# Evaluating the efficacy of the HITSystem 2.1 to improve PMTCT retention and maternal viral suppression in Kenya: Study protocol of a cluster-randomized trial

**DOI:** 10.1371/journal.pone.0263988

**Published:** 2022-07-26

**Authors:** Sharon Mokua, May Maloba, Catherine Wexler, Kathy Goggin, Vincent Staggs, Natabhona Mabachi, Nicodemus Maosa, Shadrack Babu, Emily Hurley, Sarah Finocchario-Kessler

**Affiliations:** 1 Kenya Medical Research Institute, Nairobi, Kenya; 2 Global Health Innovations–Kenya, Nairobi, Kenya; 3 Department of Family Medicine, University of Kansas Medical Center, Kansas City, Missouri, United States of America; 4 Children’s Mercy Kansas City, Health Services and Outcomes Research, Kansas City, Missouri, United States of America; 5 University of Missouri-Kansas City, School of Medicine, Kansas City, Missouri, United States of America; Public Library of Science, UNITED KINGDOM

## Abstract

**Background:**

Gaps in the provision of guideline-adherent prevention of mother-to-child transmission of HIV (PMTCT) services and maternal retention in care contribute to nearly 8000 Kenyan infants becoming infected with HIV annually. Interventions that routinize evidence-based PMTCT service delivery and foster consistent patient engagement are essential to eliminating mother-to-child transmission of HIV. The HITSystem 2.1 is an eHealth intervention that aims to improve retention in PMTCT services and viral load monitoring, using electronic alerts to providers and SMS to patients. This study will evaluate the impact, implementation, and cost-effectiveness of HITSystem 2.1.

**Method:**

This cluster randomized trial will be conducted at 12 study hospital (6 intervention, 6 control). Pregnant women living with HIV who have initiated PMTCT care ≤36 weeks gestation are eligible. Women enrolled at control hospitals will receive standard-of-care PMTCT services. Women enrolled at intervention hospitals will receive standard-of-care PMTCT services plus enhanced HITSystem 2.1 tracking. Mixed logistic regression models will compare the arms on two primary outcomes: (1) completed guideline-adherence PMTCT services and (2) viral suppression at both delivery and 6 months postpartum. We will assess associations between provider and patient characteristics (disclosure status, partner status, depression, partner support), PMTCT knowledge, and motivation with retention outcomes. Using the RE-AIM model, we will also assess implementation factors to guide sustainable scale-up. Finally, a cost-effectiveness analysis will be conducted.

**Discussion:**

This study will provide insights regarding the development and adaptation of eHealth strategies to meet the global goal of eliminating new HIV infections in children and optimizing maternal health through PMTCT services. If efficacious, implementation and cost-effectiveness data gathered in this study will guide scale-up across Kenyan health facilities.

**Trial registration:**

This study was registered at clinicaltrials.gov (NCT04571684) on October 1, 2020.

## Introduction

Prevention of mother-to-child transmission of HIV (PMTCT) programs provide services for pregnant women living with HIV through the antenatal, delivery, and postpartum phases and are critical in reducing mother-to-child transmission. All pregnant or breastfeeding women living with HIV in Kenya are eligible to receive life-long antiretroviral therapy (ART) [1].

By 2020, the proportion of pregnant Kenyan women living with HIV who were on ART had reached 94% [1]. However, a survey of records of 4 Kenyan hospitals from 2013 to 2016 indicated that only 38% of pregnant women living with HIV received ART regimens compliant with guidelines at the time [2]. Retention in care, particularly in the postpartum period, continues to pose a challenge. One study suggests 28% attrition through 6 months postpartum [3]. These Kenya data are consistent with broader literature highlighting suboptimal patient retention throughout the phases of PMTCT, including high rates of patient disengagement or loss to follow-up [4–10], poor maternal medication adherence [11–13], ART failure [14, 15], and missed opportunities for early infant diagnosis (EID) [16].

Maternal viral load (VL) levels provide a clear benchmark to guide clinical management and minimize risk of mother-to-child transmission [17]. Pregnant women with suppressed VL have very low risk of transmitting the virus to their infant. Clinical intervention to address unsuppressed maternal VL during pregnancy or breastfeeding can include enhanced adherence support, regimen switching, and enhanced infant prophylaxis [18–20]. These interventions are only feasible if eligible patients receive VL tests, results are promptly returned to health facilities, and detectable VL results lead to clinical action. Records reviewed at 4 Kenyan hospitals indicate insufficient VL testing among pregnant and postpartum women: only 72% received any VL test by 6 months postpartum and only 6% received guideline adherent baseline and repeat testing through 6 months postpartum. Furthermore, among patients who received a viral load test, few (36.0%) were notified of their results and only 28.9% had any documented evidence of clinical action taken [21]. Delays in timely clinical action after detectable VL results were cited in PEPFAR’s Kenya Country Operational 2017 Plan as a key barrier in Kenya, and as a target for interventions [22].

Interventions that foster consistent patient engagement and routinize the delivery of best practices across all phases of PMTCT are essential to prevent perinatal HIV infections. HITSystem 2.0 is a web-based intervention that tracks pregnant women living with HIV and their infants to improve the completeness and efficiency of PMTCT and EID services. In a pilot study at two Kenyan hospital (1 control, 1 intervention), HITSystem 2.0 participants were more likely to receive complete PMTCT services compared to SOC (56.6% vs. 17.1% p < 0.001); however, viral load testing at both sites was suboptimal, prompting a HITSystem modification to more comprehensively prompt and track guideline-adherence viral load testing (HITSystem 2.1) [23]. The HITSystem 2.1 intervention aims to facilitate complete PMTCT retention and VL monitoring with prompt clinical action (adherence support, ART regimen change) in the antenatal, delivery, and 6-month postpartum periods to increase viral suppression during windows critical for HIV prevention.

This study uses a cluster randomized controlled design at 12 Kenyan government hospitals with the overall goal of evaluating the HITSystem 2.1 intervention’s impact on complete, guideline-adherent PMTCT care through 6 months postpartum. HITSystem 2.1 reflects the 2018 Kenyan PMTCT guidelines [24], including added intervention features that support routine viral load monitoring and interventions to optimize maternal ART adherence. We aim to rigorously evaluate the impact of HITSystem 2.1 with the long-term goal of optimizing the provision of guideline-adherent services and viral suppression through the antenatal, delivery, and early postpartum periods.

## Materials and methods

### Study overview

**Fig 1:**
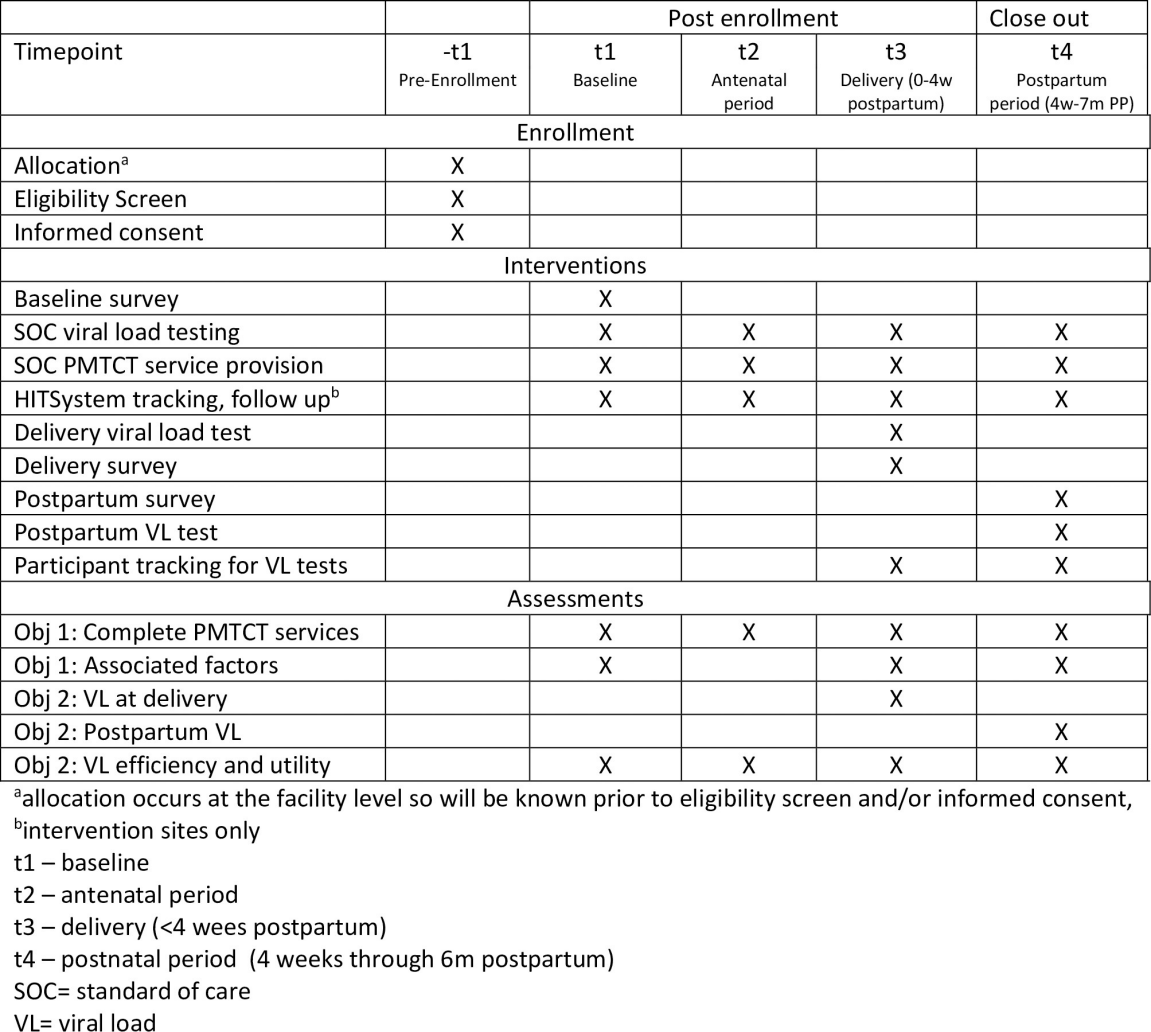
SPIRIT schedule of enrollment, interventions, and assessments.

This is a 5-year cluster-randomized control trial designed to evaluate the HITSystem 2.1 in Kenya, while also assessing implementation to guide long term sustainability (NCT04571684). There are four specific objectives that this study aims to achieve: (1) evaluate the efficacy of HITSystem 2.1 in increasing the proportion of women who receive complete PMTCT services through 6 months postpartum, per Kenyan national guidelines, (2) evaluate the efficacy of HITSystem 2.1 in increasing viral suppression (<1,000 ml/copies) through 6 months postpartum, (3) evaluate the reach, effectiveness, adoption, implementation fidelity, and maintenance (RE-AIM model) of HITSystem 2.1 using system data and surveys with providers and patients, and (4) evaluate the costs and cost-effectiveness of HITSystem 2.1 for increasing complete PMTCT retention, viral suppression, and modeled estimates for pediatric HIV infections averted, see **Fig 1** for the schedule of enrollment, interventions and assessments [25]. All study procedures were approved by Institution Review Boards at the University of Kansas Medical Center (STUDY00144753) on December 5^th^ 2019 and Kenya Medical Research Institute (SERU3983) on 27^th^ April 2020. At the time of PMTCT enrollment, all eligible women will be informed about the study by research staff, and provide written informed consent for participation.

### HITSystem 2.1 intervention

The HITSystem is accessed online through a computer or tablet, using mobile broadband modems that respond to cellular signal. There are two active modules of the HITSystem: the EID module of the HITSystem (HITSystem 1.0) combines SMS outreach to mothers of HIV-exposed infants and algorithm-based dashboard alerts to prompt provider (MCH/HIV and laboratory) action to improve retention of mother-infant pairs in early infant diagnosis of HIV (EID) services and ART initiation among HIV-infected infants [26]. The PMTCT module of the HITSystem (HITSystem 2.0) expands the scope of the intervention to involve pregnant women living with HIV, and primary intervention components include: (1) electronic prompts to notify PMTCT providers and program managers when action is required to support timely services (ART initiation, syphilis testing, linkage to EID) or when patients have missed appointments and (2) automated text messages to participants’ mobile phones with the aim of motivating adherence to medication, reminding women of ANC appointments and medication refills, and prompting preparation for a hospital delivery [23]. A recent modification to the PMTCT module (HITSystem 2.1) has increased VL test tracking and follow up and has expanded the duration of maternal follow up from 6 weeks to 6 months. Automatic linkage from the PMTCT module to the EID module allows for simplified tracking of mother-infant pairs from pregnancy through EID completion at 18 months. Table 1 outlines the progression of the HITSystem from 1.0 to 2.1.

**Table 1:** Key difference between iterations of the HITSystem.

	HITSystem 1.0	HITSystem 2.0	HITSystem 2.1
Services	EID	PMTCT	
Population	HIV-exposed infants	Pregnant women/mothers living with HIV	
Time period	6 weeks– 18 months	ANC– 6wk postpartum	ANC– 6 month postpartum
Targeted outcomes	• Efficiency of sample processing • ART initiation for HIV+ infants • Retention through 18 months	• Appointment attendance • ART adherence • Hospital delivery • Infant linkage to EID by 6wk postnatal	HITSystem 2.0, plus: • VL testing through 6m postpartum • Efficiency of VL testing • Clinical action based on VL result

EID = early infant diagnosis; PMTCT = prevention of mother-to-child transmission; VL = viral load; ART = antiretroviral therapy; ANC = antenatal care

### Site selection

The study will be conducted within twelve government health facilities in Siaya (n = 8), Kilifi (n = 2), and Mombasa (n = 2) counties of Kenya. These counties were selected due to their elevated perinatal HIV transmission rates and lower density of interventions targeting perinatal HIV transmission that could confound the results of this evaluation. To be eligible for selection and matching in the study, health facilities were required to provide PMTCT, EID, and ART services and not be involved in any ongoing PMTCT or EID interventions or research studies.

### Randomization

Randomization was conducted at the facility level. Study sites were matched on geographic region, resource level, and patient volume to produce six matched pairs. The study statistician randomly allocated one site in each pair to the intervention, using a random number generator program. The study statistician and all research staff were blinded to the randomization process. However, neither study staff nor participants will be blinded after the randomization process.

### Study staffing and training

A research assistant (RA) will be stationed at each study site and trained on tasks specific to their site designation (i.e., intervention vs control site) and will be overseen by a Kenyan study coordinator. Two study coordinators will oversee sites, based on geographic location and will be responsible for routine site visits. Table 2 outlines key responsibilities of RA and study coordinators. During the first weeks of implementation, the study coordinators will work closely with the RA and site-level clinical staff to ensure thorough understanding and accurate independent use of the HITSystem 2.1. Key hospital personnel (PMTCT and maternity nurses and mentor mothers) will be trained by employing hands-on data entry scenarios tailored to the specific capacity in which they will utilize the HITSystem 2.0.

**Table 2:** Study staffing and activities at intervention and control sites.

	Intervention site	Control site
Study Coordinator (n = 2, overseeing 12 sites)	• Provider surveys • Monitor study data • Facilitate home-based testing • Routine visits	
	• HITSystem troubleshooting/ retraining	• Electronic entry of manually recorded standard-of-care clinical data
Research Assistant (n = 12, 1 per site)	• Participant screening • Informed Consent • Surveys: enrollment, delivery, postpartum • Facilitate delivery VL tests	
	• HITSystem data entry and alert monitoring	• Support of SOC services without intervention

### Aims 1 and 2 methods

**Fig 2:**
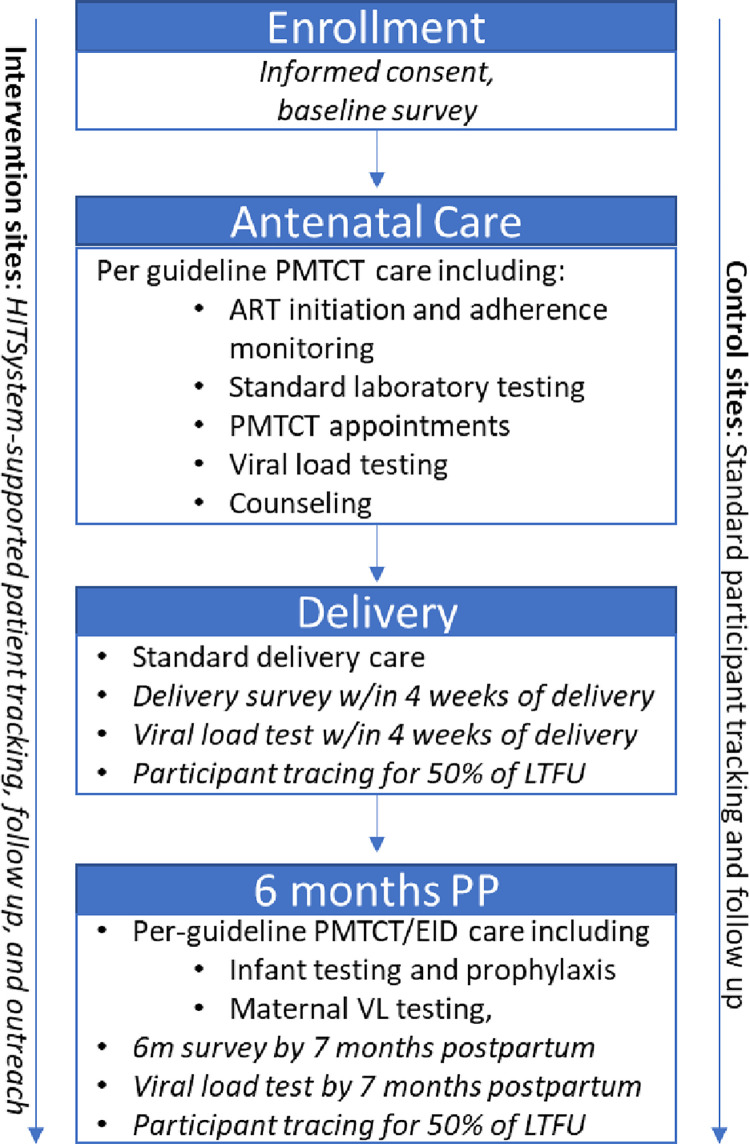
Patient flow through study services. [Normal text represents standard PMTCT services, per Kenyan guidelines. Italicized text represents study-specific services.].

Aims 1 and 2 will use the same patient population and similar methods; thus, they will be described together, see Fig 2.

#### Participant eligibility and consent

All pregnant women living with HIV presenting for PMTCT services at study hospitals by 36 weeks gestation will be eligible for enrollment. Since Kenyan law allows all pregnant females the same capacity to consent as an adult, young women under the age of 18 will be able to provide consent for themselves and will be eligible for inclusion. Women will be excluded from study participation if they have any condition (including drug abuse, alcohol abuse, or psychiatric disorder) that study or hospital staff feel precludes their ability to provide informed consent. Capacity will be determined at the time of consent. Women who transfer care from one study site to another during their PMTCT services will be ineligible for enrollment at their new facility.

#### Procedures

*Baseline surveys* will be conducted at study enrollment at both intervention and control sites. The baseline survey will collect demographic and clinical data, as well as patient-level data regarding PMTCT information, motivation, partner status and support, HIV disclosure, depression (modified Edinburgh postnatal scale [27]), and risk of intimate partner violence (Appendix 1 in S1 Appendix). Repeated surveys after delivery and 6m postpartum will include these same patient-level measures to assess potential correlates of outcomes (Appendices 2, 3 in S1 Appendix). Surveys are estimated to take about 15 minutes and participants will be compensated 200 KES for completion of each survey.

#### Clinical data collection

*At sites implementing HITSystem 2*.*1*, all patient-specific data including: demographics; women’s phone number and patient tracing information; women’s appointment attendance and ART medication refills/pill counts; dates of maternal VL sample collection, test processing, results return, and notification; maternal VL results; infant’s date of birth, birth weight, and gestational age at delivery will be entered directly into HITSystem 2.1, as they become available. At enrollment, intervention site participants will indicate their SMS preferences by opting in or out of SMS for appointment reminders (sent 2 days before scheduled appointment), delivery support (sent 4 weeks and 2 weeks prior to estimated delivery date and encourages planning for a hospital-based delivery), and ART adherence (option to select never, daily, weekly, monthly).

*At control sites*, all clinical and PMTCT service-related data will be recorded in the existing paper-based registries by health care providers as part of routine services. To prevent stimulating unintended intervention at control sites because of more comprehensive review of clinical data for research, site coordinators–rather than site-based research assistants–will collect control site data. To ensure comparable data collection tools and quality at intervention and control sites, control site data will be entered into an electronic system that mirrors the HITSystem’s data collection fields but has all HITSystem functionality (provider alerts and SMS features) disabled.

*Viral load tracking*: To understand the efficacy of HITSystem 2.1 to increase viral suppression we will systematically measure VL of all women (intervention and control) at the time of delivery (defined as within 4 weeks of delivery) and by 7 months postpartum to approximate maternal VL during the antenatal and early postnatal periods. VL testing at delivery goes beyond the guidelines and is done solely for research purposes. To ensure that this testing does not interfere with our ability to examine routine VL testing postpartum and avoid confusion regarding VL guidelines, the delivery VL test will be coordinated by study staff, processed by the CDC KEMRI laboratory (not the clinical laboratory) and results will be maintained outside of clinic files by the US-based study manager. For the postpartum VL test, results of the first routine postpartum VL test will be collected from the medical record. Per guidelines, the first postpartum VL test should occur within 6 months, so even women tested very late in pregnancy will be due for retesting by 6 months postpartum. Participants without a repeat VL test by 5 months postpartum will be tracked by study staff through phone or in-person contact to schedule a VL test by 7 months postpartum.

*Participant tracking for VL among participants ‘lost to follow-up’ (LTFU)*. Measurement of viral suppression rates in either study arm can be skewed if detectable VL is more/less frequent in LTFU patients. Therefore, we will track patients who receive no delivery VL test or postpartum VL test within the indicated time windows from a “watchlist” that was developed for each site to indicate patients in need of a time-sensitive VL test. Women without a routine VL by the indicated time will be targeted for active tracking including phone calls and home visit. Study staff conducting home visits (mentor mother and community health worker) will be thoroughly trained on protection of confidentiality, especially when participants are not home or have relocated. Visits will be introduced as well-baby visits to protect confidentiality, and a dried blood spot sample (DBS) for maternal VL will only be collected once privacy is confirmed and consent provided. Visits will be rescheduled if privacy is perceived to be compromised at any time. (Appendix 4 in S1 Appendix). Participants who re-engage in care after active tracing for viral load collection will not be eligible to be considered “completely” retained in care.

*Potential correlates of HITSystem efficacy* will be assessed through patient characteristics gathered in patient surveys at baseline, delivery, and at the postpartum viral load test. Facility assessment forms (Appendix 5 in S1 Appendix) will be completed at the beginning, mid-point and end of study implementation to assess facility factors such as average resource level, annual PMTCT volume, and PMTCT provider-patient ratios.

*Provider surveys* will be conducted at baseline and again at the end of the study period. Providers in both arms will be asked to complete a short survey (Appendix 6 in S1 Appendix) assessing provider role/ department, level of experience, knowledge and motivation regarding current PMTCT guidelines, and barriers and facilitators to provision of complete PMTCT services. Providers will be eligible to complete the survey if they (1) work primarily in ANC, PMTCT, CCC, laboratory, or MCH departments, (2) will/have been involved in the provision of PMTCT care during the course of the study, and (3) have interacted with the HITSystem 2.1 during the course of the study (intervention sites, only).

*Completion of study follow up*. Study follow-up is complete when participants receive their postpartum viral load test and complete the postpartum survey OR when they reach 7 months postpartum. Participants at both intervention and control sites will continue to receive PMTCT and EID services per their hospital’s standard of care. Participants from intervention sites can choose to continue receiving enhanced follow up for their infant through the EID module of the HITSystem. Patient clinical data after these completion points will not be documented as part of the study.

### Aim 1 outcomes and analyses

#### Aim 1 outcomes

The primary outcome for Aim 1 is receiving “complete PMTCT services,” an aggregate measure incorporating the documented receipt of all designated services across the antenatal (ART initiation, on-time appointment attendance, VL testing per guidelines, clinical action upon detectable VL), delivery (hospital-based delivery), and postpartum (EID linkage by 7 weeks, VL testing per guidelines, clinical action upon detectable VL) phases of PMTCT services. Participants who receive all indicated services per guidelines will be coded as 1 or ‘yes’. Participants missing 1 or more services will be coded as 0 or incomplete PMTCT services.

Secondary outcomes associated with Aim 1 will include gestational week at PMTCT enrollment, duration of PMTCT retention (weeks), and ART adherence (proportion >95%, proportion with missing/late prescription refill). Proportions will be calculated to describe each component of “complete PMTCT services” separately (appointment attendance, VL monitoring, hospital delivery, EID linkage). Patient and facility-level factors will be assessed as correlates of receiving complete PMTCT. Provider characteristics will be aggregated and treated as facility-level variables. We will measure provider and patient information and motivation both pre- and post-intervention to examine changes in these variables throughout the study period. We also will measure the proportion and timing/age of any HIV transmissions to infants, and maternal and/or infant deaths.

*Statistical analyses for Aim 1 (complete PMTCT services)*. Data from women who have a documented reason for incomplete care at the study hospital (e.g., transfer facility, pregnancy loss, maternal death) will not be included in analyses. To demonstrate the efficacy of HITSystem 2.1 versus standard of care, we will test for differences between arms by modeling complete PMTCT as a function of study arm and selected covariates (e.g., age, partner support, depression) using logistic mixed models with a random site intercept (to adjust for clustering at the facility level). We will explore patient and facility-level characteristics as correlates of HITSystem effectiveness. We will compute average levels of provider knowledge and motivation for each site at baseline and end of study. These knowledge and motivation scores, as well as patient knowledge and motivation levels, will be compared pre-post intervention and between study arms to assess change within and between sites. In addition, provider and patient knowledge and motivation will be examined as predictors of PMTCT completion using logistic regression.

### Aim 2 outcomes and analyses

#### Aim 2 outcomes

The primary outcome for Aim 2 is suppressed VL at both delivery and within 6 months postpartum. Secondary outcomes associated with Aim 2 will include VL test coverage (VL test in antenatal and postpartum periods, per guidelines), VL test efficiency (turn-around time from sample to result and turn-around time from result to patient notification), VL test utility (detectable VL results with clinical action per guidelines, such as: intensified adherence counseling and/or ARV regimen change), and repeated VL testing among those with detectible VL. We will document outcomes for pregnant and breastfeeding women who are LTFU to better enumerate outcomes for this population.

#### Statistical analyses for Aim 2

We will test for differences on binary outcomes between arms using logistic mixed models with adjustment for clustering and select covariates. After transformation (e.g., log) of turn-around time values, if needed, differences in time will be assessed using appropriate generalized or linear mixed models. Post hoc analyses will assess person-level characteristics as moderators of the HITSystem 2.1 effect to assess whether the HITSystem 2.1 is more effective for specific subgroups. Any differences will guide future adaptations to improve efficacy among specific populations.

### Power and sample size

Sample size calculations were carried out to ensure sufficient power to detect a difference in odds of complete PMTCT services between the intervention (p1) and control (p2). We estimated current levels of retention in complete, guideline-adherent PMTCT services based on findings from our pilot work [23]. In the HITSystem R34 pilot study, guideline-adherent PMTCT services were completed at a much higher rate at the intervention facility (11% SOC v 43% HITSystem, p = 0.002, 32 percentage point increase). When complete, guideline-adherent PMTCT is defined to include VL monitoring, completion rate estimates drop substantially. Findings from our retrospective review at 4 Kenyan health facilities indicate only 50/424 (11.8%) of women enrolled in PMTCT services received both a guideline- adherent VL test in pregnancy AND a guideline adherent retest within 6 months postpartum. We conservatively anticipate HITSystem 2.1 supported VL testing will exceed that of standard of care by at least 20 percentage points (e.g., 12% v 32%). We assumed that the small subset of participants with guideline-adherent VL testing will be included among the larger population of those receiving complete PMTCT retention. To account for increased familiarity with guidelines over time and continually improving SOC, we assessed statistical power for contingencies with SOC guideline adherence as high as 18% using R package clusterPower [28]. Assuming ICC = .05, the planned sample size of six 75-person clusters per arm provides 85% power to detect differences even smaller than the expected 20-percentage point difference across a range of possible standard of care completion rates. Using HITSystem 2.0 pilot data, we predict 28% attrition; thus, we need to enroll a minimum of n = 96 women per cluster (75 + 21). To account for possible temporal differences in enrollment, we will enroll at each pair of matched sites until n = 96 is reached at both sites of the matched pair OR until a full year of enrollment data has occurred, whichever is later.

### Aim 3 outcomes and analyses

We will evaluate the implementation of HITSystem 2.1 components to guide programmatic implementation and increase sustainable use in government health facilities. The RE-AIM framework is well suited to evaluate the implementation of system-level interventions, and outlines the public health impact as the intersection between: Reach, Effectiveness, Adoption, Implementation, and Maintenance. Table 3 outlines how each of these components of the RE-AIM model will be evaluated.

**Table 3:** HITSystem 2.1 implementation evaluation guided by the RE-AIM model.

	Definition	Measures	Data Sources
Reach	Number, proportion, representativeness of individuals who participate	• HITS 2.1 enrollment vs refusal • Patient characteristics	• Enrollment log vs hospital registers • Patient surveys
Effectiveness	The impact of an intervention on important outcomes	Aim 1 and Aim 2 primary outcomes	• HITS 2.1 • Hospital registers
Adoption	The number, proportion, representativeness of settings and intervention agents	• Provider and hospital characteristics • System Use	• Provider surveys • Facility assessment forms
Implementation	The intervention fidelity to the various elements of an intervention’s protocol	• SMS delivery/receipt • Provider engagement • Changes to HITS2.1	• SMS Log • Surveys • Field notes, IRB amendments
Maintenance	The extent to which a program or policy becomes part of routine practice	• Continued HITS 2.1 programmatic enrollment • Cost effectiveness	• HITS 2.1 vs hospital registers • Field notes

#### Statistical analyses for Aim 3

Clinical and survey data will be summarized using descriptive statistics and analyzed per Aim 1 and 2’s analysis strategy. Qualitative content of field notes (documentation of contextual events over the course of the study [i.e., strikes, reagent stock outs, laboratory machine breakdowns], email exchanges with other relevant summary documentation regarding implementation, meeting minutes of team calls) will be reviewed and coded in summary themes relevant to the implementation process and experience, per the RE-AIM model.

### Aim 4 specific methods

We will establish a unit associated with each type of resource used, estimate the dollar value of each resource, calculate the number of units used, and multiply the units of resources used by the dollar cost for each. Costs (licensing, computing/telecommunication, and personnel/training) will then be summed to derive the low, medium, and high estimates of the total cost of the intervention. Research (research personnel, remuneration) costs will be calculated separately. Cost data will be collected at the end of study years 2 & 4. We will then remove the costs saved with HITSystem 2.1 from the gross program cost. We will conduct sensitivity analyses with a range of estimates for HIV prevalence, rates of viral suppression, mother-to-child transmission rates, and patient volume to assess variations in cost effectiveness outcomes driven by key epidemiologic or demographic data.

The primary driver of the proposed cost-effectiveness analyses for HITSystem 2.1 will be the estimated reduction in the proportion of infants diagnosed with HIV at the initial test at 6-weeks of age. Since maternal nonadherence to ART, long turn-around time for VL results, and failure to take clinical action based on detectable VL results represent wasted investments in PMTCT services, secondary drivers of the cost-effectiveness analyses will be the proportions of women with optimal ART adherence during the antenatal and post-partum periods, women with VL results returned in time for prompt clinical action (by 36 weeks gestation), and detectable VL results that trigger guideline-adherent clinical action.

While the most rigorous plan is to use the number of perinatal transmissions observed in the study (intervention v control), we recognize the actual numbers are expected to be low. Thus, a secondary plan is to rely on viral suppression data in each study arm to estimate and compare perinatal transmission on a larger scale. Based on literature assessments of maternal viral load and perinatal HIV transmission of HIV [17, 29–32]. we will estimate rates of transmission at 2.5% and 22.5% for virally suppressed and unsuppressed women, respectively. We will use the estimated number of transmissions in intervention and control sites to compute savings from pediatric infections averted (lifetime ART costs for infants, HIV-related mortality). Data collected on ART adherence and VL testing will be used to calculate the wastage saved by HITSystem 2.1. VL test costing data have been assessed at the national level [33], and costing estimates for maternal and pediatric ART are available by regimen [34, 35], but outcome data from the proposed study are needed to adequately calculate the benefits of HITSystem 2.1. Anticipated costs, costs savings, and benefits are outlined in Table 4.

**Table 4:** Anticipated costs, savings and benefits for cost-effectiveness analysis.

HITSystem 2.1 Intervention Costs	Anticipated Cost Savings	Anticipated Benefits
Training, licensing, Internet, computer/modem, implementation support *costs to health system may increase with higher rates of VL testing	Reduced: delayed/unreturned VL results, detectable VL results without clinical action, poor maternal ART adherence, pediatric infections and associated costs	QALY saved (infants) QALY saved (mothers)

### Study oversight

Study-related adverse events will be reported to both overseeing IRBs, the study sponsor, and an established data and safety monitoring board (DSMB). Annual reviews by both IRBs will be conducted to ensure participant safety and compliance with study protocols. The external DSMB will be established to review and evaluate the accumulated study data for participant safety, study conduct, and progress on an annual basis. The DSMB will also conduct a blinded interim analysis after 67% of the planned sample in each arm has reached the endpoint for the primary outcomes to assess if HITSystem 2.1 efficacy can be determined for the main study outcomes: complete PMTCT retention (Aim 1) and maternal viral suppression (Aim 2) before accrual is complete. Members of the DSMB include an epidemiologist, a biostatistician, and a medical doctor with expertise in gynecology and obstetrics in global health settings. Any decisions for discontinuing or modifying allocated interventions will be discussed with the DSMB prior to implementation.

### Dissemination policy

Relevant new information on best practices for preventing perinatal HIV transmission that emerges from this study will be disseminated widely to all key stakeholders, including hospital, county, and national level PMTCT and EID administrators and policy makers in Kenya; PMTCT and EID researchers and programmers, globally; and the community of caregivers of HIV-positive pregnant and postpartum women in Kenya. We will achieve these goals with the following steps: (1) the study registration will be updated annually during the course of the study to recognize progress and report results, (2) as data become available and are analyzed, results will be widely presented at national and international conferences and through publication of manuscripts, (3) study findings will be presented at dissemination meetings in Kenya to ensure that local, Kenyan hospital, county, and national level stakeholders (patients, clinicians, county administrators, and national policy makers) are informed of key results of the research, and (4) when appropriate, de-identified data will be deposited to public repositories. Key points of this dissemination plan are included on the informed consent documents, so that participants are informed of the plan prior to agreeing to participate in the research.

## Results

We are currently in year 2 of the 5-year study period and are actively enrolling participants at both intervention and control sites. Currently, only the first round of facility assessments have been conducted, and the first round of provider surveys are complete. To date, no data have been analyzed.

## Discussion

In spite of a global concerted effort, uptake and retention in quality, evidence-based PMTCT and HIV care remain suboptimal. This study will include vulnerable populations–HIV-infected mothers and HIV-exposed or HIV-infected infants living with severely constrained resources. Participation does have the potential for direct benefit. Complete PMTCT and guideline-adherent VL monitoring and clinical management have the potential to improve HIV+ women’s health and prevent transmission of HIV to exposed infants. If transmission does occur, earlier linkage to infant HIV testing can allow for earlier treatment initiation, which has been shown to improve outcomes in HIV-infected infants. In addition, for those who miss their postpartum VL test, follow up efforts to obtain a sample and share VL results have the potential to directly benefit mother-infant pairs (especially if VL is high) by re-engaging them in care and facilitating action to address high viral load.

Findings from this study will make an important contribution to PMTCT services in Kenya and comparable low resource settings and will help address the outstanding need for evidence guiding implementation of eHealth strategies and viral suppression in this context. As Kenyan and global recommendations move toward eHealth and more stringent VL monitoring strategies, it is critical to assess the performance and cost-effectiveness of these methods in resource-constrained settings. These data will provide evidence for Ministries of Health and other organizations to consider optimal eHealth strategies to meet the global goal of eliminating new HIV infections in children through PMTCT services. We will work with participating counties and health facilities throughout the study period to develop sustainability plans beyond the course of the study. If efficacious, we will work with potential funders to facilitate the roll out of HITSystem 2.1 at health facilities throughout Kenya.

## Supporting information

S1 Checklist SPIRIT 2013 checklist: Recommended items to address in a clinical trial protocol and related documents*. (PDF)

S1 Appendix Appendices 1–6 for baseline surveys, repeated surveys, facility assessment and provider survey. (PDF)

S1 Protocol (DOCX)

S1 File IRB approval (KEMRI). (PDF)

S2 File IRB approval (KUMC). (PDF)

## Abbreviations

AIDS: Acquired immune deficiency syndrome
ANC: Antenatal care
ART: Antiretroviral therapy
CHW: Community health worker
cRCT: cluster randomized control trial
EID: Early infant diagnosis
HITSystem: HIV Infant Tracking System
HIV: Human immunodeficiency virus
IPV: Intimate partner violence
KES: Kenyan shillings
LTFU: Loss to follow up
MTCT: Mother to child transmission
PEPFAR: President’s Emergency Plan for AIDS Relief
PMTCT: Prevention of mother to child transmission
SMS: short message services
SOC: Standard of care
VL: Viral load
WHO: World Health Organization

## References

[1] UNAIDS. Country Factsheet: Kenya 2020. 2020. https://www.unaids.org/en/regionscountries/countries/kenya

[2] Pricilla RA, Brown M, Wexler C, Maloba M, Gautney BJ, Finocchario-Kessler S. Progress Toward Eliminating Mother to Child Transmission of HIV in Kenya: Review of Treatment Guidelines Uptake and Pediatric Transmission Between 2013 and 2016—A Follow Up. Maternal and Child Health Journal. 2018;22(12):1685–92. doi: 10.1007/s10995-018-2612-0 30047080 PMC6245141

[3] Fayorsey RN, Wang C, Chege D, Reidy W, Syengo M, Owino SO, et al. Effectiveness of a Lay Counselor-Led Combination Intervention for Retention of Mothers and Infants in HIV Care: A Randomized Trial in Kenya. Journal of acquired immune deficiency syndromes (1999). 2019;80(1):56–63. Epub 2018/11/07. doi: 10.1097/QAI.0000000000001882 ; PubMed Central PMCID: PMC6319592. 30399035 PMC6319592

[4] Sibanda EL, Weller IVD, Hakim JG, Cowan FM. The magnitude of loss to follow-up of HIV-exposed infants along the prevention of mother-to-child HIV transmission continuum of care: a systematic review and meta-analysis. AIDS. 2013;27(17):2787–97. doi: 10.1097/QAD.0000000000000027 . 24056068 PMC3814628

[5] Ayuo P, Musick B, Liu H, Braitstein P, Nyandiko W, Otieno-Nyunya B, et al. Frequency and factors associated with adherence to and completion of combination antiretroviral therapy for prevention of mother to child transmission in western Kenya. Journal of the International AIDS Society. 2013;16(1):17994–. doi: 10.7448/IAS.16.1.17994 . 23336727 PMC3536941

[6] Schnippel K, Mongwenyana C, Long LC, Larson BA. Delays, interruptions, and losses from prevention of mother-to-child transmission of HIV services during antenatal care in Johannesburg, South Africa: a cohort analysis. BMC infectious diseases. 2015;15:46–. doi: 10.1186/s12879-015-0778-2 . 25656597 PMC4322445

[7] Myer L, Phillips TK. Beyond "Option B+": Understanding Antiretroviral Therapy (ART) Adherence, Retention in Care and Engagement in ART Services Among Pregnant and Postpartum Women Initiating Therapy in Sub-Saharan Africa. Journal of acquired immune deficiency syndromes (1999). 2017;75 Suppl 2:S115–s22. Epub 2017/05/13. doi: 10.1097/qai.0000000000001343 . 28498180

[8] Knettel BA, Cichowitz C, Ngocho JS, Knippler ET, Chumba LN, Mmbaga BT, et al. Retention in HIV Care During Pregnancy and the Postpartum Period in the Option B+ Era: Systematic Review and Meta-Analysis of Studies in Africa. Journal of acquired immune deficiency syndromes (1999). 2018;77(5):427–38. Epub 2017/12/30. doi: 10.1097/QAI.0000000000001616 ; PubMed Central PMCID: PMC5844830. 29287029 PMC5844830

[9] Phillips T, Thebus E, Bekker LG, McIntyre J, Abrams EJ, Myer L. Disengagement of HIV-positive pregnant and postpartum women from antiretroviral therapy services: a cohort study. Journal of the International AIDS Society. 2014;17(1):19242. Epub 2014/10/11. doi: 10.7448/IAS.17.1.19242 ; PubMed Central PMCID: PMC4192834. 25301494 PMC4192834

[10] Onoya D, Sineke T, Brennan AT, Long L, Fox MP. Timing of pregnancy, postpartum risk of virologic failure and loss to follow-up among HIV-positive women. Aids. 2017;31(11):1593–602. Epub 2017/05/04. doi: 10.1097/QAD.0000000000001517 ; PubMed Central PMCID: PMC5491237. 28463877 PMC5491237

[11] Katz IT, Leister E, Kacanek D, Hughes MD, Bardeguez A, Livingston E, et al. Factors associated with lack of viral suppression at delivery among highly active antiretroviral therapy-naive women with HIV: a cohort study. Ann Intern Med. 2015;162(2):90–9. Epub 2015/01/20. doi: 10.7326/M13-2005 ; PubMed Central PMCID: PMC4299931. 25599347 PMC4299931

[12] Nsubuga-Nyombi T, Sensalire S, Karamagi E, Aloyo J, Byabagambi J, Rahimzai M, et al. Multivariate analysis of covariates of adherence among HIV-positive mothers with low viral suppression. AIDS Res Ther. 2018;15(1):9. Epub 2018/04/02. doi: 10.1186/s12981-018-0197-8 ; PubMed Central PMCID: PMC5878417. 29604955 PMC5878417

[13] Haas AD, Msukwa MT, Egger M, Tenthani L, Tweya H, Jahn A, et al. Adherence to Antiretroviral Therapy During and After Pregnancy: Cohort Study on Women Receiving Care in Malawi’s Option B+ Program. Clinical infectious diseases: an official publication of the Infectious Diseases Society of America. 2016;63(9):1227–35. Epub 2016/07/28. doi: 10.1093/cid/ciw500 ; PubMed Central PMCID: PMC5064160. 27461920 PMC5064160

[14] Myer L, Dunning L, Lesosky M, Hsiao NY, Phillips T, Petro G, et al. Frequency of Viremic Episodes in HIV-Infected Women Initiating Antiretroviral Therapy During Pregnancy: A Cohort Study. Clinical infectious diseases: an official publication of the Infectious Diseases Society of America. 2017;64(4):422–7. Epub 2016/12/09. doi: 10.1093/cid/ciw792 ; PubMed Central PMCID: PMC5849096. 27927852 PMC5849096

[15] Ngarina M, Kilewo C, Karlsson K, Aboud S, Karlsson A, Marrone G, et al. Virologic and immunologic failure, drug resistance and mortality during the first 24 months postpartum among HIV-infected women initiated on antiretroviral therapy for life in the Mitra plus Study, Dar es Salaam, Tanzania. BMC infectious diseases. 2015;15:175. Epub 2015/04/18. doi: 10.1186/s12879-015-0914-z ; PubMed Central PMCID: PMC4392730. 25886277 PMC4392730

[16] Kim MH, Ahmed S, Hosseinipour MC, Yu X, Nguyen C, Chimbwandira F, et al. Brief Report: Impact of Option B+ on the Infant PMTCT Cascade in Lilongwe, Malawi. Journal of acquired immune deficiency syndromes (1999). 2015;70(1):99–103. doi: 10.1097/QAI.0000000000000692 . 26322670 PMC4537054

[17] Myer L, Phillips TK, McIntyre JA, Hsiao NY, Petro G, Zerbe A, et al. HIV viraemia and mother-to-child transmission risk after antiretroviral therapy initiation in pregnancy in Cape Town, South Africa. HIV Med. 2017;18(2):80–8. Epub 2016/06/30. doi: 10.1111/hiv.12397 . 27353189

[18] Myer L, Essajee S, Broyles LN, Watts DH, Lesosky M, El-Sadr WM, et al. Pregnant and breastfeeding women: A priority population for HIV viral load monitoring. PLOS Medicine. 2017;14(8):e1002375. doi: 10.1371/journal.pmed.1002375 28809929 PMC5557351

[19] Nielsen-Saines K, Watts DH, Veloso VG, Bryson YJ, Joao EC, Pilotto JH, et al. Three Postpartum Antiretroviral Regimens to Prevent Intrapartum HIV Infection. New England Journal of Medicine. 2012;366(25):2368–79. doi: 10.1056/NEJMoa1108275 . 22716975 PMC3590113

[20] World Health Organization. Consolidated guidelines on the use of antiretroviral drugs for treating and preventing HIV infection Geneva, Switzerland2016 [6/11/2018]. Available from: http://apps.who.int/iris/bitstream/handle/10665/208825/9789241549684_eng.pdf?sequence=1.

[21] Sandbulte M, Brown M, Wexler C, Maloba M, Gautney B, Goggin K, et al. Maternal viral load monitoring: Coverage and clinical action at 4 Kenyan hospitals. PloS one. 2020;15(5):e0232358–e. doi: 10.1371/journal.pone.0232358 . 32469876 PMC7259657

[22] PEPFAR. Kenya Country Operational Plan (COP) 2017. 2017 2017. Report No. https://copsdata.amfar.org/SDS/2017/Kenya.pdf

[23] Finocchario-Kessler S, Brown M, Maloba M, Nazir N, Wexler C, Goggin K, et al. A Pilot Study to Evaluate the Impact of the HIV Infant Tracking System (HITSystem 2.0) on Priority Prevention of Mother-to-Child Transmission (PMTCT) Outcomes. AIDS and behavior. 2021. doi: 10.1007/s10461-021-03204-0 33709212 PMC8224224

[24] Ministry of Health, National AIDS and STI Control Program. Guidelines on Use of Antiretroviral Drugs for Treating and Preventing HIV Infection in Kenya 2018 Edition. 2018. http://cquin.icap.columbia.edu/wp-content/uploads/2017/04/ICAP_CQUIN_Kenya-ARV-Guidelines-2018-Final_20thAug2018.pdf

[25] Chan A-W, Tetzlaff JM, Altman DG, Laupacis A, Gøtzsche PC, Krleža-Jerić K, et al. SPIRIT 2013 Statement: Defining Standard Protocol Items for Clinical Trials. Annals of Internal Medicine. 2013;158(3):200–7. doi: 10.7326/0003-4819-158-3-201302050-00583 23295957 PMC5114123

[26] Finocchario-Kessler S, Gautney B, Cheng A, Wexler C, Maloba M, Nazir N, et al. Evaluation of the HIV Infant Tracking System (HITSystem) to optimise quality and efficiency of early infant diagnosis: a cluster-randomised trial in Kenya. The Lancet HIV. 2018;5(12):e696–e705. 10.1016/S2352-3018(18)30245-5. PubMed Central PMCID: PMCID: PMC6289743. 30309787 PMC6289743

[27] Cox JL, Holden JM, Sagovsky R. Detection of postnatal depression. Development of the 10-item Edinburgh Postnatal Depression Scale. Br J Psychiatry. 1987;150:782–6. Epub 1987/06/01. doi: 10.1192/bjp.150.6.782 . 3651732

[28] Kleinman K, Moyer J, Reich N, Obeng D. Package ‘clusterPower’. September 5, 2017.

[29] Ioannidis JP, Abrams EJ, Ammann A, Bulterys M, Goedert JJ, Gray L, et al. Perinatal transmission of human immunodeficiency virus type 1 by pregnant women with RNA virus loads <1000 copies/ml. J Infect Dis. 2001;183(4):539–45. Epub 2001/02/15. doi: 10.1086/318530 . 11170978

[30] Mandelbrot L, Tubiana R, Le Chenadec J, Dollfus C, Faye A, Pannier E, et al. No perinatal HIV-1 transmission from women with effective antiretroviral therapy starting before conception. Clinical infectious diseases: an official publication of the Infectious Diseases Society of America. 2015;61(11):1715–25. Epub 2015/07/23. doi: 10.1093/cid/civ578 . 26197844

[31] Contopoulos-Ioannidis DG, Ioannidis JP. Maternal cell-free viremia in the natural history of perinatal HIV-1 transmission: a meta-analysis. J Acquir Immune Defic Syndr Hum Retrovirol. 1998;18(2):126–35. Epub 1998/06/24. doi: 10.1097/00042560-199806010-00004 . 9637577

[32] Garcia PM, Kalish LA, Pitt J, Minkoff H, Quinn TC, Burchett SK, et al. Maternal levels of plasma human immunodeficiency virus type 1 RNA and the risk of perinatal transmission. Women and Infants Transmission Study Group. The New England journal of medicine. 1999;341(6):394–402. Epub 1999/08/05. doi: 10.1056/NEJM199908053410602 . 10432324

[33] Cintron C, Mudhune V, Haider R, Cogswell H, Gutierrez J, Angira F, et al. Costs of HIV Viral Load and Early Infant Diagnosis Testing in Kenya. 2017.

[34] Doherty K, Essajee S, Penazzato M, Holmes C, Resch S, Ciaranello A. Estimating age-based antiretroviral therapy costs for HIV-infected children in resource-limited settings based on World Health Organization weight-based dosing recommendations. BMC Health Serv Res. 2014;14(1):201. doi: 10.1186/1472-6963-14-201 24885453 PMC4021070

[35] Ciaranello AL, Doherty K, Penazzato M, Lindsey JC, Harrison L, Kelly K, et al. Cost-effectiveness of first-line antiretroviral therapy for HIV-infected African children less than 3 years of age. AIDS (London, England). 2015;29(10):1247–59. doi: 10.1097/QAD.0000000000000672 . 25870982 PMC4536981

