# Supplementary material for: Evaluating the efficacy of the HITSystem 2.1 to improve PMTCT retention and maternal viral suppression in Kenya: Study protocol of a cluster-randomized trial
Source: PLoS One. 2022 Jul 26;17(7):e0263988. doi: 10.1371/journal.pone.0263988 (PMC9321364; doi:10.1371/journal.pone.0263988)
Supplement: S1 Appendix — (PDF) [file pone.0263988.s002.pdf]

Appendix 1. Enrollment Survey

For interviewer to fill out:

Study ID: ______________

Date of interview__________________________

Interviewer initials_________________________

Mother Demographics

1. Mothers DOB: ________________
2. Mother phone number:____________________
3. Mother tracing information: ____
   ____
4. Date of enrollment in PMTCT services:__________________
5. Estimated delivery date (EDD) ______________________
6. Date initiated HAART _______________
7. What is your **highest level of education**?
   a. No school
   b. Partial primary
   c. Completed primary
   d. Partial secondary
   e. Completed secondary
   f. University/college
8. Who have you **disclosed your HIV status to**?
   a. No one
   b. Partner/husband
   c. 1 or more family
   d. 1 or more friends
   e. 1 or more parent
9. How do you access a mobile phone?
   a. Own phone
   b. Shared, with someone who knows HIV status
   c. Shared, with someone who doesn't know HIV status
   d. Borrow phone when needed
   e. No mobile phone access
10. Time to hospital (minutes)______________
11. Expense to hospital (roundtrip, KES)______
12. What is your **relationship status?**
    a. Single
    b. Married
    c. Unmarried, but in a relationship
    d. Separated
    e. Divorced
    f. Widowed
    g. Other, specify__________
13. What is your **living situation**?
    a. No current partner
    b. Live separate from partner
    c. Live with current partner
14. How many living children do you have?
    a. Biological____________
    b. Non-biological___________
15. Have you ever been through PMTCT and/or EID services before? ………Y / N

Partner support & Financial Control

16. What is your partner's HIV status?
    a. HIV+

b. HIV –
c. Unknown
d. No current partner

17. How did your partner react to the news of your pregnancy?
    a. Happy
    b. Neutral
    c. Unhappy
    d. Don't know
    e. No current partner
18. Who usually makes the final decision regarding how money is spent in your household?
    a. My partner, always
    b. My partner more often than me
    c. My partner and me equally
    d. Me more often than my partner
    e. Me, always.
    f. No current partner
19. Average weekly household income
    a. Less than 500 KES
    b. 500-750 KES
    c. 750-1,000 KES
    d. 1,000-2,500 KES
    e. >2,500 KES
20. I worry that I will not have enough money to reach the hospital for my own or my child's care?
    a. Strongly agree
    b. Agree
    c. Disagree
    d. Strongly Disagree

Partner Support

21. How important is it to you that your male partner supports you in each of the following ways?

	Not important at all	Not very important	Somewhat important	Very important	N/A, no partner
21a. Attend your PMTCT appointments with you?					
21b. Provide money for transport to the hospital?					
21c. Give you advice on remaining healthy while pregnant?					
21d. Help you with household responsibilities so that you can rest?					
21e. Help you with household responsibilities so that you can attend PMTCT appointments?					
21f. Remind you to take your medication?					
21g. Remind you of the importance of following doctor's instructions?					
21h. Provided money to purchase medication or vitamins?					
21i. Attend the delivery?					
21j. Provide encouragement					

22. In what ways do you expect your male partner to support you during pregnancy?

	Do not expect	Unsure	Expect	N/A – no partner
22a. Attend your PMTCT appointments with you?				
22b. Provide money for transport to the hospital?				

22c. Give you advice on remaining healthy while pregnant?				
22d. Help you with household responsibilities so that you can rest?				
22e. Help you with household responsibilities so that you can attend PMTCT appointments?				
22f. Remind you to take your medication?				
22g. Remind you of the importance of following doctor's instructions?				
22h. Provided money to purchase medication or vitamins?				
22i. Attend the delivery?				
22j. Provide encouragement				

Depression

Please check the answer that comes closest to how you have felt IN THE PAST 7 DAYS, not just today.

23. I have looked forward with enjoyment to things
    a. As much as I ever did
    b. A little less than I used to
    c. Much less than I used to
    d. Not at all
24. I have blamed myself unnecessarily when things went wrong
    a. Yes, often
    b. Yes, some of the time
    c. Not very often
    d. No, never
25. I have felt scared or panicky for no good reason
    a. Yes, quite a lot
    b. Yes sometimes
    c. No, not much
    d. No, not at all
26. I have been so unhappy that I have been crying
    a. Yes, most of the time
    b. Yes, quite often
    c. Only occasionally
    d. No, never
27. The thought of harming myself has occurred to me
    a. Yes, often
    b. Sometimes
    c. Hardly ever
    d. Never

Risk of Violence

For each of the following statements (27-30), choose the answer which best indicates how often each has occurred in the past 12 months

	Never	Rarely	Sometimes	Frequently
28. In the past 12 months, has your partner ever insulted you or made you feel bad about yourself?				
29. In the past 12 months, has your partner ever hit you, kicked you, dragged you, or beaten you up?				
30. In the past 12 months, has your partner ever hit, kicked, dragged or beaten your child?				
31. In the past 12 months, has your partner ever physically forced you to have sexual intercourse when you did not want to?				

PMTCT Knowledge

Please choose the best answer:

32. During <u>pregnancy</u>, I should receive viral load testing:
    a. Never, viral load testing is only conducted after delivery
    b. Between 1 and 2 times.
    c. At least three times.
    d. Don't know
33. During pregnancy, it is recommended that HIV+ women take ART medications only if they are feeling unwell………………………True / False
34. It is recommended that I attend a minimum of how many ANC appointments?
    a. 2
    b. 3
    c. 4
    d. 5

PMTCT Motivation and self-efficacy

Please indicated the degree to which you agree or disagree with each of the following statements.

	Strongly Disagree	Disagree	Neutral	Agree	Strongly Agree
35. I know I can maintain perfect adherence to my medication throughout my pregnancy, even when other people are around.					
36. It is very important to me that I deliver my child in a health facility, rather than at home.					
37. I am worried that I will not be able to find transport to the health facility when it is time for me to deliver my child.					
38. Other commitments – such as work, household duties, childcare – may prevent me from attending all of my ANC/PMTCT appointments.					
39. Attending all ANC/PMTCT appointments is extremely important to me.					

COVID-related questions

Please indicated the degree to which you expect COVID-19 to impact the following events.

	Strongly Disagree	Disagree	Neutral	Agree	Strongly Agree
40. I delayed coming for ANC care because of concerns related to COVID-19 exposure.					
41. I delayed coming to care because someone in my household or I was feeling unwell and did not want to risk exposing others to illness.					
42. I do not want to attend the clinic as frequently for antenatal care because I am worried about COVID-19 exposure					
43. I am worried that delivering in a hospital will expose me and my child to COVID-19					
44. Travel to and from the hospital has become more challenging (cost and/or availability of cars) as a result of COVID-19					

Thank you for your time and participation.

Appendix 2. Delivery survey (within 4 weeks, Intervention and Control Sites)

For interviewer to fill out:

Study ID: ______________

Date of interview___________________________

Interviewer initials__________________________

Delivery

1. Where did you deliver your child?
    a. Health care facility
        i. If so, please specify which one ____________________
    b. Traditional birth attendant
        i. If so, please specify location of delivery ________________
    c. Home
    d. Other
        i. Please specify_________

2. Approximately how long did you labor to deliver your child**?**
    a. < 2 hours
    b. 2-5 hours
    c. 6-12 hours
    d. > 12 hours
3. Did you or the infant experience any complications**?**
    a. yes
        i. Please explain for mother and/or infant separately: _________________________ ____
    b. no

Partner status & Disclosure

4. What is your **relationship status?**
    a. Single
    b. Married
    c. Unmarried, but in a relationship
    d. Separated
    e. Divorced
    f. Widowed
    g. Other, specify________
5. What is your **living situation**?
    a. No current partner
    b. Live separate from partner
    c. Live with current partner
6. Is your current partner the infant's father?...........Y / N
7. Who have you **disclosed your HIV status to**? Select all that apply.
    a. No one
    b. Partner/husband
    c. 1 or more family
    d. 1 or more friends
    e. 1 or more parent
8. What is your partner's HIV status?
    a. HIV+
    b. HIV –
    c. Unknown
    d. No current partner
9. Who usually makes the final decision regarding how money is spent in your household?
    a. My partner, always
    b. My partner more often than me
    c. My partner and me equally
    d. Me more often than my partner

    e. Me, always.
    f. No current partner
10. Average weekly household income
    a. Less than 500 KES
    b. 500-750 KES
    c. 750-1,000 KES
    d. 1,000-2,500 KES
    e. >2,500 KES
11. I worry that I will not have enough money to reach the hospital for my own or my child's care?
    a. Strongly agree
    b. Agree
    c. Disagree
    d. Strongly Disagree

Partner Support

12. How often *during your pregnancy* did your male partner support you in the follow ways:

	Never	Rarely	Sometimes	Often
12b. Provided money for transport to the hospital?				
12c. Gave you advice on remaining healthy while pregnant?				
12d. Helped you with household responsibilities so that you could rest?				
12e. Helped you with household responsibilities so that you could attend PMTCT appointments?				
12f. Reminded you to take your medication				
12g. Reminded you of the importance of following doctor's instructions.				
12h. Provided money to purchase medication or vitamins?				
12i. My husband gave me encouragement throughout my pregnancy				
12j. Me and my husband prayed together for a safe pregnancy and delivery				

13. Did your partner attend any PMTCT appointments with you?
    a. No, he did not attend any
    b. He attended 1
    c. He attended 2
    d. He attended 3 or more.
    e. N/A, no partner.
14. If #13 was no, what were the reasons he did not attend?
    a. He was working during my PMTCT appointments__________Yes / No
    b. He lives or works far away ____________________________Yes / No
    c. He felt unwelcomed/uncomfortable at the clinic____________ Yes / No
    d. I did not want him to attend ___________________________ Yes / No
    e. He did not want to attend ______________________________ Yes / No
    f. He had other responsibilities ________________________ yes / no
    g. Other, please specify_________________________________
15. Did your male partner provide support for your child's delivery? Please select the most accurate answer.
    a. I do not have a male partner
    b. No, my male partner did not provide any type of support for my child's delivery.
    c. My male partner provided funding for me to get to the hospital, but did not come himself.
    d. My male partner escorted me to the hospital while I was in labor.
    e. My male partner visited me in the My male partner both escorted me to the hospital while I was in labor AND visited me in the hospital after my child was born.
    f. hospital after my child was born.

Depression

Please check the answer that comes closest to how you have felt IN THE PAST 7 DAYS, not just today.

16. I have looked forward with enjoyment to things
    a. As much as I ever did
    b. A little less than I used to

c. Much less than I used to
d. Not at all

17. I have blamed myself unnecessarily when things went wrong
    a. Yes, often
    b. Yes, some of the time
    c. Not very often
    d. No, never
18. I have felt scared or panicky for no very good reason
    a. Yes, quite a lot
    b. Yes sometimes
    c. No, not much
    d. No, not at all
19. I have been so unhappy that I have been crying
    a. Yes, most of the time
    b. Yes, quite often
    c. Only occasionally
    d. No, never
20. The thought of harming myself has occurred to me
    a. Yes, often
    b. Sometimes
    c. Hardly ever
    d. Never

Risk of Violence

For each of the following statements (21-24), choose the answer which best indicates how often each has occurred in the past 12 months.

	Never	Rarely	Sometimes	Frequently
21. In the past 12 months, has your partner ever insulted you or made you feel bad about yourself?				
22. In the past 12 months, has your partner ever hit you, kicked you, dragged you, or beaten you up?				
23. In the past 12 months, has your partner ever hit, kicked, dragged or beaten your child?				
24. In the past 12 months, has your partner ever physically forced you to have sexual intercourse when you did not want to?				

PMTCT Knowledge

25. Can a mothers help prevent HIV transmission to her infant in the following ways?
    a. Taking all prescribed ARTs during pregnancy and breastfeeding....................................Yes / No / DK
    b. Delivering in a health care facility, instead of at home……………...................................Yes / No / DK
    c. Using infant formula when it is available and breastfeeding when it is not………………..Yes / No / DK
    d. Giving the infant Nevirapine………………………..Yes / No / DK
    e. Giving the infant Cotrimoxazole………………………………………………..…………...Yes / No / DK
    f. Giving baby cow's milk and eggs before 6 months of age to help him/her grow strong…Yes / No / DK

26. It is recommended that my infant receives his/her first HIV test
    a. Within 2 weeks of birth
    b. At 6 weeks of age
    c. At 6 months of age
    d. At 12 months of age
    e. One s/he stops breastfeeding
    f. Don't know

27. It is recommended that babies continue being routinely tested for HIV until when:
    a. 6 months of age
    b. 12 months of age
    c. 18 months of age
    d. 2 years of age
    e. The baby stops breastfeeding

PMTCT Motivation

For each of the following, indicate the extent to which you agree or disagree with the statement.

SD=strongly disagree, D=disagree, N=neither agree nor disagree, A= agree, SA = strongly agree.

	SD	D	N	A	SA
28. I know I can maintain perfect adherence to my medication while my baby is very young and breastfeeding.					
29. Giving my baby his/her medication is extremely important to his health					
30. Even if s/he does not want to take it, I know that I'll be able to give every dose of his/her medication					
31. It is extremely important to get routine viral load tests while my baby is still breastfeeding.					
32. Other commitments – such as work, household duties, childcare – may prevent me from getting my baby tested by 6 weeks of age.					
33. Attending all of my child's appointments is extremely important to me.					

COVID-related questions

Please indicated the degree to which you expect COVID-19 to impact the following events.

	Strongly Disagree	Disagree	Neutral	Agree	Strongly Agree
34. I was worried that delivering in a hospital woud expose me and my child to COVID-19					
35. I am worried that attending the hospital for infant testing will expose me and my child to COVID-19					
36. During pregnancy, challenges with travel to and from the hospital (cost and/or availability of cars) as a result of COVID-19 caused meto delay or miss attending some ANC or medication refill appointments					
37. During pregnancy, I delayed or missed ANC or medication refill appointments because I was feeling unwell and did not want to risk exposing others to illness.					

ENGAGEMENT WITH HITSYSTEM - **INTERVENTION ONLY**

38. Since enrolling in ANC, have you received any SMS from the hospital?
    a. No, I have not received any SMS.
    b. Yes, I have received at least one SMS from the hospital but less than 3
    c. Yes, I have received between 3 and 5 SMS
    d. Yes, I have received more than 5 SMS
39. Have you received and SMS for any of the following reasons?
    a. To check how you are doing……………………………………Yes / No
    b. To inform you of your next appointment date………………… Yes / No
    c. To remind you to take ART medications……………………… Yes / No
    d. To let you know your child was due for services………………Yes / No
    e. Asking you to return to the hospital to be informed of a test result………… Yes / No
40. If yes to any option in number 39, please answer question 40 regarding your reaction to receiving the text messages, otherwise skip to 41:
    a. I did not find the SMS helpful……………….……………………………….………..True / False
    b. I found the SMS helpful to remember to take medication……………………..…..True / False
    c. I was worried that someone would see the SMS and discover my status ……….True / False
    d. The SMS helped me feel more connected to the hospital/my care provider…….True / False
    e. The SMS helped me remember when my appointments were……………………True / False
    f. Someone other than me read one of the SMS and asked me about it……………True / False
    g. A family member or friend discovered my HIV status because of the SMS………True / False
    h. The SMS gave me important information regarding my or my infant's care………True / False
    i. The SMS motivated me to continue PMTCT and/or EID care………………………True / False
41. Please select a preference for frequency of ART adherence messages going forward:
    a. Never, please stop sending me adherence messages

b. Daily
c. Weekly
d. Every two weeks
e. Monthly

Thank you for your time and participation.

Appendix 3. Postpartum survey (Intervention and Control Sites)

For interviewer to fill out:

Study ID: _______________

Date of interview____________________________

Interviewer initials___________________________

Partner status & Disclosure

1. What is your **relationship status?**
   a. Single
   b. Married
   c. Unmarried, but in a relationship
   d. Separated
   e. Divorced
   f. Widowed
2. What is your **living situation**?
   a. No current partner
   b. Live separate from partner
   c. Live with current partner
3. Who have you **disclosed your HIV status to**?
   a. No one
   b. Partner/husband
   c. 1 or more family
   d. 1 or more friends
   e. 1 or more parent
4. What is your partner's HIV status?
   a. HIV+
   b. HIV –
   c. Unknown
   d. No current partner
5. Is your current partner the infant's father? ….. Y / N / NA
6. Who usually makes the final decision regarding how money is spent in your household?
   a. My partner, always
   b. My partner more often than me
   c. My partner and me equally
   d. Me more often than my partner
   e. Me, always.
   f. No current partner
7. Average weekly household income
   a. Less than 500 KES
   b. 500-750 KES
   c. 750-1,000 KES
   d. 1,000-2,500 KES
   e. >2,500 KES
8. I worry that I will not have enough money to reach the hospital for my own or my child's care?
   a. Strongly agree
   b. Agree
   c. Disagree
   d. Strongly Disagree

Partner Support (To be filled at 6 month VL test)

9. How often since your infant's birth has your male partner supported you in the follow ways:

	Never	Rarely	Sometimes	Frequently
9a. Attended your child's medical appointments?				
9b. Provided money for transport to the hospital?				
9c. Gave you advice on infant feeding or hygiene?				

9d. Helped you with household responsibilities so that you could rest?				
9e. Reminded you to give your child medication?				
9f. Reminded you to take your medication?				
9g. Reminded you of the importance of following doctor's instructions?				
9h. Provided money to purchase medication or vitamins?				
9i. Provide encouragement to live a healthy life?				
9j. Pray together for the health of your family?				

Depression

Please check the answer that comes closes to how you have felt IN THE PAST 7 DAYS, not just how you feel today.

10. I have looked forward with enjoyment to things
    a. As much as I ever did
    b. A little less than I used to
    c. Much less than I used to
    d. Not at all
11. I have blamed myself unnecessarily when things went wrong?
    a. Yes, often
    b. Yes, some of the time
    c. Not very often
    d. No, never
12. I have felt scared or panicky for no very good reason
    a. Yes, quite a lot
    b. Yes sometimes
    c. No, not much
    d. No, not at all
13. I have been so unhappy that I have been crying
    a. Yes, most of the time
    b. Yes, quite often
    c. Only occasionally
    d. No, never
14. The thought of harming myself has occurred to me
    a. Yes, often
    b. Sometimes
    c. Hardly ever
    d. Never

Risk of Violence

For each of the following statements (15-18), choose the answer which best indicates how often each has occurred in the past 12 months

	Never	Rarely	Sometimes	Frequently
15. In the past 12 months, has your partner ever insulted you or made you feel bad about yourself?				
16. In the past 12 months, has your partner ever hit you, kicked you, dragged you, or beaten you up?				
17. In the past 12 months, has your partner ever hit, kicked, dragged or beaten your child?				
18. In the past 12 months, has your partner ever physically forced you to have sexual intercourse when you did not want to?				

PMTCT Knowledge

19. With what frequency should HIV+ mothers with undetectable viral loads receive a viral load test while they are breastfeeding their baby?
    a. Every 3 months
    b. Every 6 months
    c. Every 12 months
    d. Only after their infant has stopped breastfeeding
20. When should HIV+ mothers stop taking their ART?
    a. Not until the infant begins eating solid food, at around 6 months.
    b. Not until the infant stops breastfeeding.
    c. Not until the infant has been confirmed HIV-negative at 18 months of age
    d. Never, the mother should continue lifelong ART for her own health.
21. It is recommended that babies continue being routinely tested for HIV until when:
    a. 6 months of age
    b. 12 months of age
    c. 18 months of age
    d. 2 years of age
    e. The baby stops breastfeeding

PMTCT Motivation

For each of the following, indicate the extent to which you agree or disagree with the statement.

SD=strongly disagree, D=disagree, N=neither agree nor disagree, A= agree, SA = strongly agree.

	SD	D	N	A	SA
22. I know I can maintain perfect adherence to my medication while my baby is very young and breastfeeding.					
23. Giving my baby his/her medication is extremely important to his health					
24. Even if s/he does not want to take it, I know that I'll be able to give every dose of his/her medication					
25. It is extremely important to get routine viral load tests while my baby is still breastfeeding.					
26. Other commitments – such as work, household duties, childcare – may prevent me from getting my baby tested by 6 weeks of age.					
27. Attending all of my child's appointments is extremely important to me.					

COVID-related questions

Please indicated the degree to which you expect COVID-19 to impact the following events.

	Strongly Disagree	Disagree	Neutral	Agree	Strongly Agree
28. I was worried that coming to the hospital for infant testing would expose me and my child to COVID-19					
29. Challenges with travel to and from the hospital (cost and/or availability of cars) as a result of COVID-19 caused me to delay or miss attending some postpartum appointments					
30. I delayed or missed postpartum appointments because I was feeling unwell and did not want to risk exposing others to illness.					

Assessing delivery VL as intervention

Please indicate the approximate dates (month and year) that you've had a VL sample collected, the results of these tests, and the reason for the test since you became pregnant. Please leave blank for any that were not collected.

31. First test since pregnancy was confirmed
    a. **Test one** date _______________________
    b. Test one results (select one)
        i. Detectable
        ii. Undetectable
        iii. I got the result but don't remember what it was
        iv. I was never informed of the result
    c. Purpose of test
        i. Routine VL test
        ii. Follow up from detectable VL
        iii. Research
        iv. I don't know
32. Second test since pregnancy was confirmed
    a. **Test two** date _______________________
    b. Test two results (select one)
        i. Detectable
        ii. Undetectable
        iii. I got the result but don't remember what it was
        iv. I was never informed of the result
    c. Purpose of test
        i. Routine VL test
        ii. Follow up from detectable VL
        iii. Research
        iv. I don't know
33. Third test since pregnancy was confirmed
    a. **Test three** date _______________________
    b. Test three results (select one)
        i. Detectable
        ii. Undetectable
        iii. I got the result but don't remember what it was
        iv. I was never informed of the result
    c. Purpose of test
        i. Routine VL test
        ii. Follow up from detectable VL
        iii. Research
        iv. I don't know
34. Forth test since pregnancy was confirmed
    a. **Test four** date _______________________
    b. Test four results (select one)
        i. Detectable
        ii. Undetectable
        iii. I got the result but don't remember what it was
        iv. I was never informed of the result
    c. Purpose of test
        i. Routine VL test
        ii. Follow up from detectable VL
        iii. Research
        iv. I don't know

Engagement with HITSystem 2.1 - **INTERVENTION ONLY**

35. Since your child was born, have you received any SMS from the hospital?
    a. No, I have not received any SMS.
    b. Yes, I have received at least one SMS from the hospital but less than 3
    c. Yes, I have received between 3 and 5 SMS
    d. Yes, I have received more than 5 SMS
36. Have you received and SMS for any of the following reasons?
    a. To check how you are doing……………………………………Yes / No
    b. To inform you of your next appointment date………………… Yes / No
    c. To remind you to take ART medications……………………… Yes / No

d. To let you know your child was due for services………………Yes / No
e. Asking you to return to the hospital to be informed of a test result………… Yes / No

37. If yes to any option in number 36, please answer question 37 regarding your reaction to receiving the text messages, otherwise skip to 38.
    a. I did not find the SMS helpful………………………………………………………..True / False
    b. I found the SMS helpful to remember to take medication…………………….…..True / False
    c. I was worried that someone would see the SMS and discover my status ……….True / False
    d. The SMS helped me feel more connected to the hospital/my care provider…….True / False
    e. The SMS helped me remember when my appointments were……………………True / False
    f. Someone other than me read one of the SMS and asked me about it……………True / False
    g. A family member or friend discovered my HIV status because of the SMS………True / False
    h. The SMS gave me important information regarding my or my infant's care………True / False
    i. The SMS motivated me to continue PMTCT and/or EID care………………………True / False
38. Please select a preference for frequency of ART adherence messages going forward:
    a. Never, please stop sending me adherence messages
    b. Daily
    c. Weekly
    d. Every two weeks
    e. Monthly

Appendix 4. Data Collection Tool For Home-based Sample Collection

For all home-visits:

1. Participant ID _______________
2. Date of visit_________________
3. Person conducting visit _____________
4. Reason for visit
    a. Birth VL test
    b. 6-month VL test
    c. Other, please specify_____________
5. How many times have study staff attempted to reach this participant through a home visit?
    a. 1
    b. 2
    c. 3
    d. 4 or more
6. Participant's status
    a. Home
    b. Not at home
    c. Moved to different location
    d. Deceased
7. Is anybody (other than the participant's infant) home?
    a. Yes, participant's partner
    b. Yes, participant's non-infant child or children
    c. Yes, participant's other family member (please specify who ___________)
    d. Yes, participant's neighbor or friend
    e. No, participant is alone
8. If YES (a, b, c, or d) to question 7: Has the participant disclosed her HIV status to all people who are currently at her home?
    a. Yes
    b. No
    c. Unknown

If anyone is home, other than the participant and her infant and she has not disclosed or her disclosure status is unknown, privacy and confidentiality may be compromised. Reschedule VL and EID test (if applicable) to ensure patient confidentiality. Proceed with "well-baby" visit (Questions 9-14).

If no-one is home, other than participant and her infant, OR she has disclosed her HIV status to everyone in the home, please proceed to questions 15 - 20

Well Baby Visit (if other person is present/confidentiality is compromised):

9. Baby's MUAC _____________cm
10. Baby's temperature ________________° Celcius
11. How is breastfeeding going?
    a. Good
    b. Fair
    c. Poor
12. Any concerns with breastfeeding? ____
    ____
    ____
13. Any concerns with baby's health? ____
    ____
    ____
14. Any concerns with your own health? ____
    ____
    ____

Home visit for VL collection (if confidentiality is secure)

15. Was VL sample collected……………………………. Y / N
    14a. If no, why not?
        a. Participant refused
        b. Other, please specify ______________
16. Was the survey completed………………. Y / N
    a. If no, why not?
        i. Participant refused
        ii. Other, please specify______________________
17. Where is the participant currently accessing HIV services for herself and her infant?
    a. At the study hospital
    b. At a different health care facility, please specify which__________________
    c. With a traditional healer
    d. Disengaged from care
    e. Other, please specify ______________________________
18. Has the infant had his/her age-appropriate HIV test sample collected at a health care facility (6 wks or 6 months)?.......... Y / N
    19. **<u>If yes to #18</u>**, which facility ___________________________
    20. **<u>If no to #18</u>**, was an EID sample collected at the home visit? ………. Y / N
        i. If no to #20, why not?
            a. Participant refused
            b. Infant was not at home
            c. Other, please specify________________________

Appendix 5. Facility Assessment Form

Date of survey_________ Conducted by: ____________

1. Facility Name ____
2. Resource level (district, county, provincial, referral, etc)__________________
3. Does the hospital have an implementing partner?
    a. If yes, who:
        i. Afya+
        ii. Walter Reed
        iii. PEPFAR
        iv. FACES
        v. USAID
        vi. Other, please specify___________________________
    b. Please describe the primary contributions of the implementing partner, especially in regards to PMTCT, EID, or HIV services at the hospital ____
    ____
    ____
4. Please indicate the number of full time (FT) and part-time (PT) doctors, nurses, mentor mothers, or other **clinical personnel** who work primarily in each of the following departments. If one person works in multiple departments, please only indicate the **primary** place of employment to avoid double-counting personnel.

		Doctor/Clinical Officer	Nurse	Mentor Mother	Other (specify)
ANC/PMTCT	FT				
	PT				
Maternity	FT				
	PT				
MCH	FT				
	PT				
CCC	FT				
	PT				

5. How many pharmacists are employed:
    a. Full time _________________
    b. Part time_________________
6. How many lab technicians are employed:
    a. Full time __________________
    b. Part time__________________

Please survey the last full **<u>3 months</u>** of paper-based registers to answer the questions 7-. For example, if this survey is being conducted in June, please survey March, April, and May.

7. How many NEW PMTCT enrollments occurred in each of the previous three months
   3 months ago _________ 2 months ago___________1 month ago_______________
8. How many patients total filled or refilled ART prescriptions in the previous 3 months (including male and female infants, children, adolescents and adults)
   3 months ago _________ 2 months ago___________1 month ago_______________
9. How many women delivered their babies in the maternity in the previous 3 months?
   3 months ago _________ 2 months ago___________1 month ago_______________
10. How many infants were enrolled in EID in the previous three months
   3 months ago _________ 2 months ago___________1 month ago_______________

Appendix 6. Provider Survey

Filled by site coordinator:

Provider ID number _________________

Hospital ___________________________

Date of interview___________________

Provider Information

1. Gender……………………………………Male / female
2. Age: _________________________________
3. Please circle the title which best describes your role at the hospital:
    a. Mentor mother
    b. Nurse
    c. Clinical officer/doctor
    d. Laboratory technician
    e. Data entry personnel
    f. Administrator
    g. Other, please specify__________________
4. What department do you work in, primarily:
    a. ANC/PMTCT
    b. Maternity
    c. MCH
    d. Laboratory
    e. CCC
    f. Rotating departments
    g. Other, please specify_______________________
5. How long have you worked in the healthcare field – at this hospital or others? ______________years

PMTCT/VL Knowledge

6. Which best describes your level of knowledge regarding the most recent national guidelines for PMTCT and VL monitoring?
    a. I am very familiar with these guidelines
    b. I am somewhat familiar with these guidelines
    c. I am somewhat unfamiliar with these guidelines
    d. I am very unfamiliar with these guidelines
7. Identify whether each of the following statements is true or false:
    a. Per Kenya national guidelines, an HIV+ pregnant women newly initiated on treatment should receive her first VL test 3 months after treatment is started…………………….True / False
    b. Per Kenya national guidelines, an HIV+ pregnant woman previously initiated on treatment should receive her first VL test 3 months after pregnancy is confirmed………True / False
    c. Per Kenya national guidelines, routine VL testing during pregnancy and breastfeeding should occur every 3 months…………………………………………………………………True / False
    d. Per Kenya national guidelines, HIV+ pregnant women who have high VL results should receive adherence counseling and then a repeat VL test after 3 months………………True / False
8. Per Kenya national guidelines, All HIV+ pregnant women should receive a minimum of how many ANC appointments?
    a. 2
    b. 3
    c. 4
    d. 5
9. Per Kenya national guidelines, all HIV-exposed infants should receive their first HIV test at the following age:
    a. Within 2 weeks
    b. 6 weeks
    c. 6 months
    d. 12 months

e. At cessation of breast feeding

Motivation

Please indicate the degree to which you agree or disagree with each of the following statements.

SD= strongly disagree, D=disagree, N=neither agree nor disagree, A=agree, SA = strongly agree

	SD	D	N	A	SA
10. I always follow national guidelines regarding PMTCT and VL monitoring for HIV+ pregnant and postpartum women.					
11. Other hospital staff always following national guidelines regarding PMTCT and VL monitoring for HIV+ pregnant women and postpartum mothers.					
12. My colleagues and I are actively trying to improve adherence to all national guidelines at the hospital level.					
13. Following national guidelines for VL monitoring among HIV+ pregnant women/new mothers is extremely important for mom and baby's well-being					
14. Sometimes, it is OK to use professional judgement to delay a VL test, even when guidelines indicate that one should be provided.					
15. Factors outside of my control make it difficult to provide guideline-adherent PMTCT and VL services to HIV+ pregnant women.					
16. I try to provide guideline-adherent PMTCT services (including ART initiation, VL testing, regimen changes, etc.) because colleagues and hospital administration expect it of me.					
17. Some aspects of the national guidelines are more important to adhere to than others					
18. I rely more often on my professional instinct and/or consultation with colleagues to inform patient care than on guidelines					
19. I believe that PMTCT/EID/VL guidelines were developed based on concrete evidence generated through rigorously conducted scientific studies.					

For questions 20 –26, indicate the level to which you agree or disagree that each of the following are barriers to providing guideline adherent PMTCT care.

SD= strongly disagree, D=disagree, N=neither agree nor disagree, A=agree, SA = strongly agree

		SD	D	N	A	SA
20.	Colleagues and hospital administration do not always support the provision of guideline-adherent PMTCT and VL services					
21.	Provider workload is too high to provide all recommended assessments for PMTCT.					
22.	Guidelines are unclear and/or too complex to follow					
23.	Mothers to not always consent and/or adhere to the guideline-adherent services that we provide					
24.	Stock outs of supplies or drugs affect the provision of PMTCT services					
25.	Patients are not adhering to PMTCT recommendations because they fear exposure to COVID-19.					
26.	Hospital level protocol changes in response to COVID-19 (e.g. longer drug dispensing intervals, recommendations to limit patient exposure, etc) prevents me and my colleauges from providing PMTCT care as recommended by the national guidelines					

27. Please describe any other barriers to providing guideline-adherent PMTCT care____

Interaction with HITSystem 2.1 (Intervention sites & End-of-Study, only)

28. On average, how many hours ***per week*** do you use the HITSystem?

a. <1 hour
b. 1-3 hours
c. 3-5 hours
d. 5-10 hours
e. >10 hours

29. On average, how many days ***per week*** do you log in to the HITSystem?
    a. 1 days
    b. 2 days
    c. 3 days
    d. 4 days
    e. 5+ days
30. In the ***past month***, have you logged into the HITSystem for any of the following reasons?
    a. To enter a new patient entry……………………………………..…..…….…………….yes / no
    b. To update data on an existing patient entry……………..……………………….………..yes / no
    c. To use alerts to identify patients who have missed services…………………………yes / no
    d. To check a patient's test result……………………………………….……………..yes / no
    e. To find patient tracing information……………………………………..………….. yes / no
    f. To access Kenya's national PMTCT/EID/VL guidelines…………………..………..yes / no
    g. To generate a report of review facility-level outcomes and data……………………..yes / no
31. Please rank each of the following HITSystem components by their level of usefulness to you, with 1 being the most useful and 5 being the least useful
    a. Patient tracking ________
    b. Accessing guidelines ________
    c. Generating reports ________
    d. Checking a lab result ________
    e. Locating a patient's clinical record ________
32. Briefly describe any challenges you experienced implementing HITSystem ______________________
    ____
    ____
    ____
