# Supplementary material for: Evaluating the efficacy of the HITSystem 2.1 to improve PMTCT retention and maternal viral suppression in Kenya: Study protocol of a cluster-randomized trial
Source: PLoS One. 2022 Jul 26;17(7):e0263988. doi: 10.1371/journal.pone.0263988 (PMC9321364; doi:10.1371/journal.pone.0263988)
Supplement: S1 Protocol — (DOCX) [file pone.0263988.s003.docx]

**Evaluating the impact of HIV Infant Tracking System(HITSystem) on retention in prevention-of-mother to child transmission services and maternal viral suppression in Siaya, Mombasa, and Kilifi Counties, Kenya**

**Principal Investigators:**

Ms. Sharon Mokua^1^, Ms. May Maloba ^2^, Dr. Sarah Finocchario-Kessler^3^

**Co investigators:**

Dr. Natabhona Mabachi^3^, Dr Kathy Goggin^4^, Dr. Vincent Staggs^4^

**Institutions**

^1^Centre for Public Health Research, KEMRI

^2^ Global Health Innovations

^3^ University of Kansas Medical Center

^4^ Children’s Mercy Kansas City

**Table of Contents**

[List of Abbreviations 4](#_Toc35428923)

[List of Appendices 4](#_Toc35428924)

[Abstract 5](#_Toc35428925)

[Lay summary 6](#_Toc35428926)

[1. Introduction/Background 7](#_Toc35428927)

[2. Justification for the study 7](#_Toc35428928)

[3.0 Research Question and Hypothesis 9](#_Toc35428929)

[4.0 Objectives 9](#_Toc35428930)

[5.0 Designs and methodology 9](#_Toc35428931)

[**Procedures and Data Management** 11](#_Toc35428932)

[Objective 1 specific methods: 12](#_Toc35428933)

[Objective 2 specific methods: 13](#_Toc35428934)

[Objective 3 specific methods: 14](#_Toc35428935)

[Objective 4 specific methods 14](#_Toc35428936)

[6.0 Data storage and management 15](#_Toc35428937)

[7.0 Timeframe/duration of the study 16](#_Toc35428938)

[8.0 Ethical considerations: human subjects 16](#_Toc35428939)

[Potential Benefits: 16](#_Toc35428940)

[Potential risks and steps taken to mitigate risks: 16](#_Toc35428941)

[*Loss of confidentiality* 16](#_Toc35428942)

[*Intimate Partner Violence (IPV)* 17](#_Toc35428943)

[*Psychological Distress.* 17](#_Toc35428944)

[*Reduction of Physical pain and/or discomfort.* 17](#_Toc35428945)

[Informed Consent 17](#_Toc35428946)

[9.0 Expected application of the results 18](#_Toc35428947)

[10.0 References 19](#_Toc35428948)

[11.0 Budget 21](#_Toc35428949)

[12.0 Budget justification 21](#_Toc35428950)

[1. Appendix I: Informed consent documents 22](#_Toc35428951)

[**3.** **Appendix II. Data collection materials** 50](#_Toc35428952)

[**English translations** 50](#_Toc35428953)

[**Appendix IIA. Enrollment Survey** 50](#_Toc35428954)

[**Appendix IIB. Delivery survey (within 4 weeks, Intervention and Control Sites)** 54](#_Toc35428955)

[**Appendix IIC. 6 Month Postpartum survey (Intervention and Control Sites)** 59](#_Toc35428956)

[**Appendix IID. Facility Assessment Form** 63](#_Toc35428957)

[**Appendix IIE. Provider Survey** 64](#_Toc35428958)

[**Appendix IIF. Data Collection Tool For Home-based Sample Collection** 67](#_Toc35428959)

[Swahili Translations 69](#_Toc35428960)

[**Kiambatisho IIB: Utafiti wa wiki ya nne baada ya kujifungua (Kituo chenye, na kisichokua na mfumo)** 73](#_Toc35428961)

[**Kiambatisho IIC. Maswali Miezi 6 Baada ya Kujifungua (Mahali mfumo utatumika, na ambapo hautatumika)** 78](#_Toc35428962)

[Luo Translations 86](#_Toc35428963)

[**Appendix IIA. Enrollment Survey** 86](#_Toc35428964)

[**Appendix IIC. 6 Month Postpartum survey (Intervention and Control Sites)** 95](#_Toc35428965)

[**Appendix IIF. Data Collection Tool For Home-based Sample Collection** 100](#_Toc35428966)

# List of Abbreviations

| AIDS | Acquired immune deficiency syndrome |
| --- | --- |
| ANC | Antenatal care |
| ART | Antiretroviral therapy |
| CHW | Community health worker |
| cRCT | cluster randomized control trial |
| EID | Early infant diagnosis |
| HITSystem | HIV Infant Tracking System |
| HIV | Human immunodeficiency virus |
| IPV | Intimate partner violence |
| KES | Kenyan shillings |
| LTFU | Loss to follow up |
| MTCT | Mother to child transmission |
| PEPFAR | President's Emergency Plan for AIDS Relief |
| PMTCT | Prevention of mother to child transmission |
| SMS | short message servies |
| SOC | Standard of care |
| VL | Viral load |
| WHO | World Health Organization |

# List of Appendices

Appendix I. Informed Consent Documents

Appendix II. Data Collection Tools

Appendix III. Certificates of Translation

Appendix IV. Role of each participating investigator

Appendix V. Biosketches of non-KEMRI Investigators

Appendix VI. Human Subjects Training Certificates of non-KEMRI investigators

# Abstract

Despite progress in providing comprehensive prevention of mother-to-child transmission of HIV (PMTCT) services, significant gaps in the timely uptake and provision of guideline-adherent services and maternal retention in care remain. Such gaps create missed opportunities for preventing mother-to-child transmission and result in nearly 6,100 infants becoming infected with HIV each year in Kenya. Effective interventions that routinize the delivery of evidence-based PMTCT services and foster consistent patient engagement are essential to close the remaining gaps and eliminate mother-to-child transmission of HIV. Building off of a successful R34 grant to develop and pilot test the HITSystem 2.0, an eHealth intervention targeting PMTCT services, the *overall goal* of this proposal is to use a cluster randomized control design at 12 Kenyan government hospitals to evaluate a modified HITSystem 2.1 intervention. HITSystem 2.1 reflects the 2018 Kenyan PMTCT guidelines, including routine viral load monitoring and interventions to suppress maternal viral load. We aim to evaluate the impact, implementation, and cost-effectiveness of HITSystem 2.1 to optimize the provision of guideline-adherent services and viral suppression through the antenatal, delivery, and early postpartum periods. If efficacious and cost-effective, HITSystem 2.1 holds strong promise for national dissemination.

# Lay summary

**Introduction:** Prevention of mother-to-child transmission (PMTCT) HIV programs, provide services for HIV+ pregnant women and mothers through the antenatal, delivery, and postpartum phases. They are critical in reducing mother-to-child transmission and preservation of the health of both mother and the child. This study has implemented an intervention to aid in fostering consistent patient engagement and routinize the delivery of best PMTCT practices across all phases of PMTCT.

**Study Relevance:** The study is aimed at evaluating the efficacy of the HITSystem 2.1 to improve complete PMTCT retention through the extended period of 6 months postpartum per the updated Kenyan guidelines, and evaluate the more rigorous outcome of maternal viral suppression

**Methodology:** The Study will cover 3 counties, Siaya, Mombasa, and Kilifi. The study population will include various population groups such as pregnant women and health service providers. The study will use quantitative methods to collect information relevant to the study objectives. Data will be collected through Participant surveys, Facility assessment forms and Provider surveys. The study will also assess the costs and cost-effectiveness of HITSystem.

**Benefits to the stakeholders and public**: Findings from this study will make an important contribution to PMTCT services in Kenya and other comparable low resource countries and will contribute to the limited data regarding the implementation of eHealth strategies and viral suppression in this context

**Approvals**: This study will be submitted to the relevant approving authorities such as SERU for ethical approvals.

# **Introduction/Background**

In 2016, an estimated 6,100 new pediatric HIV infections occurred in Kenya.^1^ Prevention of mother-to-child transmission (PMTCT) of HIV programs, which provide services for HIV+ pregnant women and mothers through the antenatal, delivery, and postpartum phases, are critical to reduce mother-to-child transmission and to preserve the health of both the mother and the child. Figure 1 outlines some of the key services provided during PMTCT care. The WHO Option B+ regimen adopted by the Kenyan government in 2013 aims to engage all pregnant or breastfeeding HIV-positive women with life-long antiretroviral therapy (ART).^2,3^ The proportion of pregnant Kenyan women on ART has reached 75-80%.^1,4^ However, a survey of records at 4 Kenyan hospitals from 2013 to 2016 indicated that only 38% of pregnant women received ART regimens compliant with Option B+ guidelines.^5^ Retention, particularly in the postpartum period, continues to pose a challenge. In a study at 10 Kenyan hospitals between 2013 and 2014, PMTCT patients receiving standard of care (SOC) had 28% attrition through 6 months postpartum.^6^ These Kenya data are consistent with broader literature highlighting suboptimal patient retention throughout the phases of PMTCT, including high rates of patient disengagement or loss to follow-up,^7-13^ poor maternal medication adherence,^14-16^, ART failure,^17,18^ and missed opportunities for early infant diagnosis (EID).^19-21^

Maternal viral load (VL) levels provide a clear benchmark to guide clinical management and minimize risk of mother-to-child transmission.^22^ Limited evidence suggests that mothers with suppressed VL have very low risk of transmitting the virus to their infant. During *pregnancy*, estimates of the risk of mother-to-child transmission range from 0% to 5% when maternal VL is <1,000 copies/mL^22-25^ and 8.5%-37% when maternal VL is >1,000 copies/mL—with higher VL associated with greater risk of transmission.^22,25,26^ During *breastfeeding*, rates of mother-to-child transmission among mothers on ART range from 0.24%-3.1%, with a pooled risk of 1.8%, and higher rates among mixed-fed infants.^27-29^ Among the few studies tracking maternal VL and mother-to-child transmission through breastfeeding, all transmitting mothers had at least one detectable plasma VL (>1000 copies/mL) during the follow up period, and/or a documented treatment interruption.^30,31^

Clinical intervention to address high maternal VL during pregnancy or breastfeeding can include enhanced adherence support, regimen switching, and enhanced infant prophylaxis.^32-34^ These interventions are only feasible if: 1) eligible patients receive VL tests, 2) results are promptly returned to health facilities, and 3) detectible VL results lead to clinical action. For women already on ART when presenting for antenatal care, Kenya’s PMTCT guidelines call for a VL test as soon as pregnancy is confirmed. Women newly diagnosed with HIV per routine testing during pregnancy receive their first VL test 3 months after ART initiation.^35^ Repeat testing occurs every 6 months.^36^ VL testing should, therefore, reach most women 1-2 times during pregnancy and 1-4 times during breastfeeding. National data on the uptake of these tests in PMTCT have not been published, but our pilot data of records reviewed at 4 Kenyan hospitals indicate insufficient VL testing among pregnant (49.8%) and breastfeeding (by 6 months postpartum) (21.7%) women, an average of 2.3 weeks’ turnaround time to get VL test results, and few patients (36.0%) being notified of their results. Only 28.9% of patients with detectable viral loads had any documented evidence of clinical action taken. Delays in timely clinical action after detectable VL results were cited in PEPFAR’s Kenya Country Operational 2017 Plan as a key barrier in Kenya, and as a target for interventions.

**Summary of HITSystem 2.1 intervention**

HITSystem 2.1 is a web-based intervention that tracks HIV+ pregnant women and their HIV-exposed infants to improve the completeness and efficiency of PMTCT and EID services. Key intervention features include: (1) **SMS messages sent to enrolled HIV+ pregnant women and mothers** to support essential PMTCT and EID services (PMTCT: appointment attendance, viral load monitoring medication adherence, hospital delivery; EID: infant HIV testing, result notification, maternal viral load testing), (2) **automated, algorithm-driven alerts** for providers when per-guidelines PMTCT and EID services are missed, (3) **laboratory-hospital linkage** to increase communication and improve tracking of maternal viral load and infant HIV DNA PCR samples, and (4) **automatic enrollment of infants into EID** and linkage with maternal PMTCT file at documentation of infant birth to improve the continuum of care for HIV+ mothers and HIV-exposed infants. The HITSystem 2.1 intervention aims to facilitate complete PMTCT retention and VL monitoring with prompt clinical action (adherence support, ART regimen change) in the antenatal, delivery, and 6-month postpartum periods to increase viral suppression during windows critical for HIV prevention.

# Justification for the study

Interventions that foster consistent patient engagement and routinize the delivery of best PMTCT practices across all phases of PMTCT are essential to prevent 6,100 new perinatal HIV infections and 4,800 AIDS-related deaths of Kenyan children each year.^1,4,37^ Similar gaps in sustained patient engagement throughout the lengthy EID cascade of care motivated our team’s development of the HITSystem 1.0. In a cluster randomized controlled trial (cRCT), this intervention demonstrated efficacy to significantly improve retention of infants enrolled in EID services through 18 months postnatal, reduce turnaround times for infant test results and notification of mothers, and increase initiation of ART at a younger infant age.^38^ In 2016, the National AIDS/STI Control Program in Kenya recognized the HITSystem 1.0 as a best practice for improving **
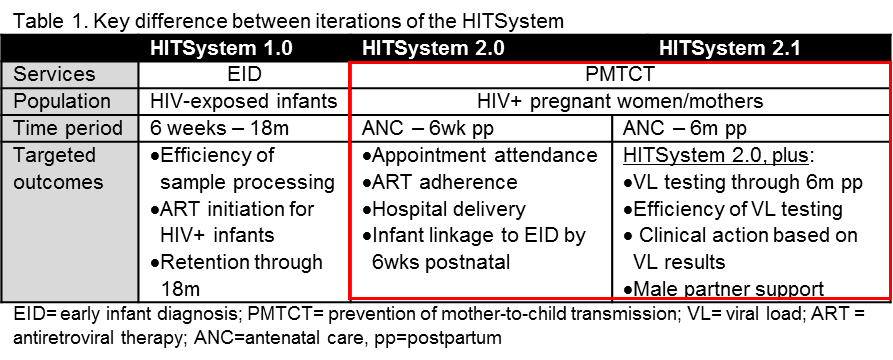
**linkage to HIV care and treatment.^39^

Leveraging this successful platform to target more efficient and complete engagement of women in PMTCT services, our team adapted the HITSystem to support provider and patient behaviors to optimize PMTCT services and outcomes and then piloted the HITSystem 2.0 at two sites [R34MH107337]. HITSystem 2.0 provides **prospective tracking** of time-sensitive interventions; with **SMS text prompts for women** to attend appointments, (content: “Tafadhali mama fika kliniki siku ya [APPOINTMENT DATE] kwa ajili ya maudhurio ya ujauzito, tunatarajia kukuona”), remain ART adherent (participant chooses content: “ni saa” OR “uhali gani?” or “Habari yako”), and plan a hospital delivery (content: “Tuna furaha sana vile uko karibu kujifungua! Ili mtoto azaliwe na afya bora kabisa, ni vizuri ujipange kujifungulia hospitalini”, as well as **electronic provider alerts** signaling missing services or poor ART drug adherence.

Figures 1A/B, below, show the HITSystem 2.1’s automated alerts (1A) and data entry tabs (1B).


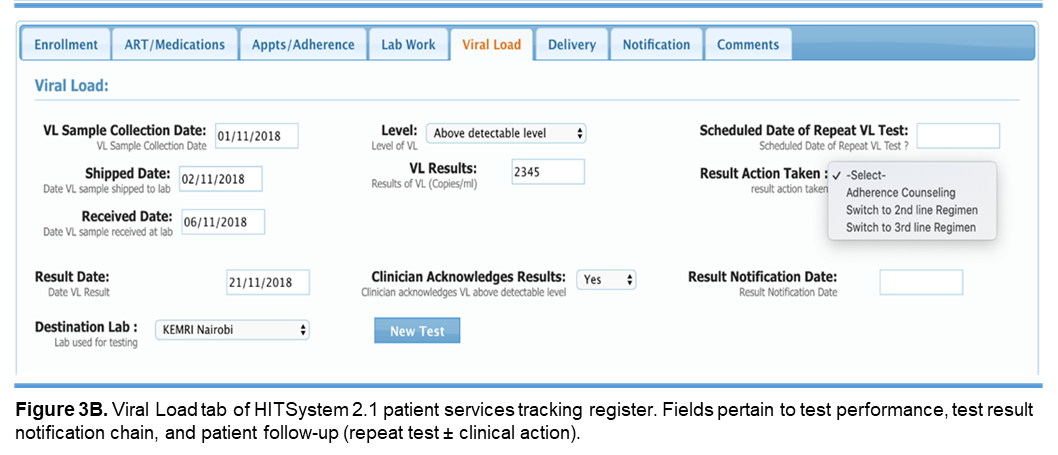

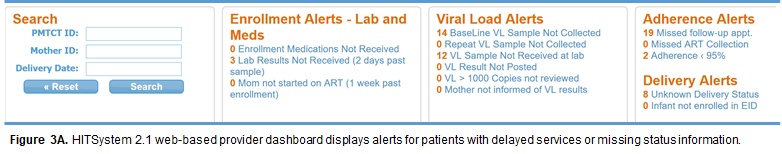


**Figure 1A**

**Figure 1B**

Resource-limited countries, including Kenya, are investing significant resources to expand VL testing during PMTCT and for all patients on ART.[40] Kenya spent $20.6 million on VL testing commodities alone in fiscal year 2015/2016.[41] Unfortunately, inefficiencies in managing VL results and failures to deliver follow-up clinical action drive down the return on investment for PMTCT VL testing. Such gaps create missed opportunities to prevent perinatal HIV transmission, negate intended maternal health benefits, and reduce cost-effectiveness of VL testing.[42] Low cost tools to facilitate guideline-adherent care allow optimization of existing investments in HIV prevention and treatment services—maximizing the benefit of known scientific advances. **This study will build upon the R34 pilot data to evaluate the efficacy of the HITSystem 2.1 to improve complete PMTCT retention, which has only been evaluated on a pilot scale. In addition, it will extend the evaluation period through 6 months postpartum per the updated Kenyan guidelines, and evaluate the more rigorous outcome of maternal viral suppression.**

# Research Question and Hypothesis

This proposal seeks to answer the research question: “Does the HITSystem 2.1 increase complete PMTCT retention with guideline-adherence services and increase maternal viral suppression through 6-months postpartum compared to standard of care PMTCT services?”

We hypothesize that the HITSystem 2.1 will improve complete PMTCT retention, improve maternal viral suppression through 6 months postpartum, and be cost-effective.

# Objectives

General Objectives: We will evaluate the impact of the HITSystem 2.1 system-level intervention in a pragmatic clinical setting on two primary outcomes: complete, guideline-adherent PMTCT retention **(Objective 1)** and viral suppression **(Objective 2)** through 6 months postpartum. We will also evaluate implementation outcomes **(Objective 3)** and the cost-effectiveness of HITSystem 2.1 **(Objective 4)**.

Specific objectives:

**Objective 1:** Evaluate the efficacy of HITSystem 2.1 to increase the proportion of women who receive complete PMTCT services through 6 months postpartum, per Kenyan national guidelines.

*Hypothesis: Pregnant women receiving HITSystem 2.1 will have a significantly higher completion rate for guideline-adherent PMTCT services compared to pregnant women receiving SOC.*

**Objective 2:** Evaluate the efficacy of HITSystem 2.1 to increase viral suppression (<1,000 ml/copies) through 6 months postpartum, per Kenyan national guidelines.

*Hypothesis: Sites implementing HITSystem 2.1 will have a significantly lower proportion of women with a detectible VL at delivery and 6 months postpartum compared to those receiving SOC.*

**Objective 3**. Evaluate *the reach, effectiveness, adoption, implementation fidelity and maintenance (REAIM model) of HITSystem 2.1 using system data and surveys with providers and patients.*

*
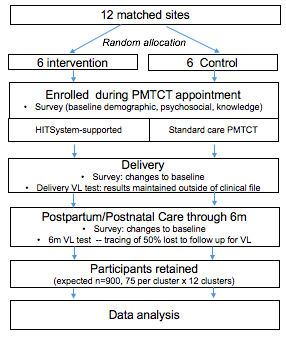
***Objective 4:** Evaluate the costs and cost-effectiveness of HITSystem 2.1 for increasing: 1) complete PMTCT retention, 2) viral suppression, and 3) modeled estimates for pediatric HIV infections averted. Cost effectiveness will be driven by estimating the proportion of women averting a detectible VL result during pregnancy or postpartum as a proxy for perinatal transmissions averted.

*Hypothesis****:*** *HITSystem 2.1 will be cost-effective for PMTCT retention and viral suppression, based on WHO criteria for life years saved.*

# Designs and methodology

Study Design: This will be a hybrid 1 study design(mixed methods), where we are testing effects of a clinical intervention on relevant outcomes while observing and gathering information on implementation.^43^ We propose a matched cluster randomized controlled trial (2 arms, 6 clusters/arm, minimum of n=75 participants per cluster), using mixed methods. We will enroll PMTCT clients at Kenyan health facilities offering either HITSystem 2.1 intervention or standard of care (SOC) services to evaluate the efficacy, implementation, and cost-effectiveness of HITSystem 2.1 to increase complete PMTCT retention and viral suppression. Participants will be enrolled in PMTCT and followed through 6 months postpartum, with focused data collection at enrollment, delivery, and 6 months postpartum, Figure 1.

1. **Study sites:** Study sites will be located in Siaya, Mombasa, and Kilifi Counties.^44^ These counties were selected due to their (1) elevated MTCT rates and (2) lower density of interventions targeting MTCT that could confound the results of this evaluation. To be eligible for selection and matching in the study, health facilities must (1) provide PMTCT, EID, and ART services and (2) not be involved in any ongoing PMTCT or EID interventions or research studies.

We will collaborate closely with the national PMTCT technical working group to facilitate strategic site selection to maximize the similarity of facilities matched on geographic region, patient volume, and HIV prevalence. Finalized matches are outlined in Table 2.

| **Table 2: Proposed matched sites.** | | |
| --- | --- | --- |
| Intervention Site | Control Site | Region |
| Mariakani | Vipingo | Coast |
| Mtwapa | Likoni | Coat |
| Bondo Referral | Siaya Referral | Siaya |
| Malanga | Yala | Siaya |
| Ukwala | Sigomere | SIaya |
| Ambira | Akala | Siaya |

Randomization: All eligible facilities will be yoked in matched pairs. The study statistician will then randomly allocate one site in each pair to the intervention. Use a random number generator program to randomly allocate one site in each pair to the intervention. The study statistician will be blind to the process of choosing the sites. Similarly, all other research staff will be blind to the randomization process.

1. **Study Population**

The primary target population for the study is HIV-positive pregnant women.

*Inclusion criteria for HIV-positive pregnant women*: All HIV-positive women presenting for PMTCT services at study hospitals during pregnancy will be eligible for enrollment in the proposed study. Pregnant women under the age of 18 are eligible for inclusion in this study. Since Kenyan law allows pregnant women the same capacity to consent as an adult, young women under the age of 18 will be able to provide consent for themselves. Mentor mothers will take additional time with young women to ensure comprehension, emphasizing that participation is optional and not a requirement for receiving standard PMTCT/EID services for themselves or their infant.

Participants who do not own a mobile phone are also eligible for study enrollment. Investigators will not provide mobile phones to women but, rather, will evaluate the efficacy of the intervention’s other components (alerts to provider for missed services) among participants without phones. Mobile phone ownership will be assessed as a mediator/moderator of intervention effectiveness.

*Exclusion criteria for HIV-positive pregnant women*: HIV-positive pregnant women will be excluded from study participation if she has any condition (including drug abuse, alcohol abuse, or psychiatric disorder) that study or hospital staff feel precludes her from providing informed consent. Capacity will be determined at the time of consent. Additionally, women who transfer care from one study site to another during their PMTCT services will be ineligible for enrollment at their new facility.

To assess mediators and moderators of HITSystem effectiveness, as well as part of the objective 4 evaluation of facility adoption, we will also interview providers at intervention and control hospitals.

*Inclusion criteria for providers*: Providers will be eligible to complete the survey if they (1) work primarily in ANC, PMTCT, CCC, laboratory, or MCH departments, (2) will/have been involved in the provision of PMTCT care during the course of the study, and (3) have interacted with the HITSystem 2.1 during the course of the study (intervention sites, only).

*Exclusion criteria for providers*: Providers will be excluded from survey eligibility if they do not provide informed consent.

1. **Sampling**

All HIV-positive pregnant women who present for care at study hospitals will be eligible for enrollment in the study. All eligible study candidates will be informed of the purpose of the research, including potential benefits and risks, prior to a request for written informed consent (Appendices 2A and 2B, see detailed informed consent section below). Women opting out of the study at intervention sites will have the option to either (1) access standard PMTCT services without enrollment in HITSystem 2.1, or (2) access HITSystem 2.1-supported PMTCT without their data being included in study analyses. Women opting out of the study at control sites will receive standard PMTCT services with their data being excluded from study analyses. Mentor mothers will document study enrollment and enrollment refusal at all sites.

Providers participating in interviews will be purposively sampled to represent a range of clinical departments and roles involved in the provision of PMTCT services.


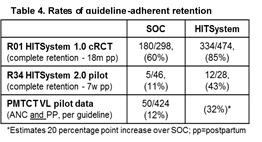


Table 3. Rates of guideline-adherent retention

Sample size determination for Objective 1: We estimated current levels of retention in complete, guideline-adherent PMTCT services based on findings from our R34 grant. Findings from our retrospective review at 4 Kenyan health facilities indicate only 50/424 (11.8%) of women enrolled in PMTCT received guideline adherent viral load testing. Based on average percentage point increases in complete retention achieved in previous studies (33% HITSystem 2.0 pilot [R34], 25% HITSystem 1.0 [R01]), we conservatively anticipate HITSystem 2.1 supported VL testing will exceed that of SOC by at least 20 percentage points (Table 3). We assume that the small subset of participants with guideline-adherent VL testing will be included among the larger population of those receiving complete PMTCT retention. To account for increased familiarity with guidelines over time and continually improving SOC, we assessed statistical power for contingencies with SOC guideline adherence as high as 18% using R package *clusterPower* ^45^. Assuming ICC=.05, the planned sample size of six 75-person clusters per arm provides 85% power to detect differences even smaller than the expected 20-percentage point difference across a range of possible SOC completion rates (Table 4). Using HITSystem 2.0 pilot data, we predict 28% attrition; thus, we need to en**roll a minimum of n=96 women per cluster** (75 + 21). To account for possible temporal differences in enrollment, we will enroll at each pair of matched sites until n=96 is reached at both sites of the matched pair OR until a full year of enrollment data has occurred, whichever is later.


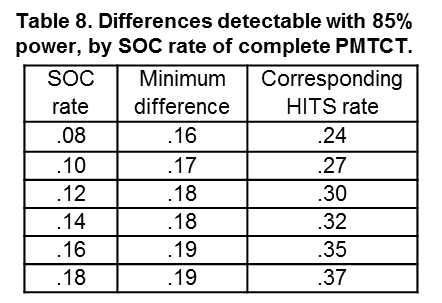


Table 4. Differences detectable with 85% power, by SOC rate of complete PMTCT

Sample size considerations for Objective 2. We draw upon limited pilot data, given the dearth of published data describing VL among women in pregnancy and in the postpartum periods, or estimates of VL of women who have disengaged from care in either period. Among PMTCT clients who received at least one VL test result, 31.3% of women in the control site had a detectible VL. Albeit small numbers, in the R34 intervention site which received intensified PMTCT retention support, but no prompts for VL testing or clinical action, 2/22

(9.1%) women had a detectible VL >1,000. These proportions reflect women engaged in clinical care during the study period. We estimate that women LTFU will have at least a 50% increased risk of a detectible VL given likely treatment interruption. In the control site, 24% of women were LTFU by the time of delivery, thus when averaging estimates for all women (retained and LTFU) we estimate 35% of women enrolled in SOC PMTCT will have a detectible VL compared to 10.2% (rounded up to 11%) in the HITSystem 2.1 sites (Table 5). With the strengthened VL component of HITSystem 2.1, we believe this estimate of 11% or less is realistic for the intervention arm. Using the R package *clusterPower*,^45^ we estimated power to detect a difference in proportions of detectible VL of .35 and .11. In an effort to demonstrate superiority of HITSystem 2.1 before accrual is complete, we will carry out an interim analysis after accruing 67% of the planned sample size in each arm. Splitting α evenly between this interim analysis and the final analysis to preserve the overall α-level at .05, power to detect the hypothesized .35 vs. .11 difference in the interim analysis at α=.025 is 95%. For a smaller difference of .30 vs. .11, power is 83% for the interim. Including the final analysis provides additional power. With the same estimates for attrition as detailed in Objective 1, the same number of participants are required for Objectives 1 & 2 (n= 75/cluster).


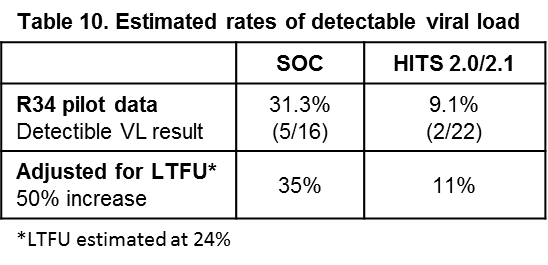


**Table 5. Estimated rates of detectable viral load**

Given the clustered nature of the data, we analyzed power for tests of differences between proportions using the R package clusterPower,^46^ which uses simulation and generalized linear mixed modeling to estimate power for cluster-randomized trials, including those with a binary outcomes.^45^ Analyses for objectives 1 and 2 will use the same patient population; thus, no new participants will be enrolled for objective 2.

Objectives 3 will assess implementation outcomes and is not statistically powered. Women and providers included in surveys will be drawn from the existing study population, i.e. no new participants will be recruited for this objective.

Objective 4 does not require human subjects.

## **Procedures and Data Management**

*Staffing and responsibilities.* We will hire and train one full time mentor mother for each study hospital. Each mentor mother will be trained to conduct study procedures specific to the study arm allocation. At all sites, mentor mothers will assess eligibility, conduct informed consent procedures and surveys, and facilitate VL testing at the time of delivery and by 6 months postpartum if not retested prior. At sites implementing HITSystem 2.1, mentor mothers will be the primary persons to enter and routinely update patient data. Mentor mothers also will lead patient outreach and follow-up efforts (phone or texting via HITSystem 2.1) and coordinate with community health workers (CHW) for targeted physical tracking if needed for VL testing at research-specific time points. Site Coordinators will routinely visit each study hospital to provide supportive supervision to ensure adherence to study protocols.

*Description of procedure and data collection:* At sites implementing HITSystem 2.1, all patient-specific data including: infant and maternal demographics; women’s phone number and patient tracing information; women’s appointment attendance and ART medication refills/pill counts; infant’s date of birth, birth weight, and gestational age at delivery; dates of maternal VL sample collection, test processing, results return, and notification; and maternal VL results will be entered directly into HITSystem 2.1. ART adherence will be measured by pill counts collected at each consultation via HITSystem 2.1. At SOC sites, all clinical and PMTCT service related data will be collected in the existing paper-based registries by health care providers as part of routine services. Because routine pill counts for ART adherence are well outside of SOC practice and could modify behaviors in the control arm, we will rely on pharmacy refill records and chart review for any provider notes related to adherence. During their monthly site visits, site coordinators will review hospital registries at SOC sites to document patients’ PMTCT engagement in study logs.

Participant surveys: A brief enrollment survey will collect same demographic and clinical data at control sites (without HITSystem 2.1) in addition to patient-level data regarding PMTCT information, motivation, partner status and support, HIV disclosure, depression (modified Edinburgh postnatal scale^47^), and risk of violence (both arms, Appendix 2A). Repeated surveys after delivery and 6m postpartum (for VL sample collection described in Objective 2, both arms) will include these same patient-level measures to assess any changes that may mediate or moderate intervention impact (Appendices 2B & 2C). Participants will be compensated 200 KES for completion of each survey.

Facility assessment form (Appendix 2D) will be completed at the beginning, mid-point and end of study implementation to assess average resource level, annual PMTCT volume, and PMTCT provider-patient ratios.

Provider surveys: At baseline and again at the end of the study period, providers in both arms will be asked to complete a short survey (Appendix 2E) assessing provider role/ department, level of experience, knowledge and motivation regarding current PMTCT guidelines, and barriers and facilitators to provision of complete PMTCT services. We estimate n=5 providers per site to complete the survey for a total of n=60 providers (n=30 intervention, n=30 control). Prior to completing the survey, providers will complete an informed consent (Appendix 2C). Providers will be compensated 500 KES for completion of each survey.

*Supportive Supervision Visits* will occur monthly at all sites. During these visits, Site Coordinators will provide technical assistance and retraining, review study materials (informed consent forms, surveys), and review data for completeness and accuracy. The monthly frequency of site visits approximates programmatic HITSystem 1.0 implementation support in Kenya.

### Objective 1 specific methods:

**Evaluate the efficacy of HITSystem 2.1 to increase the proportion of womens who receive complete PMTCT services through 6 m postpartum, per Kenyan guidelines.**

Objective 1 outcomes. The primary outcome for Objective 1 is the proportion receiving “complete PMTCT,” an aggregate measure incorporating the documented receipt of all

| Table 6. Aim 1 primary outcome: Services required for “complete PMTCT” | | |
| --- | --- | --- |
| Antenatal | Delivery | Postpartum (through 6m) |
| - ART initiation - On-time appointment attendance - VL testing per guidelines - Clinical action upon detectable VL | Hospital-based delivery | - EID linkage by 7 weeks - VL testing per guidelines - Clinical action upon detectable VL |

designated services across the 3 PMTCT phases: maternal ART initiation, antenatal appointment attendance, facility delivery, EID linkage by 7 weeks of age, maternal VL testing and clinical action per national guidelines through 6 months postpartum (Table 6). Participants who receive all indicated services per guidelines will be coded as 1 or ‘yes’. Participants missing > 1 service will be coded as 0 or incomplete PMTCT services.

Secondary outcomes associated with Objective 1 will include gestational week at PMTCT enrollment, duration of PMTCT retention (weeks), and ART adherence (proportion >95%, proportion with missing/late prescription refill). Proportions will be calculated to describe each component of “complete PMTCT” separately (appointment attendance, VL monitoring, hospital delivery, EID linkage). Patient and facility-level factors will be assessed for their moderating impact on complete PMTCT. Provider characteristics will be aggregated and treated as facility-level variables. To assess pathways through which the HITSystem 2.1 works, we will measure provider and patient information and motivation as potential mediators both pre- and post-intervention, and across study arms. Preterm births and infant weight at birth will be documented. We also will measure the proportion and timing/age of any HIV transmissions to infants, and maternal and/or infant deaths.

*Statistical analyses for objective 1 (complete retention).* Data from women who have a documented reason for incomplete care at the study hospital (e.g. transfer facility, pregnancy loss, maternal death) will not be included in analyses. To demonstrate the efficacy of HITSystem 2.1 versus SOC, we will test for differences between arms by modeling complete PMTCT as a function of study arm and selected covariates (e.g., age, partner support, depression) using logistic mixed models with a random site intercept (to adjust for clustering). In post hoc analyses we will explore patient and facility-level characteristics as potential moderators of the effect of HITSystem 2.1, and examine changes in key individual level factors (disclosure status, partner status, depression, partner support) at the midpoint (delivery) and again at the end (6m postpartum). To assess mediating factors, we will compute average levels of provider knowledge and motivation for each site at baseline and end of study. These knowledge and motivation scores, as well as patient knowledge and motivation levels, will be compared pre-post intervention and between study arms to assess change within and between sites. In addition, provider and patient knowledge and motivation will be examined as predictors of PMTCT completion using logistic regression.

### Objective 2 specific methods:

Evaluate the efficacy of HITSystem 2.1 to increase viral suppression (<1,000 ml/copies) through 6 months postpartum, per Kenyan national guidelines

To understand the efficacy of HITSystem 2.1 to increase viral suppression we will systematically measure VL of all women (intervention and control) at the time of delivery and by 6 months postpartum for those missing a repeat postpartum VL to approximate maternal VL during the antenatal and early postnatal periods.


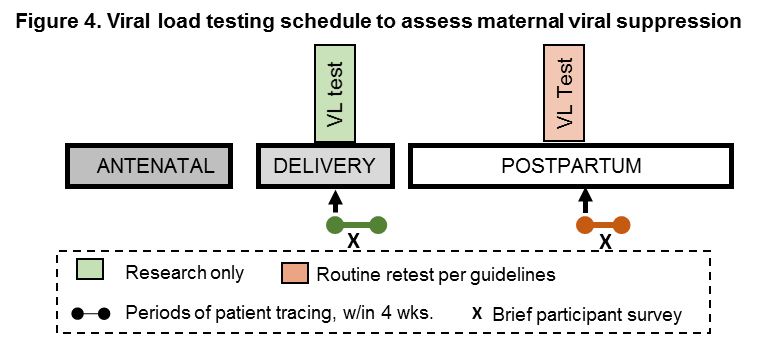


**Figure 1. Viral load testing schedule to assess maternal viral suppression**

*Tracking Viral Load.* The HITSystem 2. 1 intervention’s impact on maternal viral suppression rates will be assessed through VL tests at delivery and at each woman’s first routine postpartum VL test. VL testing at delivery goes beyond the guidelines and is done solely for research purposes. To ensure that this testing does not interfere with our ability to examine routine VL testing postpartum and avoid confusion regarding VL guidelines, maternal VL testing at delivery (within 4 weeks of delivery) will be coordinated by study staff, processed by a qualified research laboratory (not the clinical laboratory) and results maintained outside of clinic files. This additional VL test for research purposes only will be described to study candidates during informed consent. Results of the first routine postpartum VL test will be collected from the medical record. Per guidelines, the first postpartum VL test should occur within 6 months, so even women tested very late in pregnancy will be due for retesting by 6 months postpartum. Participants without a repeat VL test by 6 months postpartum will be tracked by study staff through phone or in-person contact to schedule a VL test within a 4-week window, i.e. before 7 months postpartum.

*Participant tracking for VL among participants ‘lost to follow-up’ (LTFU)***.** Measurement of viral suppression rates in either study arm can be skewed if detectible VL is more/less frequent in LTFU patients. Therefore, we will use a weighted sampling-based approach^48^ to track patients who receive no delivery VL test or postpartum VL test within 6 months. From a twice monthly LTFU patient list for each site, a random subset of 1 in 2 participants will be targeted for active tracking including phone calls and home visit to reach women who disengaged from care prior to or by the time of delivery, and thus had no VL sample collected at the time of delivery. The same strategies will be used to reach women who did not receive a postpartum VL test; either because they were not offered a test or because they disengaged from PMTCT care during this period. Study staff conducting home visits (mentor mother and community health worker) will be thoroughly trained on protection of confidentiality, especially when participants are not home or have relocated. Visits will be introduced as well-baby visits to protect confidentiality, and a dried blood spot sample (DBS) for VL will only be collected once privacy is confirmed and consent provided. Visits will be rescheduled if privacy is perceived to be compromised at any time. Women whose infant has not yet tested for HIV will be offered sample collection for infant testing, and DBS will be transported for processing (Appendix 2F, Home Visit Survey).

*Objective 2 Outcomes*. The primary outcome for Objective 2 is the proportion of clients with a detectable VL at delivery and within 6 months postpartum. Secondary outcomes associated with Objective 2 will include VL test coverage (proportion with VL test in antenatal and postpartum periods, per guidelines), VL test efficiency (turn-around time from sample to result, and turn-around time from result to patient notification), VL test utility (proportion of detectable VL results with clinical action per guidelines, such as: intensified adherence counseling and/or ARV regimen change), and repeated VL testing among those with detectible VL. We will document outcomes for pregnant and breastfeeding women who are LTFU to better enumerate outcomes for this population.

*Statistical analyses for Objective 2***.**  We will test for differences on binary outcomes between arms using logistic mixed models with adjustment for clustering and select covariates. After transformation (e.g., log) of turn-around time values if needed, differences in time will be assessed using appropriate generalized or linear mixed models. Post hoc analyses will assess person-level characteristics as moderators of the HITSystem 2.1 effect to assess whether the HITSystem 2.1 is more effective for specific subgroups. Any differences will guide future adaptations to improve efficacy among specific populations. The same methods described for mediation analysis of information and motivation will be applied in Objective 2.

### Objective 3 specific methods:

**Evaluate *the reach, effectiveness, adoption, implementation fidelity and maintenance (REAIM model) of HITSystem 2.1 using system data and surveys with providers and patients.***

We will evaluate the implementation of HITSystem 2.1 components to guide programmatic implementation and increase sustainable use in government health facilities. The RE-AIM framework is well suited to evaluate the implementation of system-level interventions, and outlines the public health impact as the intersection between: Reach, Effectiveness, Adoption, Implementation, and Maintenance, Table 6.

*Reach*: We will assess the proportion of eligible participants enrolled in HITSystem 2.1 at intervention sites by comparing HITSystem 2.1 enrollment with paper-based PMTCT registers. Reasons for non-participation will be documented for all eligible participants not included in the study. Among participants, data collected through the patient enrollment surveys will be used to describe characteristics of the sample and compared with national estimates to assess representative- ness to national population of HIV+ pregnant women.

*Effectiveness*: Objectives 1 & 2 assessing complete PMTCT retention and viral suppression will evaluate effectiveness. We will also measure adverse outcomes as a result of the program.

*Adoption*: At the *facility-level*, we will note any facilities which decline study participation and reasons for refusal. Among participating hospitals, the facility assessment form – filled out pre-mid-post study implementation – will collect data regarding resource level, patient-provider ratios, annual volume, implementing partners and outside support, and other facility details will be used to assess representativeness with Kenyan health care facilities. At the *provider-level*, adoption will be evaluated through provider surveys, assess the degree to which providers used HITSystem 2.1, determine which components were most beneficial, and assess facilitators and barriers to HITSystem 2.1 adoption. *Patient surveys* will similarly assess utility of SMS outreach components and suggestions to optimize benefit.


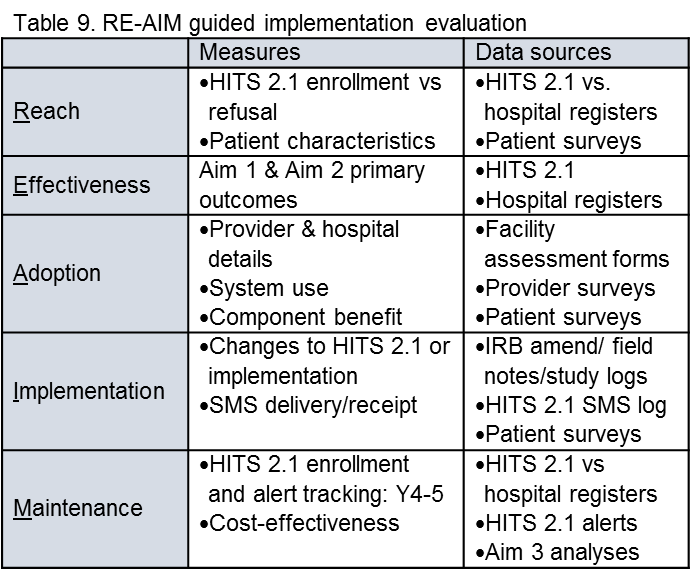


**Table 7. RE-AIM guided implementation evaluation**

*Implementation*: Any changes to HITSystem 2.1 programming or study protocols will documented in research logs and/or through IRB amendments. Any changes to HITSystem 2.1 implementation at the hospital-level (i.e. location changes of desktop to better suit workflow, changes in key users, etc) will also be documented in study logs to guide implementation recommendations. We will review all HITSystem texting logs during the implementation period to assess fidelity of the SMS component by calculating the proportion of SMS sent according to participant preference (correct frequency and content) and the proportion received by a functioning mobile number. We will compare this with participants’ reports of the number of SMS received as part of the study and the reasons for these SMS, which will be collected as part of the patient surveys.

*Maintenance*: Continued HITSystem 2.1 enrollment after the study enrollment period has ended (years 3-5) and utilization after the study follow up period (year 4-5) will serve as an indicator of system maintenance. In Years 3-5, the US-based study manager will compare the overall number of HITSystem 2.1 enrolments with PMTCT registers to assess if reach is being maintained. Using the HITSystem 2.1-generated alerts report, s/he

will document the number and duration of provider alerts as a measure of system use.

Statistical analyses for Objective 3: Survey data will be summarized using descriptive statistics to calculate proportions, medians, and mean values. The content of field notes (documentation of contextual events over the course of the study [i.e., strikes, reagent stock outs, laboratory machine breakdowns], email exchanges with other relevant summary documentation regarding implementation, meeting minutes of team calls) will be reviewed and coded in summary themes relevant to the implementation process and experience, per the RE-AIM model. These qualitative data will augment the quantitative survey data from provider and patient interviews.

### Objective 4 specific methods

Evaluate the costs and cost-effectiveness of HITSystem 2.1 for increasing: 1) complete PMTCT retention, 2) viral suppression, and 3) modeled estimates for pediatric HIV infections averted

We will establish a unit associated with each type of resource used, estimate the dollar value of each resource, calculate the number of units used, and multiply the units of resources used by the dollar cost for each. Costs (licensing, computing/telecommunication, and personnel/training) will then be summed to derive the low, medium, and high estimates of the total cost of the intervention.  Research (research personnel, remuneration) costs will be calculated separately. We will then remove the costs saved with HITSystem 2.1 from the gross program cost. We will conduct sensitivity analyses with a range of estimates for HIV prevalence, rates of viral suppression, mother-to-child transmission rates, and patient volume to assess variations in cost effectiveness outcomes driven by key epidemiologic or demographic data.

The primary driver of the proposed cost-effectiveness analyses for HITSystem 2.1 will be the estimated reduction in the proportion of infants diagnosed with HIV at the initial test at 6-weeks of age. Since maternal nonadherence to ART, long turn-around time for VL results, and failure to take clinical action based on detectable VL results represent wasted investments in PMTCT services, secondary drivers of the cost-effectiveness analyses will be the proportion of (1) women with optimal ART adherence during the antenatal and post-partum periods, (2) women with VL results returned in time for prompt clinical action (by 36 weeks gestation) and (3) detectable VL results that trigger guideline-adherent clinical action.

We will use the number of perinatal transmissions observed in the study (intervention v control), but since the actual numbers are expected to be low, we will also rely on viral suppression data to estimate transmission on a larger scale. Based on literature assessments of maternal viral load and perinatal HIV transmission of HIV ^22-26^, we will estimate rates of transmission at 2.5% and 22.5% for virally suppressed and unsuppressed women, respectively. We will use the estimated number of transmissions in intervention and control sites to compute savings from pediatric infections averted (lifetime ART costs for infants, HIV-related mortality). Data collected on ART adherence and VL testing will be used to calculate the wastage saved by HITSystem 2.1. VL test costing data have been assessed at the national level,^49^ and costing estimates for maternal and pediatric ART are available by regimen,^50,51^ but outcome data from the proposed study are needed to adequately calculate the benefits of HITSystem 2.1. Anticipated costs, costs savings, and benefits are outlined below, Table 8.

**Table 8. Anticipated costs, savings and benefits for cost-effectiveness analysis**


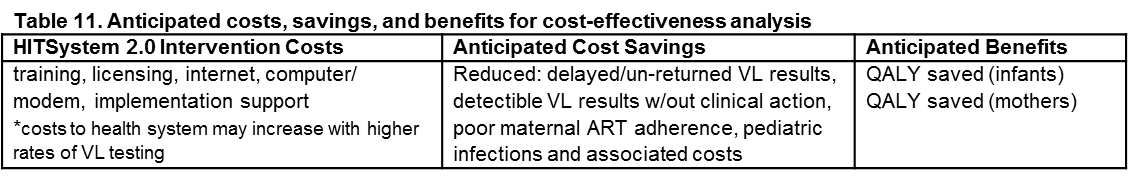


# Data storage and management

All relevant clinical data at intervention sites will be captured through the HITSystem’s online, automated system which produces output in Excel format. Patient specific information (including infant and mother demographics; mother’s phone number and patient tracing information; PMTCT appointment attendance, maternal viral load testing and results, ART adherence, infant’s date of birth; infant weight and gestational age at delivery; dates of sample collection, HIV test processing, results return, mother notification; and date of infant ART initiation for positive infants) will be entered by the Mentor Mother at the hospital during the first and subsequent PMTCT consultations to capture relevant demographic and clinical data and laboratory results. *Data will be de-identified* and maintained for each mother-infant pair within the password-protected HITSystem using a numeric ID to protect the identity of all participants. The site coordinators will routinely visit HITSystem sites for data quality assurance. At non-HITSystem sites, we will use individual patient (mother and infant) files, augmented by available hospital paper-based registers and the national laboratory online database to collect relevant routine clinical data. Patient records will be filled out by personnel performing the respective actions. The site coordinator will routinely visit non-HITSystem study sites to conduct retrospective record review of patient records and enter relevant study data into a secured electronic database. As with HITSystem data, each mother-infant pair will be identified by a numeric study ID to protect their identity.

At enrollment, at the “delivery” VL test, and at the postpartum VL test, participants will take a brief survey assessing individual characteristics, partner support, depression, and interpersonal violence. Mentor mothers will conduct these surveys on paper forms. Site coordinators will then enter the responses from paper forms into a *secured electronic database*. Additional paper-based study logs kept at study sites will document study enrollment, enrollment refusal, adverse events that occur as a result of the study, and communication between clinical or study staff and participants. Hospital or national-level events that may impact clinical care and study operations will be documented across all sites (intervention and control). In addition, separate study logs will be used to document data related to research-specific viral load sample collection at both intervention and control sites at delivery, and at >7 months postpartum for those disengaged from care, including VL sample collection date and results. Logs will be filled out by personnel performing the respective actions. The site coordinator will collect study logs and surveys routinely at all study sites and enter them into a *secured electronic database*.

All dried blood spot samples collected for research-related viral load tests will be labeled with the participant’s unique study ID and sent to an independent laboratory (KEMRI CDC) for sample processing. All samples will be processed per the laboratory’s established procedures. Results will be mailed back to the study team and maintained in a secure electronic database.

All study staff will be trained to promote standardized and objective collection and recording of participant information. Study personnel will review data entered in the HITSystem and other study data collection tools for legibility, consistency, and completeness. All data will be stored in a password-protected database that is backed up through a secure offsite connection. All HITSystem data (PMTCT and EID) will be stored and backed-up on secure servers in Kansas City, USA. We are currently in the process of identifying a Kenya-based server for local data storage. Every 24-hours study data will be backed up on a *secure cloud-based server*, which the PIs from each institution and key study personnel (biostatistician and study manager) will have access to through *password-protected login*. County officials, hospital administrators and MCH departments will be able to access de-identified progress and performance data from their facility/region by using their read only, site-specific login and password. All data collection, storage, and use will be compliant with Kenya’s Data Protection Act of 2019.^52^

# Timeframe/duration of the study

We anticipate the study will last for five years, including a 6-8 month start up period for site selection and matching, IRB approvals, hiring and training, followed by a 1.5-year period to accrue the minimum sample size of n=96 eligible women per site. This 1.5-year accrual period will allow lower volume facilities (i.e. those with 4-5 HIV-positive pregnant women enrolling per month) to achieve the sample size. If a site fails to reach the minimum of n=96 participants after 18 months, enrollment will be extended and the “maintenance monitoring” period at that facility will be shortened to achieve the target sample size. Prospective follow-up of all mother-infant pairs (pregnancy through postpartum) and provider interviews will require an additional year, after enrollment stops. Data cleaning and analyses will begin midway through Year 4, with dissemination at end of Year 5, see Table 9.


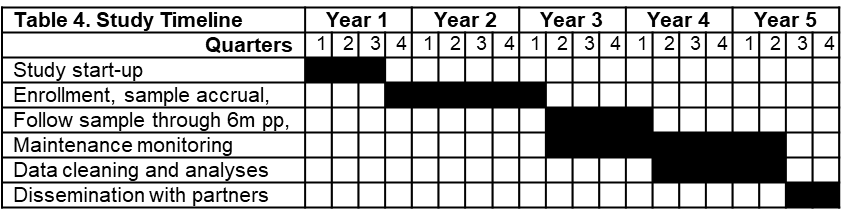


**Table 9. Study Timeline**

# Ethical considerations: human subjects

## Potential Benefits:

In spite of a global concerted effort, uptake and retention in quality, evidence-based PMTCT and HIV care continues to be suboptimal. This study will include vulnerable populations – HIV-infected mothers and HIV-exposed or HIV-infected infants living with severely constrained resources. Participation does have the potential for direct benefit. Complete PMTCT and guideline-adherence VL monitoring and clinical management has the potential to improve HIV+ women’s health and prevent transmission of HIV to their infants. If transmission does occur, earlier linkage to infant HIV testing can allow for earlier treatment initiation, which has been shown to improve outcomes in HIV-infected infants. In addition, for those who miss their postpartum VL test, follow up efforts to obtain a sample and share VL results has the potential to directly benefit mother-infant pairs (especially if VL is high) by re-engaging them in care and facilitating action to address high viral load.

## Potential risks and steps taken to mitigate risks:

*Loss of confidentiality.* To protect confidentiality, the following steps will be taken: Study staff will be trained regarding the need to strictly protect confidentiality; this will also be reinforced with the clinic staff. Prior to starting the study, we will meet with clinic staff and peer counselors to identify any additional strategies that could be taken in that particular setting to help improve participant confidentiality. This includes strict adherence to protocols for the content of HITSystem text messages sent to caregivers and procedures for those engaged in patient tracing in the community. Extensive measures have been taken in the development of the HITSystem to customize text messages to avoid risk of disclosing the caregivers’ or their infants’ HIV status or increased stigma. These steps included focus groups to help guide the development of the messages and pilot testing the content of text messages. Messages sent by the HITSystem never indicate that the hospital visit is related in any way to HIV. Access to HITSystem will be restricted only to research staff directly involved in implementation and evaluation of the study. All electronic data will be de-identified, and recorded through use of a numeric ID for each participant (HIV+ woman and her HIV-exposed or HIV-positive infant participating in Objectives 1 and 2. Access to the HITSystem will be password protected and only permitted after completion of training. The link between participant name and study ID and HITSystem ID is known only by the research staff member involved in participant enrollment or the PMTCT/EID provider who has an established care relationship with the woman and her infant (Objectives 1&2 HIV+ mothers/infants). The record linking patient name, study ID, and HITSystem ID will be securely stored by the provider in the password protected computer designated only for study use. De-identified data will be used for all analyses and results.

Home visits to track LTFU participants for VL testing present an additional risk of inadvertent HIV status disclosure to community or household members. Study staff conducting home visits will be trained on protection of confidentiality, including instructions to reschedule any home visit if privacy is perceived to be compromised. In the event a participant relocated or is not home, any conversation with household members will only refer to a well-baby visit. A “home visit” checklist will be used to reinforce these procedures.

Participants will be informed of these conditions during the informed consent process and the precautions described above will be employed to mitigate any risks to participant confidentiality, privacy, and safety.

*Intimate Partner Violence (IPV).* If a case of IPV is reported during study engagement (per routine surveys including questions to assess risk and experience of IPV), the participant will be counseled on options for seeking care and referred to the nearest service provider, including referral for legal action. Participants who report IPV over the phone (via study contact or mentor mother) will be encouraged to visit the clinic for an in-person consultation and will also be offered a home visit by a mentor mother. All study staff will be trained to ask for and identify signs of IPV among pregnant and postpartum women living with HIV. Any form or IPV during the study period will be reported as an adverse event.

*Psychological Distress.* The informed consent process will be carefully designed to prepare women for the Early Infant Diagnosis testing process after they deliver and the possibility of an HIV positive diagnosis for their infants. In addition, the existing use of peer counselors to support pregnant women and mothers to consider care and treatment options for themselves and their infants, and to process their and their infant’s HIV positive diagnosis will continue to be employed in the study. If additional support and follow-up services are needed, the PMTCT/EID provider will arrange for additional on-site counseling.

*Reduction of Physical pain and/or discomfort.* Dried blood spot (DBS) samples will be collected for all research-related viral load testing. Providers are most experienced with this form of blood sample collection and thus may be more proficient in collecting samples in this manner with less pain and discomfort for the participant. Training on VL testing procedures will be reinforced to minimize risk of collection errors and need for repeat sample collection.

# Informed Consent

All participants will be informed of the purpose of the research, including the potential benefits and risks of PMTCT/EID engagement and VL testing and, at HITSystem-implementing sites, the unique communication features of the HITSystem. The Mentor Mother will go over the informed consent with each woman and then allow her to read it over on her own. Mentor mothers will explain that all participants have the right to discontinue participation at any time and that women who decline study participation will access standard of care PMTCT/EID services. At HITSystem study sites, caregivers declining study participation can also choose to be tracked in the HITSystem without providing additional samples for VL tests

Prior to a request for written informed consent, women will be asked to summarize what they’ve been told/read to ensure comprehension. Women will then be asked if they would like to enroll by: a) providing their cell phone contact and/or tracing information from the hospital to their residence to facilitate follow up, b) allowing their and their infant’s patient information to be entered and tracked in the HITSystem (at HITSytem sites only), and c) consenting to receive research-specific VL testing at delivery. Women who are non-literate will have the option of using a thumb-print in lieu of a signature for study consent. Pregnant women under the age of 18 are eligible for inclusion in this study. Since Kenyan law allows pregnant women/mothers the same capacity to consent as an adult, young women under the age of 18 will be able to provide consent for themselves. Mentor mothers will take additional time with these young women to ensure comprehension, emphasizing that participation is optional and is not a requirement for receiving standard of care PMTCT/EID services for themselves or their infant.

The contact number of the Study Coordinator and in-country PI will be provided to all who agree to participate. Participants will be encouraged to call this number if they have questions about their participation in the study. IRB approval will be sought from KEMRI and the University of Kansas Medical Center.

# Expected application of the results

Findings from this study will make an important contribution to PMTCT services in Kenya and other comparable low resource countries and will contribute to the limited data regarding the implementation of eHealth strategies and viral suppression in this context. As Kenyan and global recommendations move toward eHealth and more stringent viral load monitoring strategies, it is critical to assess the performance and cost-effectiveness of these methods in resource-constrained settings. This data will provide evidence for Ministries of Health and other organizations to consider optimal mHealth strategies to meet the global goal of eliminating new HIV infections in children through PMTCT services. We will work with participating counties throughout the study period to develop sustainability plans beyond the course of the study. If efficacious, we will work with potential funders to facilitate the roll out of HITSystem 2.1 at health facilities throughout Kenya.

# References

1. *AIDSinfo Country Factsheets: Kenya*: UNAIDS;2016.

2. *Consolidated Guidelines on the Use of Antiretroviral Drugs for Treating and Preventing HIV Infection*: World Health Organization;2013.

3. Odeny TA, Bailey RC, Bukusi EA, et al. Effect of text messaging to deter early resumption of sexual activity after male circumcision for HIV prevention: a randomized controlled trial. *Journal of acquired immune deficiency syndromes (1999).* Feb 1 2014;65(2):e50-57.

4. National AIDS Control Council. Kenya AIDS Response Progress Report 20162016.

5. Pricilla RA, Brown M, Wexler C, Maloba M, Gautney BJ, Finocchario-Kessler S. Progress Toward Eliminating Mother to Child Transmission of HIV in Kenya: Review of Treatment Guidelines Uptake and Pediatric Transmission Between 2013 and 2016-A Follow Up. *Matern Child Health J.* Jul 25 2018.

6. Fayorsey RN, Wang C, Chege D, et al. Effectiveness of a Lay Counselor-Led Combination Intervention for Retention of Mothers and Infants in HIV Care: A Randomized Trial in Kenya. *J Acquir Immune Defic Syndr.* Jan 1 2019;80(1):56-63.

7. Sibanda EL, Weller IV, Hakim JG, Cowan FM. The magnitude of loss to follow-up of HIV-exposed infants along the prevention of mother-to-child HIV transmission continuum of care: a systematic review and meta-analysis. *AIDS.* Nov 13 2013;27(17):2787-2797.

8. Ayuo P, Musick B, Liu H, et al. Frequency and factors associated with adherence to and completion of combination antiretroviral therapy for prevention of mother to child transmission in western Kenya. *J Int AIDS Soc.* Jan 2 2013;16:17994.

9. Schnippel K, Mongwenyana C, Long LC, Larson BA. Delays, interruptions, and losses from prevention of mother-to-child transmission of HIV services during antenatal care in Johannesburg, South Africa: a cohort analysis. *BMC Infect Dis.* Feb 6 2015;15:46.

10. Myer L, Phillips TK. Beyond "Option B+": Understanding Antiretroviral Therapy (ART) Adherence, Retention in Care and Engagement in ART Services Among Pregnant and Postpartum Women Initiating Therapy in Sub-Saharan Africa. *J Acquir Immune Defic Syndr.* Jun 1 2017;75 Suppl 2:S115-S122.

11. Knettel BA, Cichowitz C, Ngocho JS, et al. Retention in HIV Care During Pregnancy and the Postpartum Period in the Option B+ Era: Systematic Review and Meta-Analysis of Studies in Africa. *J Acquir Immune Defic Syndr.* Apr 15 2018;77(5):427-438.

12. Phillips T, Thebus E, Bekker LG, McIntyre J, Abrams EJ, Myer L. Disengagement of HIV-positive pregnant and postpartum women from antiretroviral therapy services: a cohort study. *J Int AIDS Soc.* 2014;17:19242.

13. Onoya D, Sineke T, Brennan AT, Long L, Fox MP. Timing of pregnancy, postpartum risk of virologic failure and loss to follow-up among HIV-positive women. *AIDS.* Jul 17 2017;31(11):1593-1602.

14. Katz IT, Leister E, Kacanek D, et al. Factors associated with lack of viral suppression at delivery among highly active antiretroviral therapy-naive women with HIV: a cohort study. *Ann Intern Med.* Jan 20 2015;162(2):90-99.

15. Nsubuga-Nyombi T, Sensalire S, Karamagi E, et al. Multivariate analysis of covariates of adherence among HIV-positive mothers with low viral suppression. *AIDS Res Ther.* Mar 31 2018;15(1):9.

16. Haas AD, Msukwa MT, Egger M, et al. Adherence to Antiretroviral Therapy During and After Pregnancy: Cohort Study on Women Receiving Care in Malawi's Option B+ Program. *Clin Infect Dis.* Nov 1 2016;63(9):1227-1235.

17. Myer L, Dunning L, Lesosky M, et al. Frequency of Viremic Episodes in HIV-Infected Women Initiating Antiretroviral Therapy During Pregnancy: A Cohort Study. *Clin Infect Dis.* Feb 15 2017;64(4):422-427.

18. Ngarina M, Kilewo C, Karlsson K, et al. Virologic and immunologic failure, drug resistance and mortality during the first 24 months postpartum among HIV-infected women initiated on antiretroviral therapy for life in the Mitra plus Study, Dar es Salaam, Tanzania. *BMC Infect Dis.* Apr 8 2015;15:175.

19. Kim MH, Ahmed S, Hosseinipour MC, et al. Brief Report: Impact of Option B+ on the Infant PMTCT Cascade in Lilongwe, Malawi. *J Acquir Immune Defic Syndr.* Sep 1 2015;70(1):99-103.

20. Herce ME, Mtande T, Chimbwandira F, et al. Supporting Option B+ scale up and strengthening the prevention of mother-to-child transmission cascade in central Malawi: results from a serial cross-sectional study. *BMC Infect Dis.* Aug 12 2015;15:328.

21. Namukwaya Z, Barlow-Mosha L, Mudiope P, et al. Use of peers, community lay persons and Village Health Team (VHT) members improves six-week postnatal clinic (PNC) follow-up and Early Infant HIV Diagnosis (EID) in urban and rural health units in Uganda: A one-year implementation study. *BMC Health Serv Res.* Dec 15 2015;15:555.

22. Myer L, Phillips TK, McIntyre JA, et al. HIV viraemia and mother-to-child transmission risk after antiretroviral therapy initiation in pregnancy in Cape Town, South Africa. *HIV Med.* Feb 2017;18(2):80-88.

23. Ioannidis JP, Abrams EJ, Ammann A, et al. Perinatal transmission of human immunodeficiency virus type 1 by pregnant women with RNA virus loads <1000 copies/ml. *J Infect Dis.* Feb 15 2001;183(4):539-545.

24. Mandelbrot L, Tubiana R, Le Chenadec J, et al. No perinatal HIV-1 transmission from women with effective antiretroviral therapy starting before conception. *Clin Infect Dis.* Dec 1 2015;61(11):1715-1725.

25. Contopoulos-Ioannidis DG, Ioannidis JP. Maternal cell-free viremia in the natural history of perinatal HIV-1 transmission: a meta-analysis. *J Acquir Immune Defic Syndr Hum Retrovirol.* Jun 1 1998;18(2):126-135.

26. Garcia PM, Kalish LA, Pitt J, et al. Maternal levels of plasma human immunodeficiency virus type 1 RNA and the risk of perinatal transmission. Women and Infants Transmission Study Group. *N Engl J Med.* Aug 5 1999;341(6):394-402.

27. Muluye D, Woldeyohannes D, Gizachew M, Tiruneh M. Infant feeding practice and associated factors of HIV positive mothers attending prevention of mother to child transmission and antiretroviral therapy clinics in Gondar Town health institutions, Northwest Ethiopia. *BMC Public Health.* Mar 26 2012;12:240.

28. Becquet R, Bland R, Leroy V, et al. Duration, pattern of breastfeeding and postnatal transmission of HIV: pooled analysis of individual data from West and South African cohorts. *PLoS One.* Oct 16 2009;4(10):e7397.

29. Becquet R, Ekouevi DK, Menan H, et al. Early mixed feeding and breastfeeding beyond 6 months increase the risk of postnatal HIV transmission: ANRS 1201/1202 Ditrame Plus, Abidjan, Cote d'Ivoire. *Prev Med.* Jul 2008;47(1):27-33.

30. Davis NL, Miller WC, Hudgens MG, et al. Maternal and Breastmilk Viral Load: Impacts of Adherence on Peripartum HIV Infections Averted-The Breastfeeding, Antiretrovirals, and Nutrition Study. *J Acquir Immune Defic Syndr.* Dec 15 2016;73(5):572-580.

31. Luoga E, Vanobberghen F, Bircher R, et al. No HIV transmission from virally suppressed mothers during breastfeeding in rural Tanzania. *J Acquir Immune Defic Syndr.* May 16 2018.

32. Myer L, Essajee S, Broyles LN, et al. Pregnant and breastfeeding women: A priority population for HIV viral load monitoring. *PLoS Med.* Aug 2017;14(8):e1002375.

33. Nielsen-Saines K, Watts DH, Veloso VG, et al. Three postpartum antiretroviral regimens to prevent intrapartum HIV infection. *N Engl J Med.* Jun 21 2012;366(25):2368-2379.

34. *Consolidated guidelines on the use of antiretroviral drugs for treating and preventing HIV infection: recommendations for a public health approach – 2nd ed.*: World Health Organization;2016.

35. Mwenda R, Fong Y, Magombo T, et al. Significant Patient Impact Observed Upon Implementation of Point-of-Care Early Infant Diagnosis Technologies in an Observational Study in Malawi. *Clin Infect Dis.* 2018;67(5):701-707.

36. Guidelines for Prevention of Mother-to-Child Transmission (PMTCT) of HIV and AIDS in Kenya, 5th Edition. In: Ministry of Health NASCP, ed: National AIDS and STI Control Program (NASCOP); 2016.

37. Wagner A, Slyker J, Langat A, et al. High mortality in HIV-infected children diagnosed in hospital underscores need for faster diagnostic turnaround time in prevention of mother-to-child transmission of HIV (PMTCT) programs. *BMC Pediatr.* Feb 15 2015;15:10.

38. Finocchario-Kessler S, Gautney B, Cheng A-L, et al. Evaluation of the HIV Infant Tracking System (HITSystem) to optimise quality and efficiency of early infant diagnosis: a cluster-randomised trial in Kenya. *The Lancet HIV.* 2018 2018;5(12):e696-e705.

39. Improving the Quality and Efficiency of Health Services in Kenya: A Practical Handbook for HIV Managers and Service Providers on Differentiated Care. Nairobi, Kenya: National AIDS and STI Control Program (NASCOP); 2016.

40. Roberts T, Cohn J, Bonner K, Hargreaves S. Scale-up of Routine Viral Load Testing in Resource-Poor Settings: Current and Future Implementation Challenges. *Clin Infect Dis.* Apr 15 2016;62(8):1043-1048.

41. PEPFAR. *Kenya Country Operational Plan (COP) 2017 Strategic Direction Summary*: U.S. President's Emergency Plan for AIDS Relief;2017.

42. Barnabas RV, Revill P, Tan N, Phillips A. Cost-effectiveness of routine viral load monitoring in low- and middle-income countries: a systematic review. *J Int AIDS Soc.* Nov 2017;20 Suppl 7.

43. Curran GM, Bauer M, Mittman B, Pyne JM, Stetler C. Effectiveness-implementation hybrid designs: combining elements of clinical effectiveness and implementation research to enhance public health impact. *Med Care.* 2012;50(3):217-226.

44. National AIDS Control Council. Kenya HIV County Profiles2016.

45. Reich NG, Myers JA, Obeng D, Milstone AM, Perl TM. Empirical Power and Sample Size Calculations for Cluster-Randomized and Cluster-Randomized Crossover Studies. *PloS one.* 2012;7(4):e35564.

46. Kleinman K, Moyer J, Reich N, Obeng D. Package ‘clusterPower’. September 5, 2017.

47. Cox JL, Holden JM, Sagovsky R. Detection of postnatal depression. Development of the 10-item Edinburgh Postnatal Depression Scale. *Br J Psychiatry.* Jun 1987;150:782-786.

48. Geng EH, Emenyonu N, Bwana MB, Glidden DV, Martin JN. Sampling-based approach to determining outcomes of patients lost to follow-up in antiretroviral therapy scale-up programs in Africa. *JAMA.* Aug 6 2008;300(5):506-507.

49. Cintron C, Mudhune V, Haider R, et al. *USAID Health Finance and Governance Project: Costs of HIV Viral Load and Early Infant Diagnosis Testing in Kenya.* Bethesda, MD2017.

50. Doherty K, Essajee S, Penazzato M, Holmes C, Resch S, Ciaranello A. Estimating age-based antiretroviral therapy costs for HIV-infected children in resource-limited settings based on World Health Organization weight-based dosing recommendations. *BMC Health Serv Res.* May 2 2014;14:201.

51. Ciaranello AL, Doherty K, Penazzato M, et al. Cost-effectiveness of first-line antiretroviral therapy for HIV-infected African children less than 3 years of age. *AIDS.* Jun 19 2015;29(10):1247-1259.

52. Republic of Kenya. The Data Protection Act, 2019. Nairobi, Kenya2019.

# Budget

Please note, all budgetary information is subject to change based on final staffing decisions and final award amount. All amounts are shown in U.S. dollars (USD, $)

Below the combined estimated study budget for years 1 through 5 and total across all five years are outlined. These funds will be distributed among the four partnering institutions (KUMC, GHI, KEMRI, CMH).

By institution, the estimated yearly breakdown of budget is outlined below.

# Budget justification

The budgeted salaries will be used to support PI and Co-I from each partnering institution including Dr. Kessler, Dr. Mabachi, Dr. Goggin, Dr. Staggs, Dr. Hurley, May Maloba, Brad Gautney, and Sharon Mokua. Salaries will also cover support staff including US-based study manager and research associate and Kenya-based study coordinators (3), research assistants (2), and finance manager. Fringe benefits were calculated from each institution’s established fringe rate.

Money budgeted for consultants will support HITSystem refinement and cost-effectiveness analysis guidance.

Money budgeted for supplies will support purchase of items and services necessary to complete the scope of the study. These items include communication, printing, computers/modems/airtime/licensing for HITSystem use, VL testing support, clinical staff support, participant remuneration, health insurance, and participant tracking funds.

Money budgeted for travel will support US-based personnel travel to/from Kenya, as well as study staff (US- and Kenya-based) travel within Kenya.

Money budgeted in the “other” category will be used to pay publishing/conference fees to ensure a wide dissemination of findings within Kenya and at international venues.

# Appendix I: Informed consent documents

Please note, the participant (intervention and control) informed consent documents will be available in English, Swahili, and Luo. The provider informed consent document will be available only in English.

**INFORMED CONSENT DOCUMENT: INTERVENTION SITE**

**Title of the Research Study:** Evaluating the HITSystem to improve PMTCT retention and maternal viral suppression in Kenya

**Investigator(s) – Local and International Collaborators:**

**Primary Investigators:** May Maloba MCH; Sharon Mokua, MSc; Dr. Sarah Finocchario Kessler, PhD, MPH

**Co-Investigators:** Natabhona Mabachi, PhD; Brad Gautney, MPH; Kathy Goggin, PhD; Vincent Staggs, PhD

**Study location:** Kenya

You are being asked to take part in a research study. The box below tells you important things you should think about before deciding to join the study. We will provide more detailed information below the box. Please ask questions about any of the information before you decide whether to participate. You may also wish to talk to others (for example, your family, friends, or your doctor) about this study, before agreeing to join.

| **Key Information for You to Consider (Not more than 500 words)** |
| --- |
| - **Voluntary Consent**. You are being asked to volunteer for a research study. It is up to you whether you choose to participate or not. There are no penalties and you will not lose anything if you decide not to join or if after you join, you decide to quit. - **Purpose**. The HITSystem is an intervention that aims to improve PMTCT and EID by sending SMS reminders to women and their providers. This study will evaluate the HITSystem’s ability to improve PMTCT care and viral suppression among pregnant and postpartum women. - **Duration.** Your participation in the study will last until approximately 6 months after your baby is born. - **Procedures and Activities.** Should you choose to participate, you will be an intervention site participant. You will be asked to complete brief surveys at enrollment, at delivery, and at approximately 6 months post-partum. You will be enrolled in the HITSystem intervention to support your PMTCT services through 6 months postpartum. This will include getting SMS to support ART adherence, to remind you of upcoming appointments, to prepare you for a hospital delivery, and to support your infant’s linkage to early HIV testing. In addition to scheduled viral load tests, you will be asked to have one additional viral load test within 4 weeks of delivery. The results of this VL test will be used only for research purposes and will not be shared with your clinical team. If this VL test at delivery or other guideline recommended VL tests are missed, study and clinic staff may reach out in various ways – including home visits - to try to coordinate the test. These visits will be introduced as well-baby visits to protect your confidentiality, and VL will only be collected once privacy is confirmed and consent provided. Visits will be rescheduled if your privacy is perceived to be compromised at any time. After you complete the study, your clinical data will be analysed together with other participants’ data. - **Risks.** The potential risks of your participation are unintentional disclosure of your HIV status, and on rare occasions some participants may feel distressed. We have taken several precautions to make sure this will not happen. Your information will not be linked to your name or any other identifying information. All individual data is handled only by study members or clinical staff trained in human subject research to ensure confidentiality and respect of persons is maintained at all times. The study coordinator is trained in basic counselling techniques to assist participants who may feel distressed. If necessary, a community health worker will be sent to follow-up. - **Benefits**. If you enroll in this study, it means that you will benefit from more frequent communication and support for the multiple steps in the PMTCT process. This can improve the chances of having a HIV-free infant. - **Alternatives.** Instead of participating, you could either (1) receive standard PMTCT services at the hospital, without HITSystem support, or (2) receive HITSystem-supported PMTCT services without your data being included in study analyses. |

**Purpose of the Research:** Briefly describe the purpose of the study.

1. **Description of the Research:**

This hospital has been randomly selected to be an intervention site. That means that if you choose to participate, you will be an intervention site participant. You will be asked to complete brief surveys at enrollment, at delivery, and at approximately 6 months post-partum. These surveys will take approximately 30 minutes each and will be conducted by hospital or clinic staff in a private room. You may choose not to answer any questions or withdraw at any time.

You will also receive HITSystem-supported PMTCT services through 6 months postpartum. You will be offered all recommended PMTCT services. The HITSystem is an intervention that aims to improve mother-baby retention in PMTCT services. The HITSystem will send you SMS to support ART adherence, to remind you of upcoming appointments, to prepare you for a hospital delivery, and to support your infant’s linkage to early HIV testing. It will also alert your clinical provider if you have missed a PMTCT service – such as an appointment or a viral load test – so that s/he can more easily follow up with you.

In addition to scheduled viral load tests per Kenya’s national guidelines, you will be asked to have one additional viral load test within 4 weeks of delivery. The results of this VL test will be used only for research purposes and will not be shared with your clinical team. If this VL test at delivery or other guideline recommended VL tests are missed, study and clinic staff may reach out in various ways – including home visits - to try to coordinate the test. These visits will be introduced as well-baby visits to protect your confidentiality, and VL will only be collected once privacy is confirmed and consent provided. Visits will be rescheduled if your privacy is perceived to be compromised at any time.

Data collection for the study will continue until 6 months postpartum (i.e. until your baby is 6 months old). At that point, you will have the option to continue utilizing the HITSystem to help track your infant’s early infant diagnosis (EID) service; however, should you choose to continue using the HITSystem, this data will not be used for the study.

If any changes are made to the study or should new information become available, you be so informed.

1. **Human genome sequencing**

Not applicable

1. **Storage of specimen, exportation of samples and further studies:**

Per the hospital’s typical method of viral load sample processing, all viral load samples obtained as part of this study will be collected as dried blood spots (DBS) and sent directly to the laboratory for processing. All samples will be destroyed after processing. Samples will not be used for future research.

1. **Potential Harm, Injuries, Discomforts or Inconvenience, Risks:**

While we believe it to be minimal, participation in this study does hold potential risk. As with any study dealing with HIV, loss of confidentiality and stigma are concerns. While extensive measures will be taken to prevent unintentional disclosure, this could result in participants being treated unfairly or discriminated against, or could face problems being accepted by their partner, family or community. The risk of psychological and emotional distress exists, particularly for mothers of infants receiving HIV tests (distress caused by seeing their young infants bled) and to women newly informed of their infant’s HIV diagnosis. There is also a risk that participants will become upset while discussing their own or their infants’ HIV diagnosis. Mothers receiving viral load testing may experience minimal pain and discomfort while providing blood samples.

1. **Potential Benefits:**

This study also has the potential to benefit you directly. HITSystem-supported PMTCT has the potential to help mothers and infants stay retained in care. Complete PMTCT and guideline-adherence VL monitoring and clinical management has the potential to improve health and prevent transmission of HIV to their infants. If transmission does occur, earlier linkage to infant HIV testing can allow for earlier treatment initiation, which has been shown to improve outcomes in HIV-infected infants. Furthermore, findings from this study will make an important contribution to PMTCT services in Kenya. This data will improve strategies to meet the global goal of eliminating new HIV infections in children through PMTCT services.

1. **Alternative Procedures or Treatments:**

You are free to either participate or not to participate in this study. If you decide you do not want to participate in this part of the study, this will have no effect on the quality of your care and you will receive PMTCT and EID services that are standard to this facility. You also have a right at any time to change your mind not to participate in the study even after accepting to participate.

1. **Confidentiality:**

Your confidentiality is of utmost importance to us and we have taken several steps to ensure privacy is maintained throughout the study. Extensive measures have been taken in the development of the HITSystem to customize text messages to avoid risk of disclosing the caregivers’ or their infants’ HIV status or increased stigma; messages sent by the HITSystem never indicate that the hospital visit is related in any way to HIV. Access to HITSystem and other study data will be restricted only to research staff directly involved in implementation and evaluation of the study.

After you complete the study, your clinical data will be analysed together with other participants’ data. Data may be shared with study sponsors, a safety and monitoring board, other researchers, and/or be deposited in public databases to adhere to current data access practices. If data is shared, study staff will protect your personal information closely so no one will be able to connect your responses or data with any information that identifies who you are (i.e name, birthdate, etc). Your identifying information will be replaced with a coded study ID number, and only study and clinical staff will have access to the information that links your study ID with your identifying information.

1. **Reimbursement:**

Participants will receive 200 Kenyan shillings ($2 USD) at completion of each survey (enrollment, delivery, postpartum). In addition, 200 Kenyan shillings ($2 USD) will be provided for the delivery viral load test, since this is not a per-guideline test. Lastly, 200 Kenya shillings ($2 USD) will be provided for postpartum VL tests conducted outside the hospital (home visits). In total, participants may receive up to 1000 Kenyan shillings ($10 USD).

1. **Participation:**

If you choose to participate in the study, you may choose which types of text messages you would like to receive from the HITSystem: ART adherence support, appointment reminders, and/or hospital delivery support. You may also choose the frequency in which you receive ART adherence support text messages (daily, weekly, monthly, never).

You have a right at any time to change your mind not to participate in the study even after accepting to participate.

1. **Sponsorship and Early Termination:**

Every year, a review board will review study data to ensure participant safety. If this board deems that participant safety is compromised, the study may be stopped early. Furthermore, when the study is 2/3 complete, an independent group of researchers will analyze early study findings. If the benefits of this early analysis are clear, they may recommend to stop the study early. Should the study sponsor terminate funding prematurely, the study may also be stopped early. If the study needs to stop early for any reason, study team members will work with clinical staff to support all participants to receive complete, uninterrupted PMTCT care**.**

1. **Contact:**

a. For any questions or concerns about a study or in the event of a study-related injury, please contact the principal investigator, May Maloba at 0720254069.

For any questions pertaining to rights as a research participant, the contact person is: The Committee Chairperson, KEMRI Scientific and Ethics Review Unit, P. O. Box 54840-00200, Nairobi; Telephone numbers: 020-2722541, 0717719477; Email address: seru@kemri.org

1. **Consent and signature options:** Please have the participant initial or thumbprint A SINGLE STATEMENT in each of the following sections:
2. **Current Study Consent**

___________ I agree to participate in the current study

___________ I do not agree to participate in the current study

1. **Data Sharing**

___________ I agree for my information to be shared with other researchers without my additional consent only if identifiers have been removed

___________ I do not agree for my information to be shared with other researchers without my additional consent with/without identifiers

1. **Future research**

_______I agree for my information to be used for future research and shared with other researchers without

my additional consent as long as identifiers have been removed

_______I do not agree for my specimen/information to be used for future research or shared with other researchers with

or without identifiers

**SIGNATURE OF PARTICIPANT**

________________________________________

Printed Name of Participant

________________________________________ ______________

Signature of Participant Date

**_______________________________________________________________­­­­­­­­­­­­­­­­­­**

**Permanent Address of Participant**

***(****Use the following signature blocks for representative, parents, and guardians, only if applicable****)***

Your signature below indicates you are legally authorized to act on behalf of the participant, and have read this document. You will receive a copy of this document. *(The Principal Investigator is responsible for confirming that an individual is a Legally Authorized Representative based on local and state laws.)*

**SIGNATURE OF LEGALLY AUTHORIZED REPRESENTATIVE**

_______________________________________

Printed Name of Legally Authorized Representative

_______________________________________

Relationship to the Participant

________________________________________ ______________

Signature of Legally Authorized Representative Date

*(Remove the witness signature if this study is conducted under ICH GCP. Determine if your institution requires witness to the entire consent process or only witness to the final signature.)*

**SIGNATURE OF WITNESS TO CONSENT/CONSENT PROCESS**

(This individual can be a relative of the participant, but cannot be an individual involved with the research study.)

_______________________________________

Printed Name of Witness

________________________________________ ______________

Signature of Witness Date

**SIGNATURE OF INDIVIDUAL ADMINISTERING CONSENT**

(Can only be signed by an investigator or staff approved to administer consent)

_______________________________________

Printed Name of Administering Individual

________________________________________ ______________

Signature of Administering Individual Date

**INFORMED CONSENT DOCUMENT: CONTROL SITE**

**Title of the Research Study:** Evaluating the HITSystem to improve PMTCT retention and maternal viral suppression in Kenya

**Investigator(s) – Local and International Collaborators:**

**Primary Investigators:** May Maloba MCH; Sharon Mokua, MSc; Dr. Sarah Finocchario Kessler, PhD, MPH

**Co-Investigators:** Natabhona Mabachi, PhD; Brad Gautney, MPH; Kathy Goggin, PhD; Vincent Staggs, PhD

**Study location:** Kenya

You are being asked to take part in a research study. The box below tells you important things you should think about before deciding to join the study. We will provide more detailed information below the box. Please ask questions about any of the information before you decide whether to participate. You may also wish to talk to others (for example, your family, friends, or your doctor) about this study, before agreeing to join.

| **Key Information for You to Consider(Not more than 500 words)** |
| --- |
| - **Voluntary Consent**. You are being asked to volunteer for a research study. It is up to you whether you choose to participate or not. There are no penalties and you will not lose anything if you decide not to join or if after you join, you decide to quit. - **Purpose**. The HITSystem is an intervention that aims to improve PMTCT and EID by sending SMS reminders to women and their providers. This study aims to evaluate the HITSystem’s ability to improve PMTCT care and viral suppression among pregnant and postpartum women. - **Duration.** Your participation in the study will last until approximately 6 months after your baby is born. - **Procedures and Activities.** Should you choose to participate, you will be a control site participant. You will be asked to complete brief surveys at enrollment, at delivery, and at approximately 6 months post-partum. You will be enrolled in this facility’s standard PMTCT services through 6 months postpartum. In addition to scheduled viral load tests, you will be asked to have one additional viral load test within 4 weeks of delivery. The results of this VL test will be used only for research purposes and will not be shared with your clinical team. If this VL test at delivery or other guideline recommended VL tests are missed, study and clinic staff may reach out in various ways – including home visits - to try to coordinate the test. These visits will be introduced as well-baby visits to protect your confidentiality, and VL will only be collected once privacy is confirmed and consent provided. Visits will be rescheduled if your privacy is perceived to be compromised at any time. After you complete the study, your clinical data will be analysed together with other participants’ data. - **Risks.** The potential risks of your participation are unintentional disclosure of your HIV status, and on rare occasions some participants may feel distressed. We have taken several precautions to make sure this will not happen. Your information will not be linked to your name or any other identifying information. All individual data is handled only by study members or clinical staff trained in human subject research to ensure confidentiality and respect of persons is maintained at all times. The study coordinator is trained in basic counselling techniques to assist participants who may feel distressed. If necessary, a community health worker will be sent to follow-up. - **Benefits**. There are no direct benefits to study participation. - **Alternatives.** Instead of participating, you could receive standard PMTCT services at the hospital, without your data being included in study analyses. |

**Purpose of the Research:** Briefly describe the purpose of the study.

1. **Description of the Research:**

This hospital has been randomly selected to be an control site. That means that if you choose to participate, you will be an control site participant. You will be asked to complete brief surveys at enrollment, at delivery, and at approximately 6 months post-partum. These surveys will take approximately 30 minutes each and will be conducted by hospital or clinic staff in a private room. You may choose not to answer any questions or withdraw at any time.

You will receive standard PMTCT services through 6 months postpartum. You will be offered all recommended PMTCT services. In addition to being offered scheduled viral load tests, per Kenya’s national guidelines, you will be asked to have one additional viral load test within 4 weeks of delivery. The results of this VL test will be used only for research purposes and will not be shared with your clinical team. If this VL test at delivery or other guideline recommended VL tests are missed, study and clinic staff may reach out in various ways – including home visits - to try to coordinate the test. These visits will be introduced as well-baby visits to protect your confidentiality, and VL will only be collected once privacy is confirmed and consent provided. Visits will be rescheduled if your privacy is perceived to be compromised at any time.

Data collection for the study will continue until 6 months postpartum (i.e. until your baby is 6 months old). If any changes are made to the study or should new information become available, you be so informed.

1. **Human genome sequencing**

Not applicable

1. **Storage of specimen, exportation of samples and further studies:**

Per the hospital’s typical method of viral load sample processing, all viral load samples obtained as part of this study will be collected as dried blood spots (DBS) and sent directly to the laboratory for processing. All samples will be destroyed after processing. Samples will not be used for future research.

1. **Potential Harm, Injuries, Discomforts or Inconvenience, Risks:**

While we believe it to be minimal, participation in this study does hold potential risk. As with any study dealing with HIV, loss of confidentiality and stigma are concerns. While extensive measures will be taken to prevent unintentional disclosure, this could result in participants being treated unfairly or discriminated against, or could face problems being accepted by their partner, family or community. The risk of psychological and emotional distress exists, particularly for mothers of infants receiving HIV tests (distress caused by seeing their young infants bled) and to women newly informed of their or their infant’s HIV positive diagnosis. There is also a risk that participants will become upset while discussing their or their infants’ HIV diagnosis. Mothers receiving viral load testing may experience minimal pain and discomfort while providing blood samples.

1. **Potential Benefits:**

There is no direct benefits to study participation. However, findings from this study will make an important contribution to PMTCT services in Kenya. This data will improve strategies to meet the global goal of eliminating new HIV infections in children through PMTCT services.

1. **Alternative Procedures or Treatments:**

You are free to either participate or not to participate in this study. If you decide you do not want to participate in this part of the study, this will have no effect on the quality of your care and you will receive PMTCT and EID services that are standard to this facility. You also have a right at any time to change your mind not to participate in the study even after accepting to participate.

1. **Confidentiality:**

Your confidentiality is of utmost importance to us and we have taken several steps to ensure privacy is maintained throughout the study. Access to study data will be restricted only to research staff directly involved in implementation and evaluation of the study. After you complete the study, your clinical data will be analyzed together with other participants’ data. Data may be shared with study sponsors, a safety and monitoring board, other researchers, and/or be deposited in public databases to adhere to current data access practices. If data is shared, study staff will protect your personal information closely so no one will be able to connect your responses or data with any information that identifies who you are (i.e name, birthdate, etc). Your identifying information will be replaced with a coded study ID number, and only study and clinical staff will have access to the information that links your study ID with your identifying information.

1. **Reimbursement:**

Participants will receive 200 Kenyan shillings ($2 USD) at completion of each survey (enrollment, delivery, postpartum). In addition, 200 Kenyan shillings ($2 USD) will be provided for the delivery viral load test, since this is not a per-guideline test. Lastly, 200 Kenya shillings ($2 USD) will be provided for postpartum VL tests conducted outside the hospital (home visits). In total, participants may receive up to 1000 Kenyan shillings ($10 USD).

1. **Participation:**

You have a right at any time to change your mind not to participate in the study even after accepting to participate.

1. **Sponsorship and Early Termination:**

Every year, a review board will review study data to ensure participant safety. If this board deems that participant safety is compromised, the study may be stopped early. Furthermore, when the study is 2/3 complete, an independent group of researchers will analyze early study findings. If the benefits of this early analysis are clear, they may recommend to stop the study early. Should the study sponsor terminate funding prematurely, the study may also be stopped early. If the study needs to stop early for any reason, study team members will work with clinical staff to ensure all participants receive complete, uninterrupted PMTCT care**.**

1. **Contact:**

a. For any questions or concerns about a study or in the event of a study-related injury, please contact the principal investigator, May Maloba at 0720254069.

For any questions pertaining to rights as a research participant, the contact person is: The Committee Chairperson, KEMRI Scientific and Ethics Review Unit, P. O. Box 54840-00200, Nairobi; Telephone numbers: 020-2722541, 0717719477; Email address: seru@kemri.org

*(Signature blocks for participant and individuals administering consent must be part of all forms. Other signature blocks will be included when appropriate, as when the research study involves children, surrogate consent etc.)*

1. **Consent and signature options:** (Write a statement of consent and give signature options and a thumbprint for illiterate study participants. Thumbprint requires a witness of an independent person outside the study team.
2. **Current Study Consent**

___________ I agree to participate in the current study

___________ I do not agree to participate in the current study

1. **Data Sharing**

___________ I agree for my information to be shared with other researchers without my additional consent only if identifiers have been removed

___________ I do not agree for my information to be shared with other researchers without my additional consent with/without identifiers

1. **Future research**

_______I agree for my information to be used for future research and shared with other researchers without

my additional consent as long as identifiers have been removed

_______I do not agree for my specimen/information to be used for future research or shared with other researchers with

or without identifiers

**SIGNATURE OF PARTICIPANT**

________________________________________

Printed Name of Participant

________________________________________ ______________

Signature of Participant Date

**_______________________________________________________________­­­­­­­­­­­­­­­­­­**

**Permanent Address of Participant**

***(****Use the following signature blocks for representative, parents, and guardians, only if applicable****)***

Your signature below indicates you are legally authorized to act on behalf of the participant, and have read this document. You will receive a copy of this document. *(The Principal Investigator is responsible for confirming that an individual is a Legally Authorized Representative based on local and state laws.)*

**SIGNATURE OF LEGALLY AUTHORIZED REPRESENTATIVE**

_______________________________________

Printed Name of Legally Authorized Representative

_______________________________________

Relationship to the Participant

________________________________________ ______________

Signature of Legally Authorized Representative Date

*(Remove the witness signature if this study is conducted under ICH GCP. Determine if your institution requires witness to the entire consent process or only witness to the final signature.)*

**SIGNATURE OF WITNESS TO CONSENT/CONSENT PROCESS**

(This individual can be a relative of the participant, but cannot be an individual involved with the research study.)

_______________________________________

Printed Name of Witness

________________________________________ ______________

Signature of Witness Date

**SIGNATURE OF INDIVIDUAL ADMINISTERING CONSENT**

(Can only be signed by an investigator or staff approved to administer consent)

_

______________________________________

Printed Name of Administering Individual

________________________________________ ______________

Signature of Administering Individual Date

**INFORMED CONSENT DOCUMENT: PROVIDER SURVEY**

**Title of the Research Study:** Evaluating the HITSystem to improve PMTCT retention and maternal viral suppression in Kenya

**Investigator(s) – Local and International Collaborators:**

**Primary Investigators:** May Maloba MCH; Sharon Mokua, MSc; Dr. Sarah Finocchario Kessler, PhD, MPH

**Co-Investigators:** Natabhona Mabachi, PhD; Brad Gautney, MPH; Kathy Goggin, PhD; Vincent Staggs, PhD

**Study location:** Kenya

You are being asked to take part in a research study. The box below tells you important things you should think about before deciding to join the study. We will provide more detailed information below the box. Please ask questions about any of the information before you decide whether to participate. You may also wish to talk to others (for example, your family, friends, or your doctor) about this study, before agreeing to join.

| **Key Information for You to Consider(Not more than 500 words)** |
| --- |
| - **Voluntary Consent**. You are being asked to volunteer for a research study. It is up to you whether you choose to participate or not. There are no penalties and you will not lose anything if you decide not to join or if after you join, you decide to quit. - **Purpose**. The HITSystem is an intervention that aims to improve PMTCT and EID by sending SMS reminders to women and their providers. This study aims to evaluate the HITSystem’s ability to improve PMTCT care and viral suppression among pregnant and postpartum women. As part of this, we are assessing provider role, department, level of experience, knowledge and motivation regarding current PMTCT guidelines, and barriers and facilitators to provision of PMTCT services. - **Duration.** We will ask you to complete surveys at two time points: at enrollment and at the end of the study (in approximately 4 years). - **Procedures and Activities.** Should you choose to participate, you will take a survey now and at the end of the study. Each survey is expected to take approximately 30 minutes and will be conducted by study staff in a private room at the hospital. - **Risks.** While we have taken multiple steps to protect your privacy, the potential risk of loss of confidentiality exists. - **Benefits**. There are no direct benefits to study participation. - **Alternatives.** There are no penalties for not participating in the study. |

**Purpose of the Research:** Briefly describe the purpose of the study.

1. **Description of the Research:**

If you choose to participate in the study, you will complete a survey at enrollment (today) and at the end of the study period (in approximately 4 years). Study questions focus on your role at the hospital, knowledge of PMTCT and viral load guidelines, and motivation and self-efficacy for following guidelines. Surveys will be administered by study staff in a private room and are expected to take approximately half an hour.

1. **Human genome sequencing**

Not applicable

1. **Storage of specimen, exportation of samples and further studies:**

Not applicable, no specimens will be collected as part of your participation in this research.

1. **Potential Harm, Injuries, Discomforts or Inconvenience, Risks:**

While we believe it to be minimal, participation in this study does hold potential risk. As with any study, the potential risk of loss of confidentiality exists. Providers will be discussing the standard of care and guidelines at their facility. If confidentiality is breached, there is a concern that negative statements may get back to superiors and result in disciplinary action or other repercussions.

1. **Potential Benefits:**

There are no direct benefits to study participation. However, findings from this study will make an important contribution to PMTCT services in Kenya. This data will improve strategies to meet the global goal of eliminating new HIV infections in children through PMTCT services.

1. **Alternative Procedures or Treatments:**

You are free to either participate or not to participate in this study. If you decide you do not want to participate in the study, this will have no impact on your role at the hospital. You also have a right at any time to change your mind not to participate in the study even after accepting to participate.

1. **Confidentiality:**

Your confidentiality is of utmost importance to us and we have taken several steps to ensure privacy is maintained throughout the study. Access to study data will be restricted only to research staff directly involved in implementation and evaluation of the study. After you complete the study, your data will be analysed together with other participants’ data. Data may be shared with study sponsors, a safety and monitoring board, other researchers, and/or be deposited in public databases to adhere to current data access practices. If data is shared, study staff will protect your personal information closely so no one will be able to connect your responses or data with any information that identifies who you are (i.e name, birthdate, role, etc.). Your identifying information will be replaced with a coded study ID number, and only study and clinical staff will have access to the information that links your study ID with your identifying information.

1. **Reimbursement:**

Participants will receive 500 Kenyan shillings ($5 USD) at completion of each survey (enrollment, end of study).

1. **Participation:**

You have a right at any time to change your mind not to participate in the study even after accepting to participate.

1. **Sponsorship and Early Termination:**

Every year, a review board will review study data to ensure participant safety. If this board deems that participant safety is compromised, the study may be stopped early. Furthermore, when the study is 2/3 complete, an independent group of researchers will analyze early study findings. If the benefits of this early analysis are clear, they may recommend to stop the study early. Should the study sponsor terminate funding prematurely, the study may also be stopped early. If the study needs to stop early for any reason, study team members will conduct the end-of-study provider surveys immediately after study ends.

1. **Contact:**

a. For any questions or concerns about a study or in the event of a study-related injury, please contact the principal investigator, May Maloba at 0720254069.

For any questions pertaining to rights as a research participant, the contact person is: The Committee Chairperson, KEMRI Scientific and Ethics Review Unit, P. O. Box 54840-00200, Nairobi; Telephone numbers: 020-2722541, 0717719477; Email address: seru@kemri.org

*(Signature blocks for participant and individuals administering consent must be part of all forms. Other signature blocks will be included when appropriate, as when the research study involves children, surrogate consent etc.)*

1. **Consent and signature options:** (Write a statement of consent and give signature options and a thumbprint for illiterate study participants. Thumbprint requires a witness of an independent person outside the study team
2. **Current Study Consent**

___________ I agree to participate in the current study

___________ I do not agree to participate in the current study

1. **Data Sharing**

___________ I agree for my information to be shared with other researchers without my additional consent only if identifiers have been removed

___________ I do not agree for my information to be shared with other researchers without my additional consent with/without identifiers

1. **Future research**

_______I agree for my information to be used for future research and shared with other researchers without

my additional consent as long as identifiers have been removed

_______I do not agree for my specimen/information to be used for future research or shared with other researchers with

or without identifiers

**SIGNATURE OF PARTICIPANT**

________________________________________

Printed Name of Participant

________________________________________ ______________

Signature of Participant Date

**_______________________________________________________________­­­­­­­­­­­­­­­­­­**

**Permenent Address of Participant**

***(****Use the following signature blocks for representative, parents, and guardians, only if applicable****)***

Your signature below indicates you are legally authorized to act on behalf of the participant, and have read this document. You will receive a copy of this document. *(The Principal Investigator is responsible for confirming that an individual is a Legally Authorized Representative based on local and state laws.)*

**SIGNATURE OF LEGALLY AUTHORIZED REPRESENTATIVE**

_______________________________________

Printed Name of Legally Authorized Representative

_______________________________________

Relationship to the Participant

________________________________________ ______________

Signature of Legally Authorized Representative Date

*(Remove the witness signature if this study is conducted under ICH GCP. Determine if your institution requires witness to the entire consent process or only witness to the final signature.)*

**SIGNATURE OF WITNESS TO CONSENT/CONSENT PROCESS**

(This individual can be a relative of the participant, but cannot be an individual involved with the research study.)

_______________________________________

Printed Name of Witness

________________________________________ ______________

Signature of Witness Date

**SIGNATURE OF INDIVIDUAL ADMINISTERING CONSENT**

(Can only be signed by an investigator or staff approved to administer consent)

_______________________________________

Printed Name of Administering Individual

________________________________________ ______________

Signature of Administering Individual Date

**KIAMBATISHO F: FOMU YA IDHINI KWA MSHIRIKI**

**JINA LA UTAFITI:** UTATHMINI WA MFUMO WA HITSYSTEM ILI KUBORESHA UZINGATIVU WA HUDUMA ZA KUMKINGA MTOTO DHIDI YA MAAMBUKIZO TOKA KWA MAMA (PMTCT), NA UKANDAMIZAJI WA KIWANGO CHA VIRUSI KWA AKINA MAMA NCHINI KENYA

**Watafiti – Washiriki wakuu Nchini, na wamataifa:**

**Watafiti Wakuu:** May Maloba MCH; Sharon Mokua, MSc; Dr. Sarah Finocchario Kessler, PhD, MPH

**Watafiti Wenza:** Natabhona Mabachi, PhD; Brad Gautney, MPH; Kathy Goggin, PhD; Vincent Staggs, PhD

**Eneo la Utafiti:** Kenya

Unaulizwa kushiriki katika utafiti. Sanduku hapo chini linamaelezo ya mambo muhimu unayopaswa kuyatafakari, kabla ya kufanya uamuzi wa kujiunga na utafiti. Tutakupa maelezo zaidi hapo chini, baada ya kisanduku. Tafadhali uliza maswali kuhusu maelezo yoyote, kabla ya kuamua iwapo utashiriki. Pia unaweza kuongea na watu wengine (K.V: familia yako, marafiki au daktari wako) kuhusu utafiti huu, kabla ya kuamua kujiunga.

| **Habari Muhimu Kwako Kuzingatia (Isizidi maneno 500)** |
| --- |
| - **Idhini ya Hiari.** Unaulizwa ujitolee kwa utafiti. Ni hiarii yako ikiwa utachagua kushiriki au la. Hakuna adhabu yoyote na hautapoteza chochote ukiamua kutojiunga au ikiwa baada ya kujiunga utaamua kujitoa kwenye utafiti. - **Lengo.** HITSystem ni mfumo unao lenga kuboresha huduma za PMTCT na EID, kwa kutuma vikumbusho vya SMS kwa akina mama, na wahudumu wao. Utafiti huu unalenga kutathmini uwezo wa HITSystem kuboresha taratibu za PMTCT, na za ukandamiza wa viwango vya virusi kwa akina mama waja wazito, na walio jifungua. - **Muda.** Ushiriki wako kwenye utafiti, utadumu hadi takriban miezi 6 baada ya kujifungua. - **Taratibu na Shughuli.** Endapo utachagua kushiriki, utakuwa mshiriki kwenye utaratibu.Utaulizwa kukamilisha maswali kwa kifupi: wakati wa usajili, wakati wa kujifungua na takriban miezi 6 baada ya kujifungua. Utasajiliwa kwenye mfumo wa HITSystem ili kukusaidia katika taratibu zako PMTCT, na hadi miezi 6 baada ya kujifungua. Hii itajumuisha wewe kupata vikumbusho vya SMS, ili kukusaidia kwa ufautiliaji wako wa umeji dawa za kukabiliana na virusi ipasavyo, kukukumbusha kuhusu tarehe zijazo za kliniki, kukutayarisha kuhusu kujifungulia hospitali, na kusaidia kwa kumuingisha mwanao kwenye utaratibu wa ugunduzi virusi mapema. Mbali na mpangilio wa uchunguzi wa kiwango cha virusi, utaulizwa upimwe kipimo kingine tena cha kuchunguza kiwango cha virusi, ndani ya wiki nne baada ya kujifungua. Matokeo ya uchunguzi huu, yatatumika tu, kwa taratibu za utafiti, na hayata kabithiwa wahudumu wako wako kiliniki. Endapo kipimo hiki cha kiwango cha virusi baada ya kujifungua, au kipimo kingine kulingana na mwongozo uliopendekezwa, hakikutolea, wahuduma wa kliniki au wa utafiti watajaribu kukufikia kwa njia tofauti ikiwemo: kukutembelea nyumbani – ili kujaribu kufanya upimaji. Ziara hizi zitatajwa kua ziara za kutembelea watoto wachanga ili kudumisha usiri, na sampuli ya kipimo cha kiwango cha virusi itatolewa tu, baada ya hakikisho la usiri, na idhini kupeanwa. Ziara ita hairishwa endapo faragha yako itabainika kuto kuwa na usiri. Data yako ya kliniki itachambuliwa pamoja na data za washiriki wengine, baada ya kukamilika kwa utafiti. - **Hathari.** Hathari zinazo weza kutokea kwa kushiriki kwako ni kufichuka kwa hali yako ya VVU bila kukusudia, na mara chache, washirik wengine wanaweza kuhisi kufadhaika. Tumechukua tahadhari kadhaa ili kuhakikisha kwamba haya hayatokei. Taarifa zako hazitaambatanishwa na jina lako au habari nyingine yoyote itakayoweza kukutambulisha. Maelezo yote ya kibinafsi hufikiwa tu, na washiriki wa utafiti au wahuduma wa kliniki waliopewa mafunzo maalum ya utafiti unaohusu binadamu ili kuhakikisha usiri na heshima ya watu ina dumishwa wakati wote. Mratibu wa utafiti amepata mafunzo ya ushauri nasaha ili, kusaidia washiriki watakao hisi fedheha. Ku kiwa na haja, mhudumu wa afya ya jamii atatumwa kufuatilia. - **Manufaa**. Ukiamua kushiriki katika utafiti huu, itamaanisha kwamba, utanufaika na mawasiliano ya mara kwa mara na usaidizi katika taratibu kadhaa wa kadha, za PMTCT. Hii inaweza kuboresha nafasi ya kuwa na mtoto asiye na maambukizo ya VVU. - **Mambo m’badala.** Hata usipo shiriki, bado utaweza 1) Kupokea huduma za kawaida za PMTCT hospitalini bila mfumo wa HITSystem, au 2) Kupokea huduma za PMTCT zinazo tekelezwa kupitia mfumo wa HITSystem, pasi na kuhusisha taarifa zako kwenye uchambuzi wa utafiti huu. |

**Lengo la utafiti:** Maelezo kwa kifupi.

1. **Maelezo ya Utafiti:**

Hosptali hii imechaguliwa kwa nasibu, kutumia mfumo. Hii inamaanisha kwamba, ukichagua kushiriki, utakuwa mshiriki katika utaratibu wa kutumia mfumo. Utaulizwa kukamilisha maswali mafupi wakati wa usajili, utakapo jifungua, na takriban miezi 6 baada ya kujifungua. Maswali yata kutachukua takriban dakika 30 na yataulizwa na wahudumu wa hospitali au kliniki, kwenye chumba binafsi. Unaweza kuchagua kutojibu maswali yoyote au kujiondoa wakati wowote.

Utapokea huduma za PMTCT zinazo tumia mfumo wa HITSystem – hadi miezi 6 baada ya kujifungua. Utapokea huduma zote zilizopendekezwa za PMTCT. HITSystem ni mfumo unao lenga kuboresha taratibu za uzingativu wa mama na mtoto katika taratibu za PMTCT. Mfumo wa HITS utakutumia SMS ili kukusaidia kwa uzingativu wa umezaji dawa za ART ipasavyo, kukukumbusha tarehe zako zijazo za kliniki, kukuandaaa kwa kujifungua hospitalini, na kusaidia kwa kumuunganisha mwanao na taratibu za ugunduzi wa VVU kwa watoto wachanga mapema. Pia itampa tahadhari mhudumu wako wa kliniki, endapo umekosa huduma ya PMTCT – kama vile kukosa kliniki, au Kipimo cha uchunguzi wa kiwango cha virusi – ili aweze kukufuatilia kwa urahisi.

Kuaongezea na mpangilio wa upimaji kiwango cha virusi mwilini kulingana na miongozo ya taifa la Kenya, utaulizwa upimwe kipimo kimoja zaidi, wiki nne baada ya kujifungua. Matokeo ya kipimo hiki cha kiwango cha Virusi mwilini yatatumika tu kwa taratibu za utafiti, na hayatakabidhiwa wahudumu wako. Endapo kipimo hiki cha kiwango cha virusi baada ya kujifungua, au kipimo kingine kulingana na mwongozo uliopendekezwa, hakikutolea, wahuduma wa kliniki au wa utafiti watajaribu kukufikia kwa njia tofauti ikiwemo: kukutembelea nyumbani, ili kujaribu kufanya upimaji. Ziara hizi zitatajwa kua ziara za kutembelea watoto wachanga ili kudumisha usiri, na sampuli ya kipimo cha kiwango cha virusi itatolewa tu, baada ya hakikisho la usiri, na idhini kupeanwa. Ziara ita hairishwa endapo faragha yako itabainika kuto kuwa na usiri.

Mkusanyiko wa taarifa (data) katika utafiti, utaendelea hadi miezi 6 baada ya kujifungua (Hadi mtoto wako atakapofikisha miezi 6). Hapo ndio, utapewa fursa ya kuchagua iwapo utaendelea kutumia mfumo wa HITSystem kukusaidia kufuatilia taratibu za utambuzi wa watoto wachanga mapema ( EID) – Hata endapo utatachagua kuendelea kutumia mfumo wa HITSystem, Mkusanyiko wa taarifa hii (data) hautatumiwa kwa taratibu ya utafiti.

Uta fahamishwa iwapo kutakuwa na mabadiliko yoyote kuhusu utafiti, au kukiwa na habari zozote mpya.

1. **Mpangilio wa genome la wanadamu (Human Genome Sequencing)**

Haita fanyika.

1. **Uhifadhi na Usafirishaji wa sampuli, Na utafiti zaidi:**

Kulingana na taratibu za kawaida za hospitali kwa uandaaji sampuli za kuchunguza kiwango cha virusi mwilini, sampuli zote zitakazo kusanywa kwa taratibu za utafiti huu, zitakusanywa kama damu iliyokaushawa (DBS – dried blood spot) na kutumwa moja kwa moja hadi maabara kuu ili kufanyiwa uchunguzi. Sampuli zote zitaharibiwa baada ya uchunguzi. Sampuli hazitatumika kwa utafiti wa baadaye.

1. **Uwezekano wa kuumia, majeraha, Usumbufu, Hatari:**

Hata ingawa tunatarajia iwe kwa uchache, kushiriki kwako kwa utafiti huu kunaweza kuwa na madhara. Kama ilivyo kwa tafiti zozote kuhusu maswala ya VVU, ukosefu wa usiri na unyanyapaa – ni sababu za kutia wasiwasi. Hata kama hatua thabiti zitachukuliwa kuzuia ufichuzi bila kukusudia, hali kama hii inaweza kusababisha washiriki kufanyiwa unyanya paa, au kubaguliwa, au kukumbwa na changamoto za kukubalika kwa wapenzi wao, familia au jamii. Athari za matatizo ya kisaikologia na kihemko iko, haswa kwa akina mama wenye watoto wachanga wanaopokea vipimo vya VVU (mihemko inayosababishwa na kuona watoto wao wachanga wakitolewa damu) na kwa wanawake wanaofahamishwa majibu ya vipimo vya VVU vya Watoto wao. Pia kuna hathari kwamba washiriki hujawa na hisia wakati wa kujadili kuhusu maambukizi yao, au ya watoto wao wachanga. Akina mama wanaopokea kipimo cha uchunguzi wa kiwango cha virusi mwilini, hupata maumivu na usumbufu kidogo wakati wa utoaji sampuli ya damu.

1. **Faida ya Kushiriki:**

Utafiti huu una uwezo wa kukufaidi moja kwa moja. Huduma za PMTCT zilizoimarishwa na mfumo wa HITS, iko na uwezo wa kusaidia Akina mama na Watoto wao wachanga, kuendeleza taratibu zao za huduma ipasavyo. Ufuatiliaji kamili wa PMTCT pamoja na upimaji wa kiwango cha virusi kulingana na mwongozo na usimamizi wa kliniki, viko na uwezo wa kuboresha afya na kuzuia maambukizi ya VVU kwa watoto wao wachanga. Maaunganisho ya taratibu za ugunduzi virusi kwa watoto wachanga mapema, kunaweza kusababisha kuanzishwa mapema kwa dawa za kudhibiti virusi, endapo mtoto amepatikana na maambukizo, swala ambalo limeonyesha kuboresha matokeo kwa watoto walioambukizwa VVU. Zaidi ya hayo, matokeo ya utafiti huu yatakua na mchango wa maana kwa taratibu za PMTCT nchini Kenya. Takwimu hizi zitaboresha mikakati ya kuafikia malengo ya ulimwengu ya kuangamiza maambukizo mapya ya VVU kwa watoto kupitia taratibu za PMTCT.

1. **Taratibu au Tiba Zinginezo:**

Uko na uhuru wa kushiriki au kutoshiriki katika utafiti huu. Ukiamua kutoshiriki katika utafiti huu, hili halitaathiri ubora wa utunzaji wako, na utapokea huduma za kawaida za PMTCT na EID zinapatikanazo katika kituo hiki. Pia uko nahaki ya kubadili msimamo wako wa kutohusika na utafiti huu wakati wowote, hata baada ya kukubali kushiriki.

1. **Usiri:**

Usiri wako ni muhimu sana kwetu na tumechukua kila hatua kuhakikisha siri yako imetunzwa wakati wote wa utafiti.

Hatua kubwa zimechukuliwa katika uundaji wa mfumo wa HITS ili kubinafsisha jumbe za maandisha (SMS) kuzuia hatari ya kufichua hali ya akina mama, ya watoto wao wachanga, au kuongezeka kwa unyanyapaa; Jumbe na taarifa kwa mfumo wa HITS hauonyeshi kamwe kama ziara ya hospitalini inahusiana kwa njia yoyote na VVU. Ni wahusika wa utafiti pekee ndio watapewa idhini kufikia jumbe na taarifa zako zihusianazo na HITS, au utafiti huu

Baada kukamilisha utafiti, data yako ya kliniki itachambuliwa pamoja na data ya washiriki wengine. Takwimu za ujumbe kukuhusu, zinaweza kuchambuliwa na wadhamini wa utafiti, bodi ya usalama na ukaguzi, watafiti wengine, na pia inaweza kuwekwa kwenye hifadhidata ya umma, kulinga na utaaratibu wa data ulioko sasa. Ikiwa data kukuhusu itashirikishwa, wafanyikazi wa utafiti watalinda maelezo binafsi kukuhusu, na hakuna mtu atakayeweza kuunganisha taarifa zako (data) na habari yoyote itakayo weza kufanya ugunduliwe (K.v. jina lako, siku ya kuzaliwa..n.k). Taarifa inayo weza kukutambulisha itabadilishwa na nambari maalum ya utafiti, na ni watafiti na wataalam wa kliniki pekee watakao weza kuunganisha taarifa binafsi, na na habari husika.

1. **Fidia/Malipo:**

Washiriki watapokea shilingi 200 (Dola 2 za Marekani) kwa kukamilisha kila uchunguzi (Wakati wa usajili, kujifungua, baaada ya kujifungua). Pia, shlingi 200 zitatopeanwa kwa kipimo cha kiwango cha virusi baada ya kujifungua, kwa sababu, kipimo hiki hakiambatani na mwongozo wa kitaifa nchini Kenya. Mwishowe, shilingi 200 zitaongezwa kwa vipimo vya virusi baada ya kujifungua, kitakacho tolewa nje ya hosptali (Wakati wa kutembelewa nyumbani). Kwa jumla, washiriki wanaweza kupokea hadi shilingi 1000 za Kenya (Dola 10 za Marekani).

1. **Ushiriki:**

Ukichagua kushiriki katika utafiti, unaweza kuchagua aina ya SMS ambayo ungetaka kupokea kutoka kwa HITSystem: ili kukusaidia kwa ufautiliaji wako wa umezaji dawa za kukabiliana na virusi ipasavyo, kukukumbusha kuhusu tarehe zijazo za kliniki au/na kujifungulia hospitalini. Pia unaweza kuchagua mpangilio ambao ungependa kupokea ujumbe wa maandishi kwa taratibu za umezaji dawa za virusi/ART (Kila siku, kila wiki, kila mwezi,kamwe).

Una haki ya kubadili msimamo wako na kutoendelea na utafiti hata baada ya kukubali kushiriki.

1. **Ufadhili na Kukomesha Mapema:**

Kila mwaka, bodi ya ukaguzi itakagua data ya utafiti ili kuhakikisha usalama wa mshiriki. Endapo bodi hii itaona usalama wa mshiriki ume atharishwa, utafiti unaweza kusimamishwa mapema. Wakati utafiti utafikia 2/3 kukamilika), kikundi huru cha watafiti, watachambua matokeo ya mapema ya utafiti. Ikiwa faida ya uchambuzi huu wa mapema inaonekana wazi, wanaweza kupendekeza kufunga utafiti mapema. Mfadhili wa utafiti akikatiza fedha mapema, utafiti utasimamishwa mapema. Ikiwa utafiti utahitaji kusimamishwa mapema kwa sababu yoyote, washiriki, watafanya kazi wa utafiti, na wahudumu wa kliniki, watashirikiana kuhakikisha kwamba washiriki wote wanapata huduma kamili, pasi kutatiza taratibu za PMTCT.

1. **Mawasiliano:**

a. Kwa maswali yoyote au wasiwasi juu ya utafiti au tukio la jeraha linalohusiana na utafiti, tafadhali wasiliana na Mpelelezi mkuu, May Maloba - 0720254069.

b. Endapo una maswali yanayohusu haki kama mshiriki wa utafiti, unaweza kuwasiliana na: Mwenyekiti wa kamati, KEMRI Scientific and Ethics Review Unit, P. O. Box 54840-00200, Nairobi; Nambari za simu: 020-2722541, 0717719477; Barua pepe: seru@kemri.org

1. **Idhini na Sahihi:** Tafadhali hakikisha mshiriki ameweka sahii/kidole kwa KILA MOJA WAPO ya sehemu zifuatazo.
2. **Dhibitisho la utafiti kwa sasa:**

___________ Ninakubali kushiriki katika utafiti huu kwa sasa

___________ Nimekataa kushiriki katika utafiti huu kwa sasa

1. **Kushiriki kwa Takwimu:**

___________ Nakubali kutumika kwa habari zangu na watafiti wengine pasipo idhini ya ziada, bora tu vitambulisho vimeondolewa.

___________ Nimekataa kutumika kwa habari zangu na watafiti wengine pasipo idhini yangu ya ziada, kukiwepo/kutokuwepo vitambulisho.

1. **Utafiti wa Baadaye**

_______Ninakubali kutumika kwa habari yangu au sampuli yangu kwa tafiti zijazo au zishirikishwe na watafiti wengine bila indini ya ziada bora tu vitambulisho vimeondolewa.

_______ Nimekataa kutumika kwa habari yangu au sampuli yangu itumike kwa utafiti ujao, au zishirikiwe na watafiti wengine bila indini yangu ya kukiwepo/ kutokuwepo vitambulisho.

**SAHIHI YA MSHIRIKI**

________________________________________

Jina la Mshiriki

________________________________________ ______________

Sahihi ya mshiriki Tarehe

**_______________________________________________________________­­­­­­­­­­­­­­­­­­**

**Anwani ya Mshiriki (Address)**

***(Sehemu ifuatayo kutumika kuweka sahihi ya msimamizi, mzazi, na Kaimu mzazi pekee)***

Sahihi yako hapo chini inaashiria kwamba uko na mamlaka kisheria kutoa uamuzi kwa niaba ya mshiriki, na umesoma jalada hili. Utapewa kopi ya jalada. *(Mtafiti mkuu ndio mwenye mamlaka ya kuthibitisha anaye dai, ako na mamlaka ya kisheria kulingana na sheria za gatuzi/taifa)*.

**SAHIHI YA MLEZI/KAIMU ANAYETAMBULIWA NA SERIKALI**

_______________________________________

Jina la mlezi/Kaimu (tumia herufi kubwa)

_______________________________________

Uhusiano na mshiriki

________________________________________ ______________

Sahihi ya mlezi/Kaimu Tarehe

**SAHIHI YA SHAHIDI**

_______________________________________

Jina la mshahidi (tumia herufi kubwa)

_______________________________________ ______________

Sahihi ya mshahidi Tarehe

**SAHIHI YA MTAFITI ANAYEPEWA IDHINI:**

______________________________________

Jina la Mtafiti

________________________________________ ______________

Sahihi ya mtafiti Tarehe

**KIAMBATANISHO F: FOMU YA IDHINI KWA MSHIRIKI**

**JINA LA UTAFITI:** UTATHMINI WA MFUMO WA HITSYSTEM ILI KUBORESHA UZINGATIVU WA HUDUMA ZA KUMKINGA MTOTO DHIDI YA MAAMBUKIZO TOKA KWA MAMA (PMTCT), NA UKANDAMIZAJI WA KIWANGO CHA VIRUSI KWA AKINA MAMA NCHINI KENYA.

**Watafiti – Washiriki wakuu Nchini, na wamataifa::**

**Watafiti Wakuu:** May Maloba MCH; Sharon Mokua, MSc; Dr. Sarah Finocchario Kessler, PhD, MPH

**Watafiti Wenza:** Natabhona Mabachi, PhD; Brad Gautney, MPH; Kathy Goggin, PhD; Vincent Staggs, PhD

**Eneo la Utafiti:** Kenya

Unaulizwa kushiriki katika utafiti. Sanduku hapo chini linamaelezo ya mambo muhimu unayopaswa kuyatafakari, kabla ya kufanya uamuzi wa kujiunga na utafiti. Tutakupa maelezo zaidi hapo chini, baada ya kisanduku. Tafadhali uliza maswali kuhusu maelezo yoyote, kabla ya kuamua iwapo utashiriki. Pia unaweza kuongea na watu wengine (K.V: familia yako, marafiki au daktari wako) kuhusu utafiti huu, kabla ya kuamua kujiunga.

| **Habari Muhimu Kwako Kuzingatia (Isizidi maneno 500)** |
| --- |
| - **Idhini ya Hiari.** Unaulizwa ujitolee kwa utafiti. Ni hiarii yako ikiwa utachagua kushiriki au la. Hakuna adhabu yoyote, na hautapoteza chochote ukiamua kutojiunga au ukiamua kujitoa kwenye utafiti, baadae. - **Lengo.** HITSystem ni mfumo unao lenga kuboresha huduma za PMTCT na EID, kwa kutuma vikumbusho vya SMS kwa akina mama, na wahudumu wao. Utafiti huu unalenga kutathmini uwezo wa HITSystem kuboresha taratibu za PMTCT, na za ukandamiza wa viwango vya virusi kwa akina mama waja wazito, na walio jifungua. - **Muda.** Ushiriki wako katika utafiti utadumu hadi takriban miezi 6 baada yam toto wako kuzaliwa. - **Taratibu na Shughuli.** Endapo utachagua kushiriki, utakuwa mshiriki kwenye utaratibu wa uthibiti.Utaulizwa kukamilisha maswali kwa kifupi: wakati wa usajili, wakati wa kujifungua na takriban miezi 6 baada ya kujifungua. Utasajiliwa kwa taratibu za kawaidi za PMTCT, na hadi miezi 6 baada ya kujifungua. Mbali na mpangilio wa kawaida wa uchunguzi wa kiwango cha virusi, utaulizwa upimwe kipimo kingine tena cha kuchunguza kiwango cha virusi, ndani ya wiki nne baada ya kujifungua. Matokeo ya uchunguzi huu, yatatumika tu, kwa taratibu za utafiti, na hayata kabithiwa wahudumu wako wako kiliniki. Endapo kipimo hiki cha kiwango cha virusi baada ya kujifungua, au kipimo kingine kulingana na mwongozo uliopendekezwa hakikutolea, wahuduma wa kliniki au wa utafiti watajaribu kukufikia kwa njia tofauti ikiwemo: kukutembelea nyumbani – ili kujaribu kufanya upimaji. Ziara hizi zitatajwa kua ziara za kutembelea watoto wachanga ili kudumisha usiri, na sampuli ya kipimo cha kiwango cha virusi itatolewa tu, baada ya hakikisho la usiri, na idhini kupeanwa. Ziara ita hairishwa endapo faragha yako itabainika kuto kuwa na usiri. Data yako ya kliniki itachambuliwa pamoja na data za washiriki wengine, baada ya kukamilika kwa utafiti. - **Hatari.** Hathari zinazo weza kutokea kwa kushiriki kwako ni kufichuka kwa hali yako ya VVU bila kukusudia, na mara chache, washirik wengine wanaweza kuhisi kufadhaika. Tumechukua tahadhari kadhaa ili kuhakikisha kwamba haya hayatokei. Taarifa zako hazitaambatanishwa na jina lako au habari nyingine yoyote itakayoweza kukutambulisha. Maelezo yote ya kibinafsi hufikiwa tu, na washiriki wa utafiti au wahuduma wa kliniki waliopewa mafunzo maalum ya utafiti unaohusu binadamu ili kuhakikisha usiri na heshima ya watu ina dumishwa wakati wote. Mratibu wa utafiti amepata mafunzo ya ushauri nasaha ili, kusaidia washiriki watakao hisi fedheha. Ku kiwa na haja, mhudumu wa afya ya jamii atatumwa kufuatilia. - **Faida.** Hakuna faida ya moja kwa moja,kwa kushiriki katika utafiti huu - **Chaguzi zingine.** **Mambo m’badala.** Hata usipo shiriki, bado utaweza kupokea huduma za kawaida za PMTCT hospitalini pasi na ujumbe/data yako kujumuishwa kwenye uchambuzi wa utafiti huu. |

**Lengo la Utafiti:** Maelezo kwa kifupi

1. **Maelezo ya Utafiti:**

Hosptali hii imechaguliwa kwa nasibu, kwa utaratibu wa kuthibiti (control site). Hii inamaanisha kwamba, ukichagua kushiriki, utakuwa mshiriki kwa utaratibu wa kuthibiti (bila mfumo). Utaulizwa kukamilisha maswali mafupi wakati wa usajili, utakapo jifungua, na takriban miezi 6 baada ya kujifungua. Maswali yata kutachukua takriban dakika 30 na yataulizwa na wahudumu wa hospitali au kliniki, kwenye chumba binafsi. Unaweza kuchagua kutojibu maswali yoyote au kujiondoa wakati wowote.

Utapokea huduma za kawaida za PMTCT hadi miezi 6 baada ya kujifungua. Utapokea huduma zote zilizopendekezwa za PMTCT. Kuaongezea na mpangilio wa upimaji kiwango cha virusi mwilini kulingana na miongozo ya taifa la Kenya, utaulizwa upimwe kipimo kimoja zaidi, wiki nne baada ya kujifungua. Matokeo ya kipimo hiki cha kiwango cha Virusi mwilini yatatumika tu kwa taratibu za utafiti, na hayatakabidhiwa wahudumu wako. Endapo kipimo hiki cha kiwango cha virusi baada ya kujifungua, au kipimo kingine kulingana na mwongozo uliopendekezwa, hakikutolea, wahuduma wa kliniki au wa utafiti watajaribu kukufikia kwa njia tofauti ikiwemo: kukutembelea nyumbani, ili kujaribu kufanya upimaji. Ziara hizi zitatajwa kuwa ziara za kutembelea watoto wachanga ili kudumisha usiri, na sampuli ya kipimo cha kiwango cha virusi itatolewa tu, baada ya hakikisho la usiri, na idhini kupeanwa. Ziara ita hairishwa endapo faragha yako itabainika kuto kuwa na usiri.

Mkusanyiko wa taarifa (data) katika utafiti, utaendelea hadi miezi 6 baada ya kujifungua (Hadi mtoto wako atakapofikisha miezi 6). Uta fahamishwa iwapo kutakuwa na mabadiliko yoyote kuhusu utafiti, au kukiwa na habari zozote mpya.

1. **Mpangilio wa genome la wanadamu (Human Genome Sequencing)**

Haita fanyika.

1. **Uhifadhi na Usafirishaji wa sampuli, Na utafiti zaidi:**

Kulingana na taratibu za kawaida za hospitali kwa uandaaji sampuli za kuchunguza kiwango cha virusi mwilini, sampuli zote zitakazo kusanywa kwa taratibu za utafiti huu, zitakusanywa kama damu iliyokaushawa (DBS – dried blood spot) na kutumwa moja kwa moja hadi maabara kuu ili kufanyiwa uchunguzi. Sampuli zote zitaharibiwa baada ya uchunguzi. Sampuli hazitatumika kwa utafiti wa baadaye.

1. **Uwezekano wa kuumia, majeraha, Usumbufu, Hatari:**

Hata ingawa tunatarajia iwe kwa uchache, kushiriki kwako kwa utafiti huu kunaweza kuwa na madhara. Kama ilivyo kwa tafiti zozote kuhusu maswala ya VVU, ukosefu wa usiri na unyanyapaa – ni sababu za kutia wasiwasi. Hata kama hatua thabiti zitachukuliwa kuzuia ufichuzi bila kukusudia, hali kama hii inaweza kusababisha washiriki kufanyiwa unyanya paa, au kubaguliwa, au kukumbwa na changamoto za kukubalika kwa wapenzi wao, familia au jamii. Athari za matatizo ya kisaikologia na kihemko iko, haswa kwa akina mama wenye watoto wachanga wanaopokea vipimo vya VVU (mihemko inayosababishwa na kuona watoto wao wachanga wakitolewa damu) na kwa wanawake wanaofahamishwa majibu ya vipimo vya VVU vya Watoto wao. Pia kuna hatari kwamba washiriki hujawa na hisia wakati wa kujadili kuhusu maambukizi yao, au ya Watoto wao wachanga. Akina mama wanaopokea kipimo cha uchunguzi wa kiwango cha virusi mwilini, hupata maumivu na usumbufu kidogo wakati wa utoaji sampuli ya damu.

1. **Faida ya Kushiriki:**

Hakuna faida kwa kushiriki katika utafiti huu. Hata hivyo, matokeo ya utafiti huu yatakua na mchango wa maana kwa taratibu za PMTCT nchini Kenya. Takwimu hizi zitaboresha mikakati ya kuafikia malengo ya ulimwengu ya kuangamiza maambukizo mapya ya VVU kwa watoto kupitia taratibu za PMTCT.

1. **Taratibu au Tiba Zingine:**

Uko na uhuru wa kushiriki au kutoshiriki katika utafiti huu. Ukiamua kutoshiriki katika utafiti huu, hili hali taathiri ubora wa utunzaji wako, na utapokea huduma za kawaida za PMTCT na EID zinapatikanazo katika kituo hiki. Pia uko nahaki ya kubadili msimamo wako wa kutohusika na utafiti huu wakati wowote, hata baada ya kukubali kushiriki

1. **Usiri:**

Usiri wako ni muhimu sana kwetu na tumechukua kila hatua kuhakikisha siri yako imetunzwa wakati wote wa utafiti.

Ni wahusika wa utafiti pekee ndio watapewa idhini kufikia jumbe na taarifa zako zihusianazo na utafiti huu

Baada kukamilisha utafiti, data yako ya kliniki itachambuliwa pamoja na data ya washiriki wengine. Takwimu za ujumbe kukuhusu zitaweza kuchambuliwa na wadhamini wa utafiti, bodi ya usalama na ukaguzi, watafiti wengine, na pia inaweza kuwekwa kwenye hifadhidata ya umma, kulinga na utaaratibu kuhusu data, ulioko sasa. Ikiwa data kukuhusu itashirikishwa, wafanyikazi wa utafiti watalinda maelezo binafsi kukuhusu, na hakuna mtu atakayeweza kuunganisha taarifa zako (data) na habari yoyote itakayo weza kufanya ugunduliwe(K.v. jina lako, siku ya kuzaliwa..n.k). Taarifa inayo weza kukutambulisha itabadilishwa na nambari maalum ya utafiti,na ni watafiti na wataalam wa kliniki pekee watakao weza kuunganisha taarifa binafsi, na habari husika.

1. **Fidia/Malipo:**

Washiriki watapokea shilingi 200 (Dola 2 za Marekani) kwa kukamilisha kila uchunguzi (Wakati wa usajili, kujifungua, baaada ya kujifungua). Pia, shlingi 200 zitatopeanwa kwa kipimo cha kiwango cha virusi baada ya kujifungua, kwa sababu, kipimo hiki hakiambatani na mwongozo wa kitaifa nchini Kenya. Mwishowe, shilingi 200 zitaongezwa kwa vipimo vya virusi baada ya kujifungua, kitakacho tolewa nje ya hosptali (Wakati wa kutembelewa nyumbani). Kwa jumla, washiriki wanaweza kupokea hadi shilingi 1000 za Kenya (Dola 10 za Marekani).

1. **Ushiriki:**

Una haki ya kubadili msimamo wako na kutoendelea na utafiti hata baada ya kukubali kushiriki.

1. **Ufadhili na Kukomesha Mapema:**

Kila mwaka, bodi ya ukaguzi itakagua data ya utafiti ili kuhakikisha usalama wa mshiriki. Endapo bodi hii itaona usalama wa mshiriki ume atharishwa, utafiti unaweza kusimamishwa mapema. Wakati utafiti utafikia 2/3 kukamilika), kikundi huru cha watafiti, watachambua matokeo ya mapema ya utafiti. Ikiwa faida ya uchambuzi huu wa mapema inaonekana wazi, wanaweza kupendekeza kufunga utafiti mapema. Mfadhili wa utafiti akikatiza fedha mapema, utafiti utasimamishwa mapema. Ikiwa utafiti utahitaji kusimamishwa mapema kwa sababu yoyote, washiriki, watafanya kazi wa utafiti, na wahudumu wa kliniki, watashirikiana kuhakikisha kwamba washiriki wote wanapata huduma kamili, pasi kutatiza taratibu za PMTCT.

1. **Mawasiliano:**

a. Kwa maswali yoyote au wasiwasi juu ya utafiti au tukio la jeraha linalohusiana na utafiti, tafadhali wasiliana na Mpelelezi mkuu, May Maloba - 0720254069.

b. Endapo una maswali yanayohusu haki kama mshiriki wa utafiti, unaweza kuwasiliana na: Mwenyekiti wa kamati, KEMRI Scientific and Ethics Review Unit, P. O. Box 54840-00200, Nairobi; Nambari za simu: 020-2722541, 0717719477; Barua pepe: seru@kemri.org

1. **Chaguzi na Sahihi:** Tafadhali hakikisha mshiriki ameweka sahii/kidole kwa KILA MOJA WAPO ya sehemu zifuatazo.
2. **Dhibitisho la utafiti kwa sasa:**

___________ Ninakubali kushiriki katika utafiti huu kwa sasa

___________ Nimekataa kushiriki katika utafiti huu kwa sasa

1. **Kushiriki kwa Takwimu:**

___________ Nakubali kutumika kwa habari zangu na watafiti wengine pasipo idhini ya ziada, bora tu vitambulisho vimeondolewa.

___________ Nimekataa kutumika kwa habari zangu na watafiti wengine pasipo idhini yangu ya ziada, kukiwepo/kutokuwepo vitambulisho.

1. **Utafiti wa Baadaye**

_______Ninakubali kutumika kwa habari yangu au sampuli yangu kwa tafiti zijazo au zishirikishwe na watafiti wengine bila indini ya ziada bora tu vitambulisho vimeondolewa.

_______ Nimekataa kutumika kwa habari yangu au sampuli yangu itumike kwa utafiti ujao, au zishirikiwe na watafiti wengine bila indini yangu ya kukiwepo/ kutokuwepo vitambulisho.

**SAHIHI YA MSHIRIKI**

________________________________________

Jina la Mshiriki

________________________________________ ______________

Sahihi ya mshiriki Tarehe

**_______________________________________________________________­­­­­­­­­­­­­­­­­­**

**Anwani ya Mshiriki (Address)**

***(Sehemu ifuatayo kutumika kuweka sahihi ya msimamizi, mzazi, na Kaimu mzazi pekee)***

Sahihi yako hapo chini inaashiria kwamba uko na mamlaka kisheria kutoa uamuzi kwa niaba ya mshiriki, na umesoma jalada hili. Utapewa kopi ya jalada. *(Mtafiti mkuu ndio mwenye mamlaka ya kuthibitisha anaye dai, ako na mamlaka ya kisheria kulingana na sheria za gatuzi/taifa)*.

**SAHIHI YA MLEZI/KAIMU ANAYETAMBULIWA NA SERIKALI**

_______________________________________

Jina la mlezi/Kaimu (tumia herufi kubwa)

_______________________________________

Uhusiano na mshiriki

________________________________________ ______________

Sahihi ya mlezi/Kaimu Tarehe

**SAHIHI YA SHAHIDI**

_______________________________________

Jina la mshahidi (tumia herufi kubwa)

________________________________________ ______________

Sahihi ya mshahidi Tarehe

**SAHIHI YA MTAFITI ANAYEPEWA IDHINI:**

_

______________________________________

Jina la Mtafiti

________________________________________ ______________

**INFORMED CONSENT DOCUMENT: INTERVENTION SITE- LUO**

**Wi nonro:** Evaluating the HITSystem to improve PMTCT retention and maternal viral suppression in Kenya

**Jo nonro(s) – Mawuok kae kod mawuok oko:**

**Jo nonro mokwongo:** May Maloba MCH; Sharon Mokua, MSc; Dr. Sarah Finocchario Kessler, PhD, MPH

**Jo ma konyo jo nonro:** Natabhona Mabachi, PhD; Brad Gautney, MPH; Kathy Goggin, PhD; Vincent Staggs, PhD

**Kama itimoe nonro:** Kenya.

Ikwayi mondo indojie nonro. Pinyka ondik ni gik ma dwarore ni mondo ing’e kapok idonjo e nonro. Wabiro chiwo ler kod weche mang’ey pinyka. Kiyie to penj penjo moro a mora kuom wach ma idwa ngeyo kapok ichako nonro.Bende inyalo wuoyo kod jomamoko ( kaka anyuola, osiepe kod jathieth) kuom nonroni, kapok idonjo.

| **Weche Mokwongo ma onego Ing’e (Not more than 500 words)** |
| --- |
| - **Hero mari ne chiwo**. Ikwayi ni mondo ichiwri e nonro. En hero mari ni mondo ichwri kata inyalo tamri. Onge rach kata onge gima ibiro wito ka ok diher bedo e nonro, kata ka isedonjo to iweyo. - **Ang’o Ma omiyo**.HITSystem en chenro mamedo siro thieth mar PMTCT kod EID chenro ni timre kod sms maparone joma mine kod joma chiwo thieth.Nonro ni biro temo ng’iyo ka bende HITSystem nyalo siro malong’o thieth mar PMTCT kod siro kaka kute mag ayaki inyalo dwokie piny kuom mine mayach kasto kendo bang nyuol. - **Ndalo.** Ibiro bedo e nonro nyaka bang dweche auchiel ka ose nyuol nyathi. - **Gigo ma mabiro timore.** Ka iyie donjo e nonro,ibiro bedo jachiw nonro mar intervention site. Ibiro penj penjo moko mokwongo seche ma irwaki e nonro, seche ma inyuol kod bang dweche auchiel ka ise nyuol. I biro rwaki e hitsystem mondo okonyi kod thieth mar PMTCT nyaka ichop dweche auchiel ka isenyuol. Gigo ma ibiro yudo gin SMS maparoni sa mwonyo yath, paroni chieng ma onego iduogi e thieth, paroni ni mondo ibi I inyuol e hospital ka saa ochopo, kendo biro miyo nyathini ma inyuolo mondo omi pim kute mag ayaki e saa madwarore. Bende ibiro pim kiwango mag kute mag ayaki e rembi, kendo pim machielo ibiro tim bang wige angwen bang nyuol. Dwoko mag VL ibiro ti godo e nonro kende to ok bi nyis jothieth ma thiedhi. Ka dipo ni pim mar VL sa nyuol kata seche mamoko kaka dwarore , jo tim nonro nyalo manyi e yore ma opogore pogore, gibiro limi e dalani mondo one kaka inyalo yudo pim. Limbe go ibiro luogni ngima nyathi mondo weche ma opondo kik ngere, to VL ibiro kaw man aka onge ngama ongeyo kendo ka iseyie.Limbe makamago bende inyal pangi chieng machiello ka oneni weche mopondo nyalo fwenyore. Bang tieko nonro, dwoko mari kod jomamoko ibiro tim negi analysis. - **Hinyruok.** Hinyruok manyalo bedie en ngeyo chal mari mag kute mag ayaki , to seche mamoko jachiw nonro nyalo bedo kod lit ma iye. Wa kaw okang mondo gik ma kamago kik. Weche ma iwacho ok bi riw kod nyingi kata yo mora mora ma inyalo ngeyi godo.Weche nonro mag kila ngato ibiro rit kod jo tim nonro kod jo od thieth, jogi duto osepuonji kaka onego gi rit weche nonro e yo makare, kendo gibiro neon ni weche ma iye kendo ma opondo ok owuok oko seche duto. Jal ma otelo ne nonro nikod tiegruok mar hocho ma onyalo konyi kod hocho seche ma in kod lit ma iye,Bende jo community health workers biro limi ka **Benefits**. If you enroll in this study, it means that you will benefit from more frequent communication and support for the multiple steps in the PMTCT process. This can improve the chances of having a HIV-free infant. - **Yore Mamoko.** Kar mondo idonjie e nonro, inyalo (1) Yudo thieth mar PMTCT e hospital, maonge konyruok mar HITSystem, kata (2) Iyudo konyruok mar HITSystem e thieth mar PMTCT ma wechegi ok ketie data analysis |

**Tij Nonro:** Briefly describe the purpose of the study.

1. **Kaka Nonro Nitie:**

Hosiptal ni oyier kuom mamoko mondo obed kaka intervention site. Mano nyiso ni ka iyie donjo e nonro, to ibiro bedo kaka jachiw nonro e intervention site. Ibiro kwayi mondo idwok penjo seche ma idonjo e nonro, seche ma inyuol,kod band dweche auchiel ka ise nyuol. Penjo biro kawo saa madirom dakika 30 to ibiro penjie kod jachiw thieth e hospital kama opondo. Inyalo weyo dwoko penjo kendo inyalo wuok e nonro saa a saya ma idwaro

Ibiro yudo konyruok koa kuom HITSystem-e thieth mar PMTCT nyaka ichop dweche auchiel bang ka ise nyuol. Ibiro yudo thieth madwarore mar PMTCT.HITSystem en yo ma medo miyo ng’ama miyo kod nyathine mondo oyud thieth mar PMTCT e yo makare. HITSystem biro ooroni sms mondo okonyi kod paroni saa mwonyo yath, paroni biro e od thieth, iki ne nyuol mar hospital, kod konyo nyathini mondo oyud pim mar kute mar ayaki . Obiro nyiso ja thieth ka opo ni ikoso biro e thieth mar PMTCT, kata ka saa mari mar VL ochopo, mondo oporni okaw remo..

Bende kiweyo pim VL kaka chike ma Kenya wacho, Ibiro kwayi mondo ichiw rembi ne pim mar VL bang wige ang’wen ka ise nyuol. Dwoko mar pim VL ibiro ti godo e nonro kende, onge jathieth moro a mora ma ibiro mi nonro gi. Ka dipo ni pim mar VL ok otim sama inyuol kata kaka chik wacho to jo nonro kata ja chiw thieth nyalo manyi e yore ma opogore opogore, kaka biro limi e dalani mondo one kaka diyud pim. Limbe gi wabiro luongo ni limbe mag ng’ima nyathi mondo obed gima opondo kendo ma ok ong’ere.Pim ibiro mana tim ka osene ni en gima opondo kendo mana ka iyie.Ka oneni ok en gima opondo to ibiro pang odichieng machielo.

Nonro biro dhi mbele nyaka nyathini chop dweche auchiel. Bang kanyo ibiro yiero ka idwaro dhi mbele kod yudo ber HITSYstem kiluwo godo thieth nyathini, en mana ni kiyie to weche gi koro ok bi ti godo e nonro.

Ka nitie lokruok moro a mora e nonro to ibiro nyisi.

1. **Chuech maiye mar dhano maluwore**

Ok tim

1. **Kaka ibiro kan kod ting’o samples kod nonro mamoko:**

Kaka hospital nigi yorgi mar rito samples, Samples duto ma okaw e nonro ni, ibiro kaw kaka remo ma otwo kaeto ibiro ter e lab mondo olosi. Bange to ibiro kethgi. Samples go ok bi ti godo kendo e nonro mamoko.

1. **Hinyruok kata chandruok ma otudore kod nonro:**

Wan kod geno ni hinyruok nyalo bedo matin, bedo e nonro nyalo bedo kod hinyruok. Mana kaka nonro mag kute mag ayaki weche ma opondo kod chandruok nyalo bedo. Oket yore mondo ng’ato kik ng’e chal mari, samoro jachiw nonro nyalo bedo kod chandruok gi anyuola mare kata alwora mare. Lit ma iye nyalo betie ahinya ka ineno ka igolo remo kuom nyathini, to kod mine ma inyiso dwoko mar chal nyithidgi. Samoro ok inyal mor ka iwachonegi kaka dwoko nyathi chalo.Mine machiwo VL samples bende nyalo kod rem matin seche ma I golo remo kuomgi

1. **Ber Mantie:**

Nonro ni nitie kod ber manyalo konyi. HITSystem-biro konyo thieth mar PMTCT ne mine mondo one ni oluwore kod thieth. Ka thieth mar PMTCT kod pim malong’o mar VL oluw kaka dwarore nyalo miyo ng’imani bed maber kendo geng’o chiwo kute mag ayaki ne nyathi.Ka dipo ni nyathi oyudo kute mag ayaki to nyalo miyo nyathi pim chon kendo mondo ochake e yath mapiyo mapiyo, kendo mae nyalo medo bero ng’ima nyathi. Moloyo kendo e ni nonro ni biro medo siro weche PMTCT e pinywa mar Kenya.Dwokogi biro medo siro kendo nyiso yore ma inyalo kedgo e yore tieko kute mag ayaki kuom nyithindo.

1. **Yore pim mamoko kata thieth:**

In thuolo donjo kata tamori donjo e nonro ni. Ka ok idwa donjo e nonroni, ma ok biketho kit thieth mar PMTCT kod EID ma isebedo ga ka iyudo e od thieth.Bende in kod thuolo seche te mar loko pachi mar weyo nonro kata ka ne iseyie.

1. **Weche ma opondo:**

Wecheni ma opondo en gima wamiyo luor kendo waketo okang’ ma opogore opogore mondo weche ni ma iye kik ng’ere ndalo duto ma nonro ni dhi mbele. Okang bende ne okaw seche mane iloso HITSYstem mondo wayud thuolo mar oorni sms ma ok ogolo chal mari kata mar nyathi ne ng’at a ng’ata. Messages ma oorni kod HITSystem okbi nyiso chal mari kata ka obedo ni message aa e hospital. Joma nikod thuolo mar neno HITSystem kod gik manie e yie en mana jo nonro matimo nonro kende.

Bang tieko nonro, dwokogi ka oriwore kod mag jomamoko ibiro ng’i e yo matut,bang’e to ibiro nyis jogo manyalo konyo kod siro weche thieth,dwokogi ibiro kan e yo malong’o, kata miyo jo nonro moko , bange ibiro kan gi kama ber. Ka dwokogi dipo onyis jomamoko, to jo nonro biro pando wecheni duto kendo ong’e nga’ma biro ng’eyo ni in ema ne idwoko wechego (nyingi, cheing nyuol kod mamoko) ibiro pand e yo ma opondo. Jo nonro kende ema biro ngeyo dwoko magi kendo riwo kod duoko mane ichiwo.

1. **Chudo:**

Ja chiw nonro biro yudo siling mia ariyo ka idwoko penjo seche mag enrollment,nyuol kod band dweche auchiel Bende siling mia ariyo ibiro chiw mar pim mar VL mar nyuol.nikech ok en pim mar chik.Mogik siling mia ariyo ibiro chiw ne pim mar VL mar dweche auchiel.Duto te, ja chiw nonro nyalo yudo siling eluf achiel.

**Bedo nie Nonro:**

Ka iyie donjo e nonro,inyalo yiero kit weche madiher yudo koa kuom HITSystem: Kony kod luwo thieth mar ART , Paroni dok e od thieth, kod paroni mondo idhi inyuo e od thieth. Bende ibiro yiero kaka diher yudo sms maparoni luwo thieth (pile wik ka wik,dwe ka dwe, k aka ok idwar).

In kod ratiro mar loko pachi ka idwa wuok e nonro kata ka ne iseyie.

1. **Kony kod chungo nonro chon:**

Higa ka higa joma ng’iyo nonro biro ng’iyo ka jachiw nonro nikare.Every year, a review board will review study data to ensure participant safety. Ka gineno ni wechene jachiw nonro owuok oko to nonro ibiro chung mapiyo. Kendo nonro kadhi rumo, jo nonro ma ok oriwore kodwa biro ng’iyo dwoko e yo matut. Ka giyudo ni dwoko nonro ni kod ber mang’ey to ginyalo wacho mondo ochung nonro chon. Ka dipo jalo ma ogolo omenda oweyo, nonro bende inyalo chung. Ka dipo ni nonro dwa chung chon jo nonro biro tiyo kod jo thieth mondo gine ni jachiw nonro oyudo thieth mar PMTCT ma ok ochung**.**

1. **To ka in kod penjo e nonro ni:**

Ka in kod penjo moro a mora kata wach moro a more e wi nonro ni , kata ng’ato ohinyore nikech nonro ni to wuo kod The principal investigator, May Maloba at 0720254069.

To ka in kod penjo e wi nonro ni kata ratiro mari, Tudri kod: The Committee Chairperson, KEMRI Scientific and Ethics Review Unit, P. O. Box 54840-00200, Nairobi; Telephone numbers: 020-2722541, 0717719477; Email address: seru@kemri.org

1. **Ayie kod Seyi :** Kiyie to ne ni jachiwre e nonro oketo seyi kata Lwete. Wach achiel kuom weche gi:
2. **Ayie nonro ma sani**

___________ Ayie ni mondo adonji e nonro

___________ A dagi ni ok adonj e nonro

1. **Chiwo wechaga oko**

___________ Ayie mondo weche na oriw kod jotim nonro mamoko maonge yiena machielo mak mana ka ogol wechena manyalo fwenya

___________ Adagi mondo weche na kik riw kod jotim nonro mamoko maonge yiena machielo mak mana ka ogol wechena manyalo fwenya

1. **Nonro Ma mbele**

______Ayie ni mondo weche na oti godo e nonro mabiro kendo onyis jo nonro mamoko maonge yiena machielo tek ni wechega ok bi mi fwenya

_______Adagi ni wechega kik bi ti godo e nonro mabiro kendo kik nyis jo nonro mamoko maonge yiena machielo manyalo miyo fwenya

**Seyi jachiw nonro**

________________________________________

Nying jachiw nonro

________________________________________ ______________

Seyi jachiw ninro Tarik

**_______________________________________________________________­­­­­­­­­­­­­­­­­­**

**Kama idakie am intie pile**

***(****Use the following signature blocks for representative, parents, and guardians, only if applicable****)***

Your signature below indicates you are legally authorized to act on behalf of the participant, and have read this document. You will receive a copy of this document. *(The Principal Investigator is responsible for confirming that an individual is a Legally Authorized Representative based on local and state laws.)*

**SIGNATURE OF LEGALLY AUTHORIZED REPRESENTATIVE**

_______________________________________

Printed Name of Legally Authorized Representative

_______________________________________

Relationship to the Participant

________________________________________ ______________

Signature of Legally Authorized Representative Date

*(Remove the witness signature if this study is conducted under ICH GCP. Determine if your institution requires witness to the entire consent process or only witness to the final signature.)*

**Seyi janeno**

(This individual can be a relative of the participant, but cannot be an individual involved with the research study.)

_______________________________________

Nying Janeno

________________________________________ ______________

Seyi Janeno Tarik

**Seyi Janonro**

(Can only be signed by an investigator or staff approved to administer consent)_

______________________________________

Nying Janonro

________________________________________ ______________

Seyi Janonro Tarik

**INFORMED CONSENT DOCUMENT: CONTROL SITE- LUO**

**Wi nonro:** Evaluating the HITSystem to improve PMTCT retention and maternal viral suppression in Kenya

**Jo nonro(s) – Mawuok kae kod mawuok oko:**

**Jo nonro mokwongo:** May Maloba MCH; Sharon Mokua, MSc; Dr. Sarah Finocchario Kessler, PhD, MPH

**Jo ma konyo jo nonro:** Natabhona Mabachi, PhD; Brad Gautney, MPH; Kathy Goggin, PhD; Vincent Staggs, PhD

**Kama itimoe nonro:** Kenya

Ikwayi mondo indojie nonro. Pinyka ondik ni gik ma dwarore ni mondo ing’e kapok idonjo e nonro. Wabiro chiwo ler kod weche mang’ey pinyka. Kiyie to penj penjo moro a mora kuom wach ma idwa ngeyo kapok ichako nonro.Bende inyalo wuoyo kod jomamoko ( kaka anyuola, osiepe kod jathieth) kuom nonroni, kapok idonjo.

| **Key Information for You to Consider(Not more than 500 words)** |
| --- |
| - **Hero mari ne chiwo**. Ikwayi ni mondo ichiwri e nonro. En hero mari ni mondo ichwri kata inyalo tamri. Onge rach kata onge gima ibiro wito ka ok diher bedo e nonro, kata ka isedonjo to iweyo. - **Ang’o Ma omiyo**.HITSystem en chenro mamedo siro thieth mar PMTCT kod EID chenro ni timre kod sms maparone joma mine kod joma chiwo thieth.Nonro ni biro temo ng’iyo ka bende HITSystem nyalo siro malong’o thieth mar PMTCT kod siro kaka kute mag ayaki inyalo dwokie piny kuom mine mayach kasto kendo bang nyuol. - **Ndalo.** Ibiro bedo e nonro nyaka bang dweche auchiel ka ose nyuol nyathi. - **Gigo ma mabiro timore.** Ka iyie donjo e nonro,ibiro bedo jachiw nonro mar control site. Ibiro penj penjo moko mokwongo seche ma irwaki e nonro, seche ma inyuol kod bang dweche auchiel ka ise nyuol. I biro rwaki e hitsystem mondo okonyi kod thieth mar PMTCT nyaka ichop dweche auchiel ka isenyuol. Gigo ma ibiro yudo gin SMS maparoni sa mwonyo yath, paroni chieng ma onego iduogi e thieth, paroni ni mondo ibi I inyuol e hospital ka saa ochopo, kendo biro miyo nyathini ma inyuolo mondo omi pim kute mag ayaki e saa madwarore. Bende ibiro pim kiwango mag kute mag ayaki e rembi, kendo pim machielo ibiro tim bang wige angwen bang nyuol. Dwoko mag VL ibiro ti godo e nonro kende to ok bi nyis jothieth ma thiedhi. Ka dipo ni pim mar VL sa nyuol kata seche mamoko kaka dwarore , jo tim nonro nyalo manyi e yore ma opogore pogore, gibiro limi e dalani mondo one kaka inyalo yudo pim. Limbe go ibiro luogni ngima nyathi mondo weche ma opondo kik ngere, to VL ibiro kaw man aka onge ngama ongeyo kendo ka iseyie.Limbe makamago bende inyal pangi chieng machiello ka oneni weche mopondo nyalo fwenyore. Bang tieko nonro, dwoko mari kod jomamoko ibiro tim negi analysis. - **Hinyruok.** Hinyruok manyalo bedie en ngeyo chal mari mag kute mag ayaki , to seche mamoko jachiw nonro nyalo bedo kod lit ma iye. Wa kaw okang mondo gik ma kamago kik. Weche ma iwacho ok bi riw kod nyingi kata yo mora mora ma inyalo ngeyi godo.Weche nonro mag kila ngato ibiro rit kod jo tim nonro kod jo od thieth, jogi duto osepuonji kaka onego gi rit weche nonro e yo makare, kendo gibiro neon ni weche ma iye kendo ma opondo ok owuok oko seche duto. Jal ma otelo ne nonro nikod tiegruok mar hocho ma onyalo konyi kod hocho seche ma in kod lit ma iye,Bende jo community health workers biro limi ka **Benefits**. If you enroll in this study, it means that you will benefit from more frequent communication and support for the multiple steps in the PMTCT process. This can improve the chances of having a HIV-free infant. - **Yore Mamoko.** Kar mondo idonjie e nonro, inyalo (1) Yudo thieth mar PMTCT e hospital, maonge konyruok mar HITSystem, kata (2) Iyudo konyruok mar HITSystem e thieth mar PMTCT ma wechegi ok ketie data analysis |

**Tij Nonro:** Briefly describe the purpose of the study.

1. **Kaka Nonro Nitie:**

Hosiptal ni oyier kuom mamoko mondo obed kaka intervention site. Mano nyiso ni ka iyie donjo e nonro, to ibiro bedo kaka jachiw nonro e intervention site. Ibiro kwayi mondo idwok penjo seche ma idonjo e nonro, seche ma inyuol,kod band dweche auchiel ka ise nyuol. Penjo biro kawo saa madirom dakika 30 to ibiro penjie kod jachiw thieth e hospital kama opondo. Inyalo weyo dwoko penjo kendo inyalo wuok e nonro saa a saya ma idwaro

Ibiro yudo konyruok koa kuom HITSystem-e thieth mar PMTCT nyaka ichop dweche auchiel bang ka ise nyuol. Ibiro yudo thieth madwarore mar PMTCT.HITSystem en yo ma medo miyo ng’ama miyo kod nyathine mondo oyud thieth mar PMTCT e yo makare. HITSystem biro ooroni sms mondo okonyi kod paroni saa mwonyo yath, paroni biro e od thieth, iki ne nyuol mar hospital, kod konyo nyathini mondo oyud pim mar kute mar ayaki . Obiro nyiso ja thieth ka opo ni ikoso biro e thieth mar PMTCT, kata ka saa mari mar VL ochopo, mondo oporni okaw remo..

Bende kiweyo pim VL kaka chike ma Kenya wacho, Ibiro kwayi mondo ichiw rembi ne pim mar VL bang wige ang’wen ka ise nyuol. Dwoko mar pim VL ibiro ti godo e nonro kende, onge jathieth moro a mora ma ibiro mi nonro gi. Ka dipo ni pim mar VL ok otim sama inyuol kata kaka chik wacho to jo nonro kata ja chiw thieth nyalo manyi e yore ma opogore opogore, kaka biro limi e dalani mondo one kaka diyud pim. Limbe gi wabiro luongo ni limbe mag ng’ima nyathi mondo obed gima opondo kendo ma ok ong’ere.Pim ibiro mana tim ka osene ni en gima opondo kendo mana ka iyie.Ka oneni ok en gima opondo to ibiro pang odichieng machielo.

Nonro biro dhi mbele nyaka nyathini chop dweche auchiel. Bang kanyo ibiro yiero ka idwaro dhi mbele kod yudo ber HITSYstem kiluwo godo thieth nyathini, en mana ni kiyie to weche gi koro ok bi ti godo e nonro.

Ka nitie lokruok moro a mora e nonro to ibiro nyisi.

1. **Chuech maiye mar dhano maluwore**

Ok tim

1. **Kaka ibiro kan kod ting’o samples kod nonro mamoko :**

Kaka hospital nigi yorgi mar rito samples, Samples duto ma okaw e nonro ni, ibiro kaw kaka remo ma otwo kaeto ibiro ter e lab mondo olosi. Bange to ibiro kethgi. Samples go ok bi ti godo kendo e nonro mamoko.

1. **Hinyruok kata chandruok ma otudore kod nonro:**

Wan kod geno ni hinyruok nyalo bedo matin, bedo e nonro nyalo bedo kod hinyruok. Mana kaka nonro mag kute mag ayaki weche ma opondo kod chandruok nyalo bedo. Oket yore mondo ng’ato kik ng’e chal mari, samoro jachiw nonro nyalo bedo kod chandruok gi anyuola mare kata alwora mare. Lit ma iye nyalo betie ahinya ka ineno ka igolo remo kuom nyathini, to kod mine ma inyiso dwoko mar chal nyithidgi. Samoro ok inyal mor ka iwachonegi kaka dwoko nyathi chalo.Mine machiwo VL samples bende nyalo kod rem matin seche ma I golo remo kuomgi

.

1. **Ber Mantie:**

Onge ber ma ibiro yudo kod bedo e nonro. To,dwoko mag nonro ibiro ti godo e siro thieth mag PMTCT Kenya kae.is data will improve strategies to meet the global goal of eliminating new HIV infections in children through PMTCT services.

1. **Yore pim mamoko kata thieth:**

In thuolo donjo kata tamori donjo e nonro ni. Ka ok idwa donjo e nonroni, ma ok biketho kit thieth mar PMTCT kod EID ma isebedo ga ka iyudo e od thieth.Bende in kod thuolo seche te mar loko pachi mar weyo nonro kata ka ne iseyie.

1. **Weche ma Opondo:**

Wecheni ma opondo en gima wamiyo luor kendo waketo okang’ ma opogore opogore mondo weche ni ma iye kik ng’ere ndalo duto ma nonro ni dhi mbele. Okang bende ne okaw seche mane iloso HITSYstem mondo wayud thuolo mar oorni sms ma ok ogolo chal mari kata mar nyathi ne ng’at a ng’ata. Messages ma oorni kod HITSystem okbi nyiso chal mari kata ka obedo ni message aa e hospital. Joma nikod thuolo mar neno HITSystem kod gik manie e yie en mana jo nonro matimo nonro kende.

1. **Chudo:**

Ja chiw nonro biro yudo siling mia ariyo ka idwoko penjo seche mag enrollment,nyuol kod band dweche auchiel Bende siling mia ariyo ibiro chiw mar pim mar VL mar nyuol.nikech ok en pim mar chik.Mogik siling mia ariyo ibiro chiw ne pim mar VL mar dweche auchiel.Duto te, ja chiw nonro nyalo yudo siling eluf achiel.

1. **Bedo nie nonro:**

In kod ratiro mar loko pachi ka idwa wuok e nonro kata ka ne iseyie.

1. **Kony kod chungo nonro chon:**

Higa ka higa joma ng’iyo nonro biro ng’iyo ka jachiw nonro nikare.Every year, a review board will review study data to ensure participant safety. Ka gineno ni wechene jachiw nonro owuok oko to nonro ibiro chung mapiyo. Kendo nonro kadhi rumo, jo nonro ma ok oriwore kodwa biro ng’iyo dwoko e yo matut. Ka giyudo ni dwoko nonro ni kod ber mang’ey to ginyalo wacho mondo ochung nonro chon. Ka dipo jalo ma ogolo omenda oweyo, nonro bende inyalo chung. Ka dipo ni nonro dwa chung chon jo nonro biro tiyo kod jo thieth mondo gine ni jachiw nonro oyudo thieth mar PMTCT ma ok ochung**..**

1. **To ka in kod penjo e nonro ni :**

a. Ka in kod penjo moro a mora kata wach moro a more e wi nonro ni , kata ng’ato ohinyore nikech nonro ni to wuo kod The principal investigator, May Maloba at 0720254069.

To ka in kod penjo e wi nonro ni kata ratiro mari, Tudri kod: The Committee Chairperson, KEMRI Scientific and Ethics Review Unit, P. O. Box 54840-00200, Nairobi; Telephone numbers: 020-2722541, 0717719477; Email address: [seru@kemri.org](mailto:seru@kemri.org)

*(Signature blocks for participant and individuals administering consent must be part of all forms. Other signature blocks will be included when appropriate, as when the research study involves children, surrogate consent etc.)*

1. **Ayie kod Seyi :** Kiyie to ne ni jachiwre e nonro oketo seyi kata Lwete. Wach achiel kuom weche gi.
   1. **Ayie nonro ma sani**

___________ Ayie ni mondo adonji e nonro

___________ A dagi ni ok adonj e nonro

- 1. **Chiwo wechaga oko**

___________ Ayie mondo weche na oriw kod jotim nonro mamoko maonge yiena machielo mak mana ka ogol wechena manyalo fwenya

___________ Adagi mondo weche na kik riw kod jotim nonro mamoko maonge yiena machielo mak mana ka ogol wechena manyalo fwenya

1. **Nonro Ma mbele**

_______Ayie ni mondo weche na oti godo e nonro mabiro kendo onyis jo nonro mamoko maonge yiena machielo tek ni wechega ok bi mi fwenya

_______Adagi ni wechega kik bi ti godo e nonro mabiro kendo kik nyis jo nonro mamoko maonge yiena machielo manyalo miyo fwenya

**Seyi jachiw nonro**

________________________________________

Nying jachiw nonro

________________________________________ ______________

Seyi jachiw ninro Tarik

**_______________________________________________________________­­­­­­­­­­­­­­­­­­**

**Kama idakie am intie pile**

***(****Use the following signature blocks for representative, parents, and guardians, only if applicable****)***

Your signature below indicates you are legally authorized to act on behalf of the participant, and have read this document. You will receive a copy of this document. *(The Principal Investigator is responsible for confirming that an individual is a Legally Authorized Representative based on local and state laws.)*

**SIGNATURE OF LEGALLY AUTHORIZED REPRESENTATIVE**

_______________________________________

Printed Name of Legally Authorized Representative

_______________________________________

Relationship to the Participant

_______________________________________ ______________

Signature of Legally Authorized Representative Date

*(Remove the witness signature if this study is conducted under ICH GCP. Determine if your institution requires witness to the entire consent process or only witness to the final signature.)*

**Seyi janeno**

(This individual can be a relative of the participant, but cannot be an individual involved with the research study.)

_______________________________________

Nying Janeno

________________________________________ ______________

Seyi Janeno Tarik

**Seyi Janonro**

(Can only be signed by an investigator or staff approved to administer consent)

________________________________________ ______________

Signature of Administering Individual Date

1. **Appendix II. Data collection materials**

Please note, the provider interview and facility assessment form will be available in English only. All other data collection materials will be available in English, Swahili and Luo.

**English translations**

**Appendix IIA. Enrollment Survey**

***For interviewer to fill out:***

Study ID: ______________

Date of interview__________________________

Interviewer initials_________________________

Mother Demographics

1. Mothers DOB: ________________
2. Mother phone number:____________________
3. Mother tracing information: ___________________________________________________________ _________________________________________________________________________________
4. Date of enrollment in PMTCT services:__________________
5. Estimated delivery date (EDD) ______________________
6. Date initiated HAART _______________
7. What is your **highest level of education**?
   1. No school
   2. Partial primary
   3. Completed primary
   4. Partial secondary
   5. Completed secondary
   6. University/college
8. Who have you **disclosed your HIV status to**?
   1. No one
   2. Partner/husband
   3. 1 or more family
   4. 1 or more friends
   5. 1 or more parent
9. How do you access a mobile phone?
   1. Own phone
   2. Shared, with someone who knows HIV status
   3. Shared, with someone who doesn’t know HIV status
   4. Borrow phone when needed
   5. No mobile phone access
10. Time to hospital (minutes)______________
11. Expense to hospital (roundtrip, KES)______
12. What is your **relationship status?**
    1. Single
    2. Married
    3. Unmarried, but in a relationship
    4. Separated
    5. Divorced
    6. Widowed
    7. Other, specify__________
13. What is your **living situation**?
    1. No current partner
    2. Live separate from partner
    3. Live with current partner
14. How many living children do you have?
    1. Biological____________
    2. Non-biological___________
15. Have you ever been through PMTCT and/or EID services before? ………Y / N

Partner support & Financial Control

1. What is your partner’s HIV status?
   1. HIV+
   2. HIV –
   3. Unknown
   4. No current partner
2. How did your partner react to the news of your pregnancy?
   1. Happy
   2. Neutral
   3. Unhappy
   4. Don’t know
   5. No current partner
3. Who usually makes the final decision regarding how money is spent in your household?
4. My partner, always
5. My partner more often than me
6. My partner and me equally
7. Me more often than my partner
8. Me, always.
9. No current partner
10. Average weekly household income
11. Less than 500 KES
12. 500-750 KES
13. 750-1,000 KES
14. 1,000-2,500 KES
15. >2,500 KES
16. I worry that I will not have enough money to reach the hospital for my own or my child’s care?
    1. Strongly agree
    2. Agree
    3. Disagree
    4. Strongly Disagree

Partner Support

1. How important is it to you that your male partner supports you in each of the following ways?

|  | Not important at all | Not very important | Somewhat important | Very important | N/A, no partner |
| --- | --- | --- | --- | --- | --- |
| 21a. Attend your PMTCT appointments with you? |  |  |  |  |  |
| 21b. Provide money for transport to the hospital? |  |  |  |  |  |
| 21c. Give you advice on remaining healthy while pregnant? |  |  |  |  |  |
| 21d. Help you with household responsibilities so that you can rest? |  |  |  |  |  |
| 21e. Help you with household responsibilities so that you can attend PMTCT appointments? |  |  |  |  |  |
| 21f. Remind you to take your medication? |  |  |  |  |  |
| 21g. Remind you of the importance of following doctor’s instructions? |  |  |  |  |  |
| 21h. Provided money to purchase medication or vitamins? |  |  |  |  |  |
| 21i. Attend the delivery? |  |  |  |  |  |
| 21j. Provide encouragement |  |  |  |  |  |

1. In what ways do you expect your male partner to support you during pregnancy?

|  | Do not expect | Unsure | Expect | N/A – no partner |
| --- | --- | --- | --- | --- |
| 22a. Attend your PMTCT appointments with you? |  |  |  |  |
| 22b. Provide money for transport to the hospital? |  |  |  |  |
| 22c. Give you advice on remaining healthy while pregnant? |  |  |  |  |
| 22d. Help you with household responsibilities so that you can rest? |  |  |  |  |
| 22e. Help you with household responsibilities so that you can attend PMTCT appointments? |  |  |  |  |
| 22f. Remind you to take your medication? |  |  |  |  |
| 22g. Remind you of the importance of following doctor’s instructions? |  |  |  |  |
| 22h. Provided money to purchase medication or vitamins? |  |  |  |  |
| 22i. Attend the delivery? |  |  |  |  |
| 22j. Provide encouragement |  |  |  |  |

Depression

Please check the answer that comes closest to how you have felt IN THE PAST 7 DAYS, not just today.

1. I have looked forward with enjoyment to things
   1. As much as I ever did
   2. A little less than I used to
   3. Much less than I used to
   4. Not at all
2. I have blamed myself unnecessarily when things went wrong
3. Yes, often
4. Yes, some of the time
5. Not very often
6. No, never
7. I have felt scared or panicky for no good reason
8. Yes, quite a lot
9. Yes sometimes
10. No, not much
11. No, not at all
12. I have been so unhappy that I have been crying
13. Yes, most of the time
14. Yes, quite often
15. Only occasionally
16. No, never
17. The thought of harming myself has occurred to me
18. Yes, often
19. Sometimes
20. Hardly ever
21. Never

Risk of Violence

For each of the following statements (27-30), choose the answer which best indicates how often each has occurred in the past 12 months

|  | Never | Rarely | Sometimes | Frequently |
| --- | --- | --- | --- | --- |
| 1. In the past 12 months, has your partner ever insulted you or made you feel bad about yourself? |  |  |  |  |
| 1. In the past 12 months, has your partner ever hit you, kicked you, dragged you, or beaten you up? |  |  |  |  |
| 1. In the past 12 months, has your partner ever hit, kicked, dragged or beaten your child? |  |  |  |  |
| 1. In the past 12 months, has your partner ever physically forced you to have sexual intercourse when you did not want to? |  |  |  |  |

PMTCT Knowledge

Please choose the best answer:

1. During pregnancy, I should receive viral load testing:
   1. Never, viral load testing is only conducted after delivery
   2. Between 1 and 2 times.
   3. At least three times.
   4. Don’t know
2. During pregnancy, it is recommended that HIV+ women take ART medications only if they are feeling unwell………………………True / False
3. It is recommended that I attend a minimum of how many ANC appointments?
4. 2
5. 3
6. 4
7. 5

PMTCT Motivation and self-efficacy

Please indicated the degree to which you agree or disagree with each of the following statements.

|  | Strongly Disagree | Disagree | Neutral | Agree | Strongly Agree |
| --- | --- | --- | --- | --- | --- |
| 1. I know I can maintain perfect adherence to my medication throughout my pregnancy, even when other people are around. |  |  |  |  |  |
| 1. It is very important to me that I deliver my child in a health facility, rather than at home. |  |  |  |  |  |
| 1. I am worried that I will not be able to find transport to the health facility when it is time for me to deliver my child. |  |  |  |  |  |
| 1. Other commitments – such as work, household duties, childcare – may prevent me from attending all of my ANC/PMTCT appointments. |  |  |  |  |  |
| 1. Attending all ANC/PMTCT appointments is extremely important to me. |  |  |  |  |  |

COVID-related questions

Please indicated the degree to which you expect COVID-19 to impact the following events.

|  | Strongly Disagree | Disagree | Neutral | Agree | Strongly Agree |
| --- | --- | --- | --- | --- | --- |
| 1. I delayed coming for ANC care because of concerns related to COVID-19 exposure. |  |  |  |  |  |
| 1. I delayed coming to care because someone in my household or I was feeling unwell and did not want to risk exposing others to illness. |  |  |  |  |  |
| 1. I do not want to attend the clinic as frequently for antenatal care because I am worried about COVID-19 exposure |  |  |  |  |  |
| 1. I am worried that delivering in a hospital will expose me and my child to COVID-19 |  |  |  |  |  |
| 1. Travel to and from the hospital has become more challenging (cost and/or availability of cars) as a result of COVID-19 |  |  |  |  |  |

Thank you for your time and participation.

**Appendix IIB. Delivery survey (within 4 weeks, Intervention and Control Sites)**

***For interviewer to fill out:***

Study ID: ______________

Date of interview__________________________

Interviewer initials_________________________

Delivery

1. Where did you deliver your child?
   1. Health care facility
      1. If so, please specify which one ___________________
   2. Traditional birth attendant
      1. If so, please specify location of delivery _______________
   3. Home
   4. Other
      1. Please specify_________
2. Approximately how long did you labor to deliver your child**?**
   1. < 2 hours
   2. 2-5 hours
   3. 6-12 hours
   4. > 12 hours
3. Did you or the infant experience any complications**?**
   1. yes
      1. Please explain for mother and/or infant separately: ________________________

_________________________________________________________________

- 1. no

Partner status & Disclosure

1. What is your **relationship status?**
   1. Single
   2. Married
   3. Unmarried, but in a relationship
   4. Separated
   5. Divorced
   6. Widowed
   7. Other, specify________
2. What is your **living situation**?
   1. No current partner
   2. Live separate from partner
   3. Live with current partner
3. Is your current partner the infant’s father?...........Y / N
4. Who have you **disclosed your HIV status to**? Select all that apply.
   1. No one
   2. Partner/husband
   3. 1 or more family
   4. 1 or more friends
   5. 1 or more parent
5. What is your partner’s HIV status?
   1. HIV+
   2. HIV –
   3. Unknown
   4. No current partner
6. Who usually makes the final decision regarding how money is spent in your household?
7. My partner, always
8. My partner more often than me
9. My partner and me equally
10. Me more often than my partner
11. Me, always.
12. No current partner
13. Average weekly household income
14. Less than 500 KES
15. 500-750 KES
16. 750-1,000 KES
17. 1,000-2,500 KES
18. >2,500 KES
19. I worry that I will not have enough money to reach the hospital for my own or my child’s care?
    1. Strongly agree
    2. Agree
    3. Disagree
    4. Strongly Disagree

Partner Support

1. How often *during your pregnancy* did your male partner support you in the follow ways:

|  | Never | Rarely | Sometimes | Often |
| --- | --- | --- | --- | --- |
| 12b. Provided money for transport to the hospital? |  |  |  |  |
| 12c. Gave you advice on remaining healthy while pregnant? |  |  |  |  |
| 12d. Helped you with household responsibilities so that you could rest? |  |  |  |  |
| 12e. Helped you with household responsibilities so that you could attend PMTCT appointments? |  |  |  |  |
| 12f. Reminded you to take your medication |  |  |  |  |
| 12g. Reminded you of the importance of following doctor’s instructions. |  |  |  |  |
| 12h. Provided money to purchase medication or vitamins? |  |  |  |  |
| 12i. My husband gave me encouragement throughout my pregnancy |  |  |  |  |
| 12j. Me and my husband prayed together for a safe pregnancy and delivery |  |  |  |  |

1. Did your partner attend any PMTCT appointments with you?
   1. No, he did not attend any
   2. He attended 1
   3. He attended 2
   4. He attended 3 or more.
   5. N/A, no partner.
2. If #13 was no, what were the reasons he did not attend?
   1. He was working during my PMTCT appointments__________Yes / No
   2. He lives or works far away ____________________________Yes / No
   3. He felt unwelcomed/uncomfortable at the clinic____________ Yes / No
   4. I did not want him to attend ___________________________ Yes / No
   5. He did not want to attend _____________________________ Yes / No
   6. He had other responsibilities _______________________
   7. Other, please specify_________________________________
3. Did your male partner provide support for your child’s delivery? Please select the most accurate answer.
   1. I do not have a male partner
   2. No, my male partner did not provide any type of support for my child’s delivery.
   3. My male partner provided funding for me to get to the hospital, but did not come himself.
   4. My male partner escorted me to the hospital while I was in labor.
   5. My male partner visited me in the hospital after my child was born.
   6. My male partner both escorted me to the hospital while I was in labor AND visited me in the hospital after my child was born.

Depression

Please check the answer that comes closest to how you have felt IN THE PAST 7 DAYS, not just today.

1. I have looked forward with enjoyment to things
2. As much as I ever did
3. A little less than I used to
4. Much less than I used to
5. Not at all
6. I have blamed myself unnecessarily when things went wrong
7. Yes, often
8. Yes, some of the time
9. Not very often
10. No, never
11. I have felt scared or panicky for no very good reason
12. Yes, quite a lot
13. Yes sometimes
14. No, not much
15. No, not at all
16. I have been so unhappy that I have been crying
17. Yes, most of the time
18. Yes, quite often
19. Only occasionally
20. No, never
21. The thought of harming myself has occurred to me
22. Yes, often
23. Sometimes
24. Hardly ever
25. Never

Risk of Violence

For each of the following statements (21-24), choose the answer which best indicates how often each has occurred in the past 12 months.

|  | Never | Rarely | Sometimes | Frequently |
| --- | --- | --- | --- | --- |
| 1. In the past 12 months, has your partner ever insulted you or made you feel bad about yourself? |  |  |  |  |
| 1. In the past 12 months, has your partner ever hit you, kicked you, dragged you, or beaten you up? |  |  |  |  |
| 1. In the past 12 months, has your partner ever hit, kicked, dragged or beaten your child? |  |  |  |  |
| 1. In the past 12 months, has your partner ever physically forced you to have sexual intercourse when you did not want to? |  |  |  |  |

PMTCT Knowledge

1. Can a mothers help prevent HIV transmission to her infant in the following ways?

a. Taking all prescribed ARTs during pregnancy and breastfeeding....................................Yes / No / DK

b. Delivering in a health care facility, instead of at home……………....................................Yes / No / DK

c. Using infant formula when it is available and breastfeeding when it is not………………..Yes / No / DK

d. Giving the infant Nevirapine…………………………..........................................................Yes / No / DK

e. Giving the infant Cotrimoxazole…………………………………………………..…………...Yes / No / DK

f. Giving baby cow’s milk and eggs before 6 months of age to help him/her grow strong…Yes / No / DK

1. It is recommended that my infant receives his/her first HIV test
2. Within 2 weeks of birth
3. At 6 weeks of age
4. At 6 months of age
5. At 12 months of age
6. One s/he stops breastfeeding
7. Don’t know
8. It is recommended that babies continue being routinely tested for HIV until when:
   1. 6 months of age
   2. 12 months of age
   3. 18 months of age
   4. 2 years of age
   5. The baby stops breastfeeding

PMTCT Motivation

For each of the following, indicate the extent to which you agree or disagree with the statement.

SD=strongly disagree, D=disagree, N=neither agree nor disagree, A= agree, SA = strongly agree.

|  | SD | D | N | A | SA |
| --- | --- | --- | --- | --- | --- |
| 1. I know I can maintain perfect adherence to my medication while my baby is very young and breastfeeding. |  |  |  |  |  |
| 1. Giving my baby his/her medication is extremely important to his health |  |  |  |  |  |
| 1. Even if s/he does not want to take it, I know that I’ll be able to give every dose of his/her medication |  |  |  |  |  |
| 1. It is extremely important to get routine viral load tests while my baby is still breastfeeding. |  |  |  |  |  |
| 1. Other commitments – such as work, household duties, childcare – may prevent me from getting my baby tested by 6 weeks of age. |  |  |  |  |  |
| 1. Attending all of my child’s appointments is extremely important to me. |  |  |  |  |  |

COVID-related questions

Please indicated the degree to which you expect COVID-19 to impact the following events.

|  | Strongly Disagree | Disagree | Neutral | Agree | Strongly Agree |
| --- | --- | --- | --- | --- | --- |
| 1. I was worried that delivering in a hospital woud expose me and my child to COVID-19 |  |  |  |  |  |
| 1. I am worried that attending the hospital for infant testing will expose me and my child to COVID-19 |  |  |  |  |  |
| 1. During pregnancy, challenges with travel to and from the hospital (cost and/or availability of cars) as a result of COVID-19 caused meto delay or miss attending some ANC or medication refill appointments |  |  |  |  |  |
| 1. During pregnancy, I delayed or missed ANC or medication refill appointments because I was feeling unwell and did not want to risk exposing others to illness. |  |  |  |  |  |

ENGAGEMENT WITH HITSYSTEM - **INTERVENTION ONLY**

1. Since enrolling in ANC, have you received any SMS from the hospital?
   1. No, I have not received any SMS.
   2. Yes, I have received at least one SMS from the hospital but less than 3
   3. Yes, I have received between 3 and 5 SMS
   4. Yes, I have received more than 5 SMS
2. Have you received and SMS for any of the following reasons?
3. To check how you are doing…………………………………….Yes / No
4. To inform you of your next appointment date………………… Yes / No
5. To remind you to take ART medications……………………… Yes / No
6. To let you know your child was due for services………………Yes / No
7. Asking you to return to the hospital to be informed of a test result………… Yes / No
8. If yes to any option in number 22, please answer question 23 regarding your reaction to receiving the text messages, otherwise skip to 24:
9. I did not find the SMS helpful……………….……………………………….………..True / False
10. I found the SMS helpful to remember to take medication……………………..…..True / False
11. I was worried that someone would see the SMS and discover my status ……….True / False
12. The SMS helped me feel more connected to the hospital/my care provider…….True / False
13. The SMS helped me remember when my appointments were……………………True / False
14. Someone other than me read one of the SMS and asked me about it……………True / False
15. A family member or friend discovered my HIV status because of the SMS………True / False
16. The SMS gave me important information regarding my or my infant’s care………True / False
17. The SMS motivated me to continue PMTCT and/or EID care………………………True / False
18. Please select a preference for frequency of ART adherence messages going forward:
19. Never, please stop sending me adherence messages
20. Daily
21. Weekly
22. Every two weeks
23. Monthly

Thank you for your time and participation.

**Appendix IIC. 6 Month Postpartum survey (Intervention and Control Sites)**

***For interviewer to fill out:***

Study ID: ______________

Date of interview__________________________

Interviewer initials_________________________

Partner status & Disclosure

1. What is your **relationship status?**
   1. Single
   2. Married
   3. Unmarried, but in a relationship
   4. Separated
   5. Divorced
   6. Widowed
2. What is your **living situation**?
3. No current partner
4. Live separate from partner
5. Live with current partner
6. Who have you **disclosed your HIV status to**?
7. No one
8. Partner/husband
9. 1 or more family
10. 1 or more friends
11. 1 or more parent
12. What is your partner’s HIV status?
13. HIV+
14. HIV –
15. Unknown
16. No current partner
17. Is your current partner the infant’s father? ….. Y / N / NA
18. Who usually makes the final decision regarding how money is spent in your household?
19. My partner, always
20. My partner more often than me
21. My partner and me equally
22. Me more often than my partner
23. Me, always.
24. No current partner
25. Average weekly household income
26. Less than 500 KES
27. 500-750 KES
28. 750-1,000 KES
29. 1,000-2,500 KES
30. >2,500 KES
31. I worry that I will not have enough money to reach the hospital for my own or my child’s care?
    1. Strongly agree
    2. Agree
    3. Disagree
    4. Strongly Disagree

Partner Support (To be filled at 6 month VL test)

1. How often since your infant’s birth has your male partner supported you in the follow ways:

|  | Never | Rarely | Sometimes | Frequently |
| --- | --- | --- | --- | --- |
| 9a. Attended your child’s medical appointments? |  |  |  |  |
| 9b. Provided money for transport to the hospital? |  |  |  |  |
| 9c. Gave you advice on infant feeding or hygiene? |  |  |  |  |
| 9d. Helped you with household responsibilities so that you could rest? |  |  |  |  |
| 9e. Reminded you to give your child medication? |  |  |  |  |
| 9f. Reminded you to take your medication? |  |  |  |  |
| 9g. Reminded you of the importance of following doctor’s instructions? |  |  |  |  |
| 9h. Provided money to purchase medication or vitamins? |  |  |  |  |
| 9i. Provide encouragement to live a healthy life? |  |  |  |  |
| 9j. Pray together for the health of your family? |  |  |  |  |

Depression

Please check the answer that comes closes to how you have felt IN THE PAST 7 DAYS, not just how you feel today.

1. I have looked forward with enjoyment to things
2. As much as I ever did
3. A little less than I used to
4. Much less than I used to
5. Not at all
6. I have blamed myself unnecessarily when things went wrong?
7. Yes, often
8. Yes, some of the time
9. Not very often
10. No, never
11. I have felt scared or panicky for no very good reason
12. Yes, quite a lot
13. Yes sometimes
14. No, not much
15. No, not at all
16. I have been so unhappy that I have been crying
17. Yes, most of the time
18. Yes, quite often
19. Only occasionally
20. No, never
21. The thought of harming myself has occurred to me
22. Yes, often
23. Sometimes
24. Hardly ever
25. Never

Risk of Violence

For each of the following statements (15-18), choose the answer which best indicates how often each has occurred in the past 12 months

|  | Never | Rarely | Sometimes | Frequently |
| --- | --- | --- | --- | --- |
| 1. In the past 12 months, has your partner ever insulted you or made you feel bad about yourself? |  |  |  |  |
| 1. In the past 12 months, has your partner ever hit you, kicked you, dragged you, or beaten you up? |  |  |  |  |
| 1. In the past 12 months, has your partner ever hit, kicked, dragged or beaten your child? |  |  |  |  |
| 1. In the past 12 months, has your partner ever physically forced you to have sexual intercourse when you did not want to? |  |  |  |  |

PMTCT Knowledge

1. With what frequency should HIV+ mothers with undetectable viral loads receive a viral load test while they are breastfeeding their baby?
2. Every 3 months
3. Every 6 months
4. Every 12 months
5. Only after their infant has stopped breastfeeding
6. When should HIV+ mothers stop taking their ART?
7. Not until the infant begins eating solid food, at around 6 months.
8. Not until the infant stops breastfeeding.
9. Not until the infant has been confirmed HIV-negative at 18 months of age
10. Never, the mother should continue lifelong ART for her own health.
11. It is recommended that babies continue being routinely tested for HIV until when:
12. 6 months of age
13. 12 months of age
14. 18 months of age
15. 2 years of age
16. The baby stops breastfeeding

PMTCT Motivation

For each of the following, indicate the extent to which you agree or disagree with the statement.

SD=strongly disagree, D=disagree, N=neither agree nor disagree, A= agree, SA = strongly agree.

|  | SD | D | N | A | SA |
| --- | --- | --- | --- | --- | --- |
| 1. I know I can maintain perfect adherence to my medication while my baby is very young and breastfeeding. |  |  |  |  |  |
| 1. Giving my baby his/her medication is extremely important to his health |  |  |  |  |  |
| 1. Even if s/he does not want to take it, I know that I’ll be able to give every dose of his/her medication |  |  |  |  |  |
| 1. It is extremely important to get routine viral load tests while my baby is still breastfeeding. |  |  |  |  |  |
| 1. Other commitments – such as work, household duties, childcare – may prevent me from getting my baby tested by 6 weeks of age. |  |  |  |  |  |
| 1. Attending all of my child’s appointments is extremely important to me. |  |  |  |  |  |

COVID-related questions

Please indicated the degree to which you expect COVID-19 to impact the following events.

|  | Strongly Disagree | Disagree | Neutral | Agree | Strongly Agree |
| --- | --- | --- | --- | --- | --- |
| 1. I was worried that coming to the hospital for infant testing would expose me and my child to COVID-19 |  |  |  |  |  |
| 1. Challenges with travel to and from the hospital (cost and/or availability of cars) as a result of COVID-19 caused me to delay or miss attending some postpartum appointments |  |  |  |  |  |
| 1. I delayed or missed postpartum appointments because I was feeling unwell and did not want to risk exposing others to illness. |  |  |  |  |  |

Assessing delivery VL as intervention

Please indicate the approximate dates (month and year) that you’ve had a VL sample collected, the results of these tests, and the reason for the test since you became pregnant. Please leave blank for any that were not collected.

1. First test since pregnancy was confirmed
2. **Test one** date _______________________
3. Test one results (select one)
   - 1. Detectable
     2. Undetectable
     3. I got the result but don’t remember what it was
     4. I was never informed of the result
4. Purpose of test
5. Routine VL test
6. Follow up from detectable VL
7. Research
8. I don’t know
9. Second test since pregnancy was confirmed
10. **Test two** date _______________________
11. Test two results (select one)
12. Detectable
13. Undetectable
14. I got the result but don’t remember what it was
15. I was never informed of the result
16. Purpose of test
17. Routine VL test
18. Follow up from detectable VL
19. Research
20. I don’t know
21. Third test since pregnancy was confirmed
22. **Test three** date _______________________
23. Test three results (select one)
24. Detectable
25. Undetectable
26. I got the result but don’t remember what it was
27. I was never informed of the result
28. Purpose of test
29. Routine VL test
30. Follow up from detectable VL
31. Research
32. I don’t know
33. Forth test since pregnancy was confirmed
34. **Test four** date _______________________
35. Test four results (select one)
36. Detectable
37. Undetectable
38. I got the result but don’t remember what it was
39. I was never informed of the result
40. Purpose of test
41. Routine VL test
42. Follow up from detectable VL
43. Research
44. I don’t know

Engagement with HITSystem 2.1 - **INTERVENTION ONLY**

1. Since your child was born, have you received any SMS from the hospital?
2. No, I have not received any SMS.
3. Yes, I have received at least one SMS from the hospital but less than 3
4. Yes, I have received between 3 and 5 SMS
5. Yes, I have received more than 5 SMS
6. Have you received and SMS for any of the following reasons?
7. To check how you are doing…………………………………….Yes / No
8. To inform you of your next appointment date………………… Yes / No
9. To remind you to take ART medications……………………… Yes / No
10. To let you know your child was due for services………………Yes / No
11. Asking you to return to the hospital to be informed of a test result………… Yes / No
12. If yes to any option in number 29, please answer question 30 regarding your reaction to receiving the text messages, otherwise skip to 31.
13. I did not find the SMS helpful……………….……………………………….………..True / False
14. I found the SMS helpful to remember to take medication……………………..…..True / False
15. I was worried that someone would see the SMS and discover my status ……….True / False
16. The SMS helped me feel more connected to the hospital/my care provider…….True / False
17. The SMS helped me remember when my appointments were……………………True / False
18. Someone other than me read one of the SMS and asked me about it……………True / False
19. A family member or friend discovered my HIV status because of the SMS………True / False
20. The SMS gave me important information regarding my or my infant’s care………True / False
21. The SMS motivated me to continue PMTCT and/or EID care………………………True / False
22. Please select a preference for frequency of ART adherence messages going forward:
23. Never, please stop sending me adherence messages
24. Daily
25. Weekly
26. Every two weeks
27. Monthly

**Appendix IID. Facility Assessment Form**

Date of survey_________ Conducted by: ____________

1. Facility Name ___________________________________________________
2. Resource level (district, county, provincial, referral, etc)__________________
3. Does the hospital have an implementing partner?
   1. If yes, who:
      1. Afya+
      2. Walter Reed
      3. PEPFAR
      4. FACES
      5. USAID
      6. Other, please specify___________________________
   2. Please describe the primary contributions of the implementing partner, especially in regards to PMTCT, EID, or HIV services at the hospital ________________________________________ ________________________________________________________________________________________________________________________________________________________
4. Please indicate the number of full time (FT) and part-time (PT) doctors, nurses, mentor mothers, or other **clinical personnel** who work primarily in each of the following departments. If one person works in multiple departments, please only indicate the **primary** place of employment to avoid double-counting personnel.

|  |  | Doctor/Clinical Officer | Nurse | Mentor Mother | Other (specify) |
| --- | --- | --- | --- | --- | --- |
| ANC/PMTCT | FT |  |  |  |  |
|  | PT |  |  |  |  |
| Maternity | FT |  |  |  |  |
|  | PT |  |  |  |  |
| MCH | FT |  |  |  |  |
|  | PT |  |  |  |  |
| CCC | FT |  |  |  |  |
|  | PT |  |  |  |  |

1. How many pharmacists are employed:
   1. Full time _________________
   2. Part time_________________
2. How many lab technicians are employed:
   1. Full time __________________
   2. Part time__________________

Please survey the last full **3 months** of paper-based registers to answer the questions 7-. For example, if this survey is being conducted in June, please survey March, April, and May.

1. How many NEW PMTCT enrollments occurred in each of the previous three months

3 months ago _________ 2 months ago___________1 month ago_______________

1. How many patients total filled or refilled ART prescriptions in the previous 3 months (including male and female infants, children, adolescents and adults)

3 months ago _________ 2 months ago___________1 month ago_______________

1. How many women delivered their babies in the maternity in the previous 3 months?

3 months ago _________ 2 months ago___________1 month ago_______________

1. How many infants were enrolled in EID in the previous three months

3 months ago _________ 2 months ago___________1 month ago_______________

**Appendix IIE. Provider Survey**

**Filled by site coordinator:**

Provider ID number _________________

Hospital __________________________

Date of interview___________________

Provider Information

1. Gender………………………………………Male / female
2. Age: _________________________________
3. Please circle the title which best describes your role at the hospital:
   1. Mentor mother
   2. Nurse
   3. Clinical officer/doctor
   4. Laboratory technician
   5. Data entry personnel
   6. Administrator
   7. Other, please specify__________________
4. What department do you work in, primarily:
   1. ANC/PMTCT
   2. Maternity
   3. MCH
   4. Laboratory
   5. CCC
   6. Rotating departments
   7. Other, please specify_______________________
5. How long have you worked in the healthcare field – at this hospital or others? ______________years

PMTCT/VL Knowledge

1. Which best describes your level of knowledge regarding the most recent national guidelines for PMTCT and VL monitoring?
   1. I am very familiar with these guidelines
   2. I am somewhat familiar with these guidelines
   3. I am somewhat unfamiliar with these guidelines
   4. I am very unfamiliar with these guidelines
2. Identify whether each of the following statements is true or false:
   1. Per Kenya national guidelines, an HIV+ pregnant women newly initiated on treatment should receive her first VL test 3 months after treatment is started…………………….True / False
   2. Per Kenya national guidelines, an HIV+ pregnant woman previously initiated on treatment should receive her first VL test 3 months after pregnancy is confirmed………True / False
   3. Per Kenya national guidelines, routine VL testing during pregnancy and breastfeeding should occur every 3 months…………………………………………………………………True / False
   4. Per Kenya national guidelines, HIV+ pregnant women who have high VL results should receive adherence counseling and then a repeat VL test after 3 months……………….True / False
3. Per Kenya national guidelines, All HIV+ pregnant women should receive a minimum of how many ANC appointments?
   1. 2
   2. 3
   3. 4
   4. 5
4. Per Kenya national guidelines, all HIV-exposed infants should receive their first HIV test at the following age:
   1. Within 2 weeks
   2. 6 weeks
   3. 6 months
   4. 12 months
   5. At cessation of breast feeding

Motivation

Please indicate the degree to which you agree or disagree with each of the following statements.

SD= strongly disagree, D=disagree, N=neither agree nor disagree, A=agree, SA = strongly agree

|  | SD | D | N | A | SA |
| --- | --- | --- | --- | --- | --- |
| 1. I always follow national guidelines regarding PMTCT and VL monitoring for HIV+ pregnant and postpartum women. |  |  |  |  |  |
| 1. Other hospital staff always following national guidelines regarding PMTCT and VL monitoring for HIV+ pregnant women and postpartum mothers. |  |  |  |  |  |
| 1. My colleagues and I are actively trying to improve adherence to all national guidelines at the hospital level. |  |  |  |  |  |
| 1. Following national guidelines for VL monitoring among HIV+ pregnant women/new mothers is extremely important for mom and baby’s well-being |  |  |  |  |  |
| 1. Sometimes, it is OK to use professional judgement to delay a VL test, even when guidelines indicate that one should be provided. |  |  |  |  |  |
| 1. Factors outside of my control make it difficult to provide guideline-adherent PMTCT and VL services to HIV+ pregnant women. |  |  |  |  |  |
| 1. I try to provide guideline-adherent PMTCT services (including ART initiation, VL testing, regimen changes, etc.) because colleagues and hospital administration expect it of me. |  |  |  |  |  |
| 1. Some aspects of the national guidelines are more important to adhere to than others |  |  |  |  |  |
| 1. I rely more often on my professional instinct and/or consultation with colleagues to inform patient care than on guidelines |  |  |  |  |  |
| 1. I believe that PMTCT/EID/VL guidelines were developed based on concrete evidence generated through rigorously conducted scientific studies. |  |  |  |  |  |

For questions 20 –26, indicate the level to which you agree or disagree that each of the following are barriers to providing guideline adherent PMTCT care.

SD= strongly disagree, D=disagree, N=neither agree nor disagree, A=agree, SA = strongly agree

|  |  | SD | D | N | A | SA |
| --- | --- | --- | --- | --- | --- | --- |
|  | Colleagues and hospital administration do not always support the provision of guideline-adherent PMTCT and VL services |  |  |  |  |  |
|  | Provider workload is too high to provide all recommended assessments for PMTCT. |  |  |  |  |  |
|  | Guidelines are unclear and/or too complex to follow |  |  |  |  |  |
|  | Mothers to not always consent and/or adhere to the guideline-adherent services that we provide |  |  |  |  |  |
|  | Stock outs of supplies or drugs affect the provision of PMTCT services |  |  |  |  |  |
|  | Patients are not adhering to PMTCT recommendations because they fear exposure to COVID-19. |  |  |  |  |  |
|  | Hospital level protocol changes in response to COVID-19 (e.g. longer drug dispensing intervals, recommendations to limit patient exposure, etc) prevents me and my colleauges from providing PMTCT care as recommended by the national guidelines |  |  |  |  |  |

1. Please describe any other barriers to providing guideline-adherent PMTCT care__________________________________________________________________________________________________________________________________________________________________________________________________________________________________________________

Interaction with HITSystem 2.1 (Intervention sites & End-of-Study, only)

1. On average, how many hours ***per week*** do you use the HITSystem?
   1. <1 hour
   2. 1-3 hours
   3. 3-5 hours
   4. 5-10 hours
   5. >10 hours
2. On average, how many days ***per week*** do you log in to the HITSystem?
3. 1 days
4. 2 days
5. 3 days
6. 4 days
7. 5+ days
8. In the ***past month***, have you logged into the HITSystem for any of the following reasons?
9. To enter a new patient entry……………………………………..…..…….…………….yes / no
10. To update data on an existing patient entry……………..…………………….………..yes / no
11. To use alerts to identify patients who have missed services………………………….yes / no
12. To check a patient’s test result…………………………………………….……………..yes / no
13. To find patient tracing information…………………………………………..………….. yes / no
14. To access Kenya’s national PMTCT/EID/VL guidelines……………………..………..yes / no
15. To generate a report of review facility-level outcomes and data……………………..yes / no
16. Please rank each of the following HITSystem components by their level of usefulness to you, with 1 being the most useful and 5 being the least useful
    1. Patient tracking ________
    2. Accessing guidelines ________
    3. Generating reports ________
    4. Checking a lab result ________
    5. Locating a patient’s clinical record ________
17. Briefly describe any challenges you experienced implementing HITSystem _______________________ _________________________________________________________________________________________________________________________________________________________________________________________________________________________________________________________

**Appendix IIF. Data Collection Tool For Home-based Sample Collection**

**For all home-visits:**

1. Participant ID _______________
2. Date of visit_________________
3. Person conducting visit ____________
4. Reason for visit
   1. Birth VL test
   2. 6-month VL test
   3. Other, please specify____________
5. How many times have study staff attempted to reach this participant through a home visit?
   1. 1
   2. 2
   3. 3
   4. 4 or more
6. Participant’s status
   1. Home
   2. Not at home
   3. Moved to different location
   4. Deceased
7. Is anybody (other than the participant’s infant) home?
   1. Yes, participant’s partner
   2. Yes, participant’s non-infant child or children
   3. Yes, participant’s other family member (please specify who ___________)
   4. Yes, participant’s neighbor or friend
   5. No, participant is alone
8. If YES (a, b, c, or d) to question 7: Has the participant disclosed her HIV status to all people who are currently at her home?
   1. Yes
   2. No
   3. Unknown

***If anyone is home, other than the participant and her infant and she has not disclosed or her disclosure status is unknown, privacy and confidentiality may be compromised. Reschedule VL and EID test (if applicable) to ensure patient confidentiality. Proceed with “well-baby” visit (Questions 9-14).***

***If no-one is home, other than participant and her infant, OR she has disclosed her HIV status to everyone in the home, please proceed to questions 15 - 20***

**Well Baby Visit (if other person is present/confidentiality is compromised):**

1. Baby’s MUAC ____________cm
2. Baby’s temperature _______________^o^ Celcius
3. How is breastfeeding going?
   1. Good
   2. Fair
   3. Poor
4. Any concerns with breastfeeding? _______________________________________________ ____________________________________________________________________________________________________________________________________________________________
5. Any concerns with baby’s health? ___________________________________________________ ____________________________________________________________________________________________________________________________________________________________
6. Any concerns with your own health? ________________________________________________ ____________________________________________________________________________________________________________________________________________________________

**Home visit for VL collection (if confidentiality is secure)**

1. Was VL sample collected……………………………. Y / N

14a. If no, why not?

- 1. Participant refused
  2. Other, please specify ______________

1. Was the survey completed………………. Y / N
   1. If no, why not?
      1. Participant refused
      2. Other, please specify______________________
2. Where is the participant currently accessing HIV services for herself and her infant?
3. At the study hospital
4. At a different health care facility, please specify which__________________
5. With a traditional healer
6. Disengaged from care
7. Other, please specify ______________________________
8. Has the infant had his/her age-appropriate HIV test sample collected at a health care facility (6 wks or 6 months)?........... Y / N
9. **If yes to #18**, which facility ___________________________
10. **If no to #18**, was an EID sample collected at the home visit? ………. Y / N
    - 1. If no to #20, why not?
         1. Participant refused
         2. Infant was not at home
         3. Other, please specify________________________

Swahili Translations

Kiambatisho IIA. Maswali ya Usajili kwa mgonjwa

***Kujazwa na Muuliza maswali:***

Nambari ya usajili: ______________

Tarehe ya mahojiano__________________________

Majina ya muuliza maswali kwa kifupi (Initials of interviewer) _________________________

Demografia ya mama:

1. Tarehe ya kuzaliwa kwa mama: ________________
2. Nambari ya simu ya mama:____________________
3. Maelezo ya jinsi mama anaweza kufikiwa: ___________________________________________________________ _________________________________________________________________________________
4. Tarehe ya kwanza ya ujisajili katika huduma za PMTCT:__________________
5. Makadirio ya tarehe ya kujifungua:______________________
6. Tarehe uliyo anzishwa huduma za HAART: _______________
7. Kiwango chako cha juu zaidi cha elimu ni?
   1. Sina elimu (no formal education)
   2. Sikumaliza shule ya msingi
   3. Nilihitimu shule ya msingi
   4. Sikumaliza sekondari
   5. Nilihitimu sekondari
   6. Sikumaliza chuo kikuu
   7. Nilihitimu chuo kikuu
8. Ni nani umemfahamisha kuhusu hali yako ya VVU?
   1. Hakuna
   2. Mchumba/Mume
   3. Mmoja au zaidi katika familia
   4. Rafiki/Marafiki
   5. Mzazi/Wazazi
9. Je, wewe hufikiaje simu ya rununu?
   1. Nina yangu mwenyewe
   2. Natumia na mtu anayefahamu hali yangu ya VVU
   3. Natumia na mtu asiye fahamu hali yangu ya VVU
   4. Mimi huomba mtu simu ninapohitaji
   5. Sina njia ya mawasiliano ya simu ya rununu
10. Je, hukuchukua mda gani kufika hospitalini (Dakika)______________
11. Gharama za kwenda hospitalini (kuenda na kurudi, KES)______
12. Je, hali yako ya uhusiano ikoje**?**
    1. Sijaoa
    2. Nimeolewa
    3. Sijaoa lakini niko katika uhusiano
    4. Nimeachana na mchumba/mume (separated)
    5. Nimepewa talaka (Divorced)
    6. Ni Mjane
    7. Ingine, fafanua__________
13. Je, hali yako ya maisha ikoje?
    1. Sina mpenzi kwa hivi
    2. Ninaishi kando na mchumba/mume
    3. Ninaishi na mchumba/mume
14. Una watoto wangapi?
    1. Uliowazaa mwenyewe____________
    2. Waliozaliwa na mwingine ___________
15. Je, umewahi kupata huduma za PMTCT/EID mbeleni? ………Ndio/Hapana

Usaidizi toka kwa mpenzi, na maamuzi kuhusu fedha

1. Je, hali ya VVU ya mchumba wako ikoje?
   1. Ana VVU
   2. Hana VVU
   3. Sijui
   4. Sina mpenzi kwa sasa
2. Je, mpenzi wako alichukulia vipi habari za ujauzito wako?
   1. Alikuwa na raha
   2. Alikua vugu vugu (Neutral)
   3. Hakuwa na raha
   4. Sijui
   5. Sina mpenzi kwa sasa
3. Kwa kawaida, ni nani hufanya uamuzi wa matumizi ya pesa katika boma yako?
4. Mchumba wangu, kila mara
5. Mchumba wangu zaidi kuniliko
6. Sote, kwa usawa
7. Mimi, mara nyingi kuliko mwenzi wangu
8. Mimi, kila mara
9. Sina mpenzi kwa sasa
10. Mapato yako ya wastani katika boma lako kila wiki ni?
11. Chini ya 500 KES
12. 500-750 KES
13. 750-1,000 KES
14. 1,000-2,500 KES
15. Zaidi ya 2,500 KES
16. Nina wasiwasi kuwa sitakuwa na pesa za kutosha kunifikisha hospitalini kwa hudum zangu, au za mtoto wangu
    1. Nakubali kabisa
    2. Nakubali
    3. Sikubali
    4. Sikubali kabisa

Usaidizi wa mchumba/Mpenzi

1. Je, kuna umuhimu kwako kwa mchumba wako kukuunga mkono katika taaratibu zifuatazo?

|  | Sio muhimu kabisa | Sio muhimu sana | Muhimu | Muhimu sana | Sina mchumba/  Haihusiani |
| --- | --- | --- | --- | --- | --- |
| 21a. Kuhudhuria taratibu zako za PMTCT na wewe? |  |  |  |  |  |
| 21b. Kukupa pesa za usafiri kwenda hospitalini? |  |  |  |  |  |
| 21c. Kukupa ushauri wa jinsi ya kuishi kwa afya ukiwa mja mzito? |  |  |  |  |  |
| 21d. Kukusaidia majukumu ya nyumbani ili uweze kupumzika? |  |  |  |  |  |
| 21e. Kuku saidia na majukumu ya nyumba, ili uweze kuhudhuria taratibu zako za PMTCT? |  |  |  |  |  |
| 21f. Kukukumbusha kumeza dawa zako? |  |  |  |  |  |
| 21g. Kukukumbusha umuhimu wa kufuata maagizo ya daktari |  |  |  |  |  |
| 21h. Kutoa pesa za kununua dawa au vitamini? |  |  |  |  |  |
| 21i. Kuhudhuria wakati wa kuzaliwa kwa mtoto? |  |  |  |  |  |

1. Je, ni kwa nanma gani unatarajia mchumba wako kukusaidia wakati wa uja uzito?

|  | Sitarajii | Sina uhakika | Natarajia | Sina mchumba/ Hainihusu |
| --- | --- | --- | --- | --- |
| 22a. Kuhudhuria taratibu zako za PMTCT na wewe? |  |  |  |  |
| 22b. Kukupa pesa za usafiri kwenda hospitalini? |  |  |  |  |
| 22c. Kukupa ushauri wa jinsi ya kudumisha afya ukiwa mja mzito? |  |  |  |  |
| 22d. Kukusaidia na majukumu ya boma ili uweze kupumzika? |  |  |  |  |
| 22e. Kuku saidia na majukumu ya boma ili uweze kuhudhuria taratibu zako PMTCT? |  |  |  |  |
| 22f. Kuku kumbusha kumeza dawa zako? |  |  |  |  |
| 22g. Kukukumbusha umuhimu wa kufuata maagizo ya daktari? |  |  |  |  |
| 22h. Kukupa pesa za kununua dawa au vitamini? |  |  |  |  |
| 22i. Kuhudhuria wakati wa kuzaliwa kwa mtoto? |  |  |  |  |

Mawazo

Tafadhali chagua jibu linalo karibiana na jinsi ulivyokuwa ukihisi wiki moja iliyopita, sio leo tu.

1. Ninatazamia maisha ya furaha
   1. Zaidi ya kawaida
   2. Kidogo, kuliko kawaida
   3. Kidogo sana, kuliko kawaida
   4. La
2. Nime jilaumu bila sababu wakati mambo yameenda vibaya
3. Ndio, mara nyingi
4. Ndio, wakati mwengine
5. La,sio sana
6. La, sijawahi
7. Nimehisi hofu na wasiwasi bila sababu ya maana
8. Ndio, mara nyingi
9. Ndio, wakati mwengine
10. La, sio sana
11. La, sijawahi
12. Nimekuwa bila furaha hadi nimekuwa nikilia
13. Ndio, mara nyingi
14. Ndio, wakati mwengine
15. La, sio sana
16. La, sijawahi
17. Nimekuwa na mawazo ya kujiumiza mwenyewe
    1. Ndio, mara nyingi
18. Ndio, wakati mwengine
19. La, sio sana
20. La, sijawahi

Hathari ya vurugu (Risk of Violence)

Kwa kila maelezo yanayofuata, (28-31) chagua jibu linalo onyesha ni mara ngapi tukio limetokea katika miezi 12 iliyopita

|  | Kamwe | Mara chache | Wakati mwingine | Mara nyingi |
| --- | --- | --- | --- | --- |
| 1. Katika miezi 12 iliyopita, mchumba wako amewahi kuku tukana au kukufanya uhisi vibaya? |  |  |  |  |
| 1. Katika miezi 12 iliyopita, mchumba wako amewahi kukugonga, kukupiga mateke, kukuvuta au kukupiga? |  |  |  |  |
| 1. Katika miezi 12 iliyopita, mchumba wako amewahi kumgonga, kumpiga mateke, kumvuta au kumpiga mtoto wako? |  |  |  |  |
| 1. Katika miezi 12 iliyopita, mchumba wako amewahi kukulazimisha kufanya ngono? |  |  |  |  |

Ufahamu wa PMTCT

Tafadhali chagua jibu linalofaa:

1. Wakati wa uja uzito, ninapaswa kufanyiwa uchunguzi wa kiwango cha virusi? (Viral load?)
   1. La, upimaji wa kiwangi cha virusi hufanywa tu baada ya kujifungua
   2. Kati ya mara moja na mbili
   3. Angalau mara tatu
   4. Sijui
2. Wakati wa uja uzito, inashauriwa kwamba wanawake walio na maambukizo ya VVU wameze dawa za ART wakati hawajihisi vizuri pekee………………………Ukweli/Uwongo
3. Je nimependekezwa kuhudhuria idadi gani ya chini, ya clinic za ANC ?
4. 2
5. 3
6. 4
7. 5

Motisha ya PMTCT na Ufanisi wa Kibinafsi

Tafadhali changua jibu linaloonyesha kiwango ambacho unakubali au kutokubaliana na kila moja ya sentensi zifuatazo..

|  | Sikubaliani Kabisa | Sikubaliani | Sina Maoni | Nakubaliana | Nakubaliana sana |
| --- | --- | --- | --- | --- | --- |
| 1. Ninajua naweza kudumisha uzingatiaji kamili wa dawa zangu wakati wote wa uja uzito, hata kukiiwa na watu wengine. |  |  |  |  |  |
| 1. Ni muhimu sana kwangu, kujifungua mtoto wangu kwenye kituo cha afya, badala ya nyumbani. |  |  |  |  |  |
| 1. Nina wasiwasi kwamba sitaweza kupata usafiri kwenda kwa kituo cha afya wakati wa kujifungua mtoto. |  |  |  |  |  |
| 1. Shughuli zingine – kama kazi, majukumu ya nyumbani, ulezi wa watoto – zinaweza kunizuia kuhudhuria taratibu zote za ANC/PMTCT. |  |  |  |  |  |
| 1. Kuhudhuria taratibu zote ya ANC/PMTCT ni muhimu sana kwangu. |  |  |  |  |  |

|  | Sikubaliani Kabisa | Sikubaliani | Sina Maoni | Nakubaliana | Nakubaliana sana |
| --- | --- | --- | --- | --- | --- |
| 1. Nilichelewesha kuja kupokea huduma za ANC kwa sababu ya wasiwasi unaohusiana na mfiduo wa COVID-19 |  |  |  |  |  |
| 1. Nilichelewesha kutafuta huduma kwa sababu mimi, au mtu katika nyumba yangu alikuwa anajisikia vibaya na sikutaka kuhatarisha wengine kwa sababu ugonjwa |  |  |  |  |  |
| 1. Sitaki kuhudhuria kliniki ya ANC mara kwa mara kwa kwa sababu nina wasiwasi juu ya mfiduo wa COVID-19 |  |  |  |  |  |
| 1. Nina wasiwasi ya kwamba kujifungua hospitalini kunaweza kutuweka hatarini, mimi na mtoto kuambukizwa na COVID-19 |  |  |  |  |  |
| 1. Kusafiri kwenda na kutoka hospitalini imekuwa ngumu zaidi (gharama na / au upatikanaji wa magari) kwa sababu ya COVID-19 |  |  |  |  |  |

Asante kwa wakati wako na ushiriki.

**Kiambatisho IIB: Utafiti wa wiki ya nne baada ya kujifungua (Kituo chenye, na kisichokua na mfumo)**

***Kujazwa na muuliza maswali***

Nambari ya Utafiti (Study ID): ______________

Tarehe ya mahojiano__________________________

Majina ya muulizaji maswali kwa kifupi (Interviewer Initials) _________________________

1. Ulijifungua mtoto wako wapi?
   1. Hospitalini/Kituo cha Afya
      1. Ikiwa ni hivyo, tafadhali eleza ni wapii ___________________
   2. Kwa mkunga
      1. Ikiwa ni hivyo, tafadhali eleza eneo ulilo jifungua _______________
   3. Nyumbani
   4. Kwingine
      1. Tafadhali fafanua_________
2. Uchungu wa kujifungua ulikuchua takriban muda gani kabla ya ujifungue?
   1. < masaa 2
   2. Kati ya masaa 2 – 5
   3. Kati ya masaa 6 -12
   4. > Zaidi ya masaa 12
3. Je, wewe au mtoto wako mchanga, mlipatwa na matatizo yoyote?
   1. Ndio
      1. Tafadhali elezea tatizo lilolo mkumba mama na/au mtoto kando kando: ________________________

_________________________________________________________________

- 1. Hapana

Hali ya Mpenzi, na kujulisha wengine kuhusu hali yako

1. Je, hali yako ya mahusiano ni?,
   1. Sijaoa
   2. Nimeolewa
   3. Sijaoa lakini niko na uhusiano
   4. Nimeachana na mchumba/mume (separated)
   5. Nimepewa talaka (Divorced)
   6. Mjane
   7. Ingine, fafanua__________
2. Je, hali yako kimaisha ni?
   1. Sina mpenzi kwa sasa
   2. Naishi kando na mpenzi
   3. Naishi pamoja na mpenzi
3. Je, mpenzi wako wa sasa ndiye baba wa mtoto wako mchanga?.................Ndio/Hapana
4. Je, ni nani umemfahamisha kuhusu hali yako ya VVU? Chagua majibu yote yanayofaa...
   1. Hakuna
   2. Mpenzi/Mume wangu
   3. Mmoja au Zaidi katika familia
   4. Rafiki mmoja, au zaidi
   5. Mzazi mmoja, au zaidi
5. Je, hali ya virusi ya mchumba wako ikoje?
   1. Yuko na VVU
   2. Hana VVU
   3. Sijui
   4. Sina mchumba kwa sasa
6. Kwa kawaida, ni nani hufanya uamuzi kuhusu utumizi wa pesa katika boma lako?
7. Mchumba wangu, kila wakati
8. Mchumba wangu zaidi kuniliko
9. Sote, kwa usawa
10. Mimi, mara nyingi kuliko mpenzi wangu
11. Mimi, kila wakati
12. Sina mchumba kwa sasa
13. Mapato ya wastani ya kila wiki katika boma lako ni?
14. Chini ya 500 KES
15. 500-750 KES
16. 750-1,000 KES
17. 1,000-2,500 KES
18. Zaidi ya 2,500 KES
19. Nina wasiwasi kwamba sitakuwa na pesa za kutosha kunifikisha hospitalini kwa huduma zangu au za mtoto wangu
    1. Nakubali kabisa
    2. Nakubali
    3. Sikubali
    4. Sikubali kabisa

Usaidizi kutoka kwa Mpenzi

1. Ni mara ngapi mchumba wako alikuunga mkon, wakati wa uja uzito, kwa njia zifuatazo,:

|  | Kamwe | Mara chache | Wakati mwingine | Kila Wakati |
| --- | --- | --- | --- | --- |
| 12b. Kukupa pesa za usafiri kwenda hospitalini |  |  |  |  |
| 12c. Kukupa ushauri wa jinsi ya kuishi na afya ukiwa mja mzito |  |  |  |  |
| 12d. Kukusaidia na majukumu ya nyumba, ili upate kupumzika |  |  |  |  |
| 12e. Kukusaidia na majukumu ya nyumba ili uweze kuhudhuria taratibu za PMTCT |  |  |  |  |
| 12f. Kuk kumbusha kumeza dawa |  |  |  |  |
| 12g. Kuku kumbusha umuhimu wa kufuata maagizo ya daktari |  |  |  |  |
| 12h. Kutoa pesa za kununua dawa au vitamini |  |  |  |  |
| 12i. Mume wangu alinitia moyo mda wote wa ujaizito |  |  |  |  |
| 12j. Mimi na mume wangu tuliomba pamoja kwa usala wa mimba na kujifungua. |  |  |  |  |

1. Je, mpenzi wako alihudhuria cliniki yoyote ya PMTCT na wewe?
   1. Hapana, hakuhudhuria yoyote
   2. Alihudhuria moja
   3. Alihudhuria mbili
   4. Alihudhuria tatu au zaidi
   5. Sina mchumba kwa sasa.
2. Ikiwa ni la kwa (13),ni sababu gani ilimfanya ashindwe kuhudhuria?
   1. Alikuwa kazini wakati wa cliniki zangu za PMTCT_____Ndio / Hapana
   2. Anaishi au anafanya kazi mbali ____________________Ndio/Hapana
   3. Alihisi kutokaribishwa au kuto jihisi huru kliniki_________Ndio/Hapana
   4. Sikutaka ahudhurie ___________________________ Ndio/Hapana
   5. Hakutaka kuhudhuria _____________________________ Ndio/Hapana
   6. Alikuwa na majukumu mengine __________________________ Ndio/Hapana
3. Je, mpenzi wako aliweza kukupa usaidizi wakati wa kujifungua? Tafadhali chagua jibu lililo sahihi zaidi.
   1. Sina mpenzi wa kwa sasa
   2. Hapana, mpenzi wangu hakutoa usaidizi wowote kwa wakati wa kujifungua kwangu
   3. Mpenzi wangu alinipa alinifadhili ili nifike hospitali, lakini hakuja.
   4. Mpenzi wangu alinisindikiza hadi hospitali nilipokuwa na maumivu ya kujifungua
   5. Mpenzi wangu alinitembelea hospitali baada ya kujifungua.
   6. Mwenzi wangu wa kiume alinipeleka hospitalini wakati maumivu ya kujifungua yalipoanza, na akanitembelea baada ya kijifungua.

Mawazo

Tafadhali chagua jibu linalo karibiana na ulivyo kuwa ukihisi wiki moja iliyopita, sio leo tu..

1. Ninekua na mtazamo wa maisha kwa furaha
   1. Sana, kuliko kawaida yangu
   2. Kwa kiasi, kuliko kawaida yangu
   3. Kidogo sana, kuliko kawaida yangu
   4. La,
2. Nimejilaumu bila sababu wakati mambo yameenda vibaya
3. Ndio, sana
4. Ndio, mara zingine
5. Sio mara nyingi sana
6. La,
7. Nimehisi hofu na wasiwasi bila sababu ya maana
8. Ndio, mara nyingi
9. Ndio, sio sana
10. La, sio sana
11. La, hasha
12. Nimekuwa bila raha sana hadi nimekuwa nikilia
13. Ndio, mara nyingi
14. Ndio, sio sana
15. La, sio sana
16. La, hasha
17. Nimekuwa na mawazo ya kujiumiza mwenyewe
    1. Ndio, mara nyingi
18. Ndio, sio sana
19. La, sio sana
20. La, hasha.

Hathari ya vurugu (Risk of Violence)

Kwa kila taarifa ifuatayo, (21-24) chagua jibu linaonyesha ni mara ngapi kila tukio limetokea katika miezi 12 iliyopita

|  | La | Mara chache | Wakati mwingine | Mara nyingi |
| --- | --- | --- | --- | --- |
| 1. Katika miezi 12 iliyopita, mchumba wako amewahi kukutukana au kukufanya uhisi vibaya? |  |  |  |  |
| 1. Katika miezi 12 iliyopita, mchumba wako amewahi kukugonga, kukupiga mateke, kukuvuta au kukupiga? |  |  |  |  |
| 1. Katika miezi 12 iliyopita, mchumba wako amewahi kumgonga, kumpiga mateke, kumvuta au kumpiga mtoto wako? |  |  |  |  |
| 1. Katika miezi 12 iliyopita, mchumba wako amewahi kukulazimisha kufanya ngono wakati wewe haujiskii? |  |  |  |  |

Ufahamu wa PMTCT

1. Je, mama mjamzito anaweza kuzuia maambukizi ya VVU kwa mtoto wake kwa njia zifuatazo?

a. Kumeza dawa za ART ipasavyo wakati wa ujauzito na wakunyonyesha.........Ndio /Hapana/Sijui

b. Kujifungulia kwenye kituo cha afya, badala ya nyumbani…………............Ndio /Hapana/Sijui

c. Kutumia lishe ya fomula kwa watoto akiwa na uwezo/ kunyonyesha mtoto asipo kua na uwezo haipo………… Ndio /Hapana/Sijui

d. Kumpa mtoto Nevirapine…………………………................................................. Ndio /Hapana/Sijui

e. Kumpa mtoto Cotrimoxazole (Septrin)…………………………………………………..……...Ndio /Hapana/Sijui

e. Kumpa maziwa ya ng’ombe na mayai kabla ya umri wa miezi 6 kumsaidia kupata nguvu… Ndio /Hapana/Sijui

1. Inapendekezwa kuwa mtoto wangu apate kipimo cha VVU cha kwanza?
   1. Ndani ya wiki mbili baada ya kuzaliwa
   2. Akiwa na umri wa wiki 6
   3. Akiwa na umri wa miezi 6
   4. Akiwa na umri wa miezi 12
   5. Mara tu akiacha kunyonya
   6. Sijui
2. Inapendekezwa kuwa watoto wanaendelea kupimwa VVU mara, kwa mara hadi:
   1. Akiwa na miezi 6
   2. Akiwa na miezi 12
   3. Akiwa na miezi 18
   4. Akiwa na miaka 2
   5. Mara tu akiacha kunyonya

Motisha kuhusu taratibu za PMTCT

Tafadhali changua jibu linaloonyesha kiwango ambacho unakubali au haukubaliani na kila mmoja ya sentensi zifuatazo..

|  | Sikubaliani Kabisa | Sikubaliani | Sina Maoni | Nakubaliana | Nakubaliana kabisa |
| --- | --- | --- | --- | --- | --- |
| 1. Ninajua naweza kuzingatia umezaji dawa zangu kikamilifiu wakati mtoto wangu ni mchanga na ninamnyonyesha |  |  |  |  |  |
| 1. Kumpa mtoto wangu dawa yake ni muhimu sana, kwa afya yake |  |  |  |  |  |
| 1. Hata ikiwa hataki kumeza, najua kwamba nitaweza kumpa dawa yake kulingana na kipimo. |  |  |  |  |  |
| 1. Ni muhimu sana kufanyiwa uchunguzi wa kawaidi wa kiwango cha Virusi mwilini wakati bado mtoto wangu ananyonya. |  |  |  |  |  |
| 1. Shughuli zingine – Kama kazi, majukumu ya nyumbani, ulezi wa watoto – yanaweza kunizuia kumpima mtoto wangu akifikishapo umri wa wiki 6 |  |  |  |  |  |
| 1. Kuhudhuria kliniki zote za mtoto wangu ni muhimu sana kwangu |  |  |  |  |  |

|  | Sikubaliani Kabisa | Sikubaliani | Sina Maoni | Nakubaliana | Nakubaliana kabisa |
| --- | --- | --- | --- | --- | --- |
| 1. Nilikuwa na wasiwasi ya kwamba kujifungua hospitalini kunaweza kutuweka hatarini, mimi na mtoto kuambukizwa na COVID-19 |  |  |  |  |  |
| 1. Nina wasiwasi ya kwamba kuenda hospitalini kwa ajili ya upimaji wa watoto inaweza kutuweka hatarini, mimi na mtoto kuambukizwa na COVID-19 |  |  |  |  |  |
| 1. Wakati wa ujauzito, changamoto za kusafiri kwenda na kutoka hospitalini (gharama na / au upatikanaji wa magari) kwa sababu ya COVID-19 ilinifanya kuchelewesha au kukosa kuhudhuria miadi ya ANC au kujaza dawa. |  |  |  |  |  |
| 1. Wakati wa ujauzito, nilichelewesha kutafuta huduma kwa sababu mimi, au mtu katika nyumba yangu alikuwa anajisikia vibaya na sikutaka kuhatarisha wengine kwa sababu ugonjwa |  |  |  |  |  |

MAWASILIANO NA HITSYSTEM – **KITUO KITAKACHO TUMIA MFUMO PEKEE**

1. Je, umepokea SMS yoyote toka hospitali, tangu ujiandikishe kwa taratibu ya ANC,?
   1. Hapana, sijapata SMS yoyote
   2. Ndio, nimepokea SMS kama moja hivi, lakini sio zaidi ya 3
   3. Ndio, nimepokea kati ya SMS 3 -5
   4. Ndio, nimepokea zaidi ya SMS 5
2. Je, umepokea SMS yoyote kwa sababu zifuatazo:
3. Kukujulia unavyo endelea na hali…………………………………….Ndio/Hapana
4. Kukujulisha tarehe ijayo ya cliniki………………………….Ndio/Hapana
5. Ili kukukumbusha kumeza dawa zako za ART…………...Ndio/Hapana
6. Kukujulisha kwamba mtoto anapaswa kuja kupokea huduma………Ndio/Hapana
7. Kukuuliza urudi hospitali kupokea matokeo ya kipimo………… Ndio/Hapana
8. Kama umejibu ndio, kwa chaguo lolote swali nambari 34, tafadhali jibu swali 36, kuhusu majibu yako ya kupokea ujumbe wa maandishi, Ikiwa ni la, ruka hadi 37.
9. Sikuona SMS ikinisaidia……………….……………………………….………..Ndio/Hapana
10. Nilipata SMS ikinisaidia kuni kumbusha kumeza dawa……………………..…..Ndio/Hapana
11. Nilikuwa na wasiwasi kwamba mtu angeona SMS na kugundua hali yangu ……….Ndio/Hapana
12. SMS ilinisaidia kuhisi kushikamana zaidi na hospitali/mhudumu wangu…….Ndio/Hapana
13. SMS ilinisaidia kukumbuka siku zangu za kliniki……………………Ndio/Hapana
14. Mtu mwingine alisoma moja ya SMS na kuniuliza juu yake……Ndio/Hapana
15. Mtu katika familia au rafiki, aligundua hali yangu ya VVU kutokana na SMS…Ndio/Hapana
16. SMS ilinipa habari muhimu kuhusu afya yangu, au ya mtoto wangu………Ndio/Hapana
17. SMS ilinipa moyo kuendeleza taratibu za PMTCTna/au EID……………………Ndio/Hapana
18. Tafadhali chagua mapendekezo ya ujumiwaji jumbe za uzingativu wa ART kuendelea mbele:
19. La, tafadhali acheni kutuma jumbe za uzingativu
20. Kila siku
21. Kila wiki
22. Kila wiki mbili
23. Kila mwezi

Asante kwa wakati wako na kwa kushiriki.

**Kiambatisho IIC. Maswali Miezi 6 Baada ya Kujifungua (Mahali mfumo utatumika, na ambapo hautatumika)**

***Kujazwa na muuliza maswali***

Nambari ya usajili (Study ID): ______________

Tarehe ya mahojiano__________________________

Jina la muuliza maswali kwa ufupi (Interviewer Initials) _________________________

**Hali ya Mshirika na Udhihirisho**

1. Hali yako ya uhusiano ni gani?
   1. Sijaoa
   2. Nimeolewa
   3. Sijaoa lakini katika uhusiano
   4. Nimeachana na mchumba/mume (separated)
   5. Nimepewa talaka (Divorced)
   6. Mjane
   7. Ingine, fafanua__________
2. Hali yako ya kuishi?
   1. Sina mwenzi wa uhusiano kwa sasa
   2. Ninaishi mbali na mpenzi wangu
   3. Naishi pamoja na mpenzi wangu wa sasa.
3. Je, umemfahamisha nani kuhusu hali yako ya VVU? Chagua majibu yote yanayofaa...
   1. Hakuna
   2. Mshiriki/Mchumba/Mume
   3. Mmoja au zaidi katika familia
   4. Rafiki/Marafiki
   5. Mzazi/Wazazi
4. Je, hali ya virusi ya mpenzi wako ikoje?
   1. Yuko na VVU
   2. Hana VVU
   3. Sijui
   4. Sina mpenzi kwa sasa
5. Je, mpenzi wako wa sasa, ndiye baba wa mtoto wako mchanga?...........Ndio/La
6. Kwa kawaida, ni nani hufanya uamuzi kuhusu utumizi wa pesa katika boma lako?
7. Mchumba wangu- kila wakati
8. Mchumba wangu- zaidi kuniliko
9. Sote, kwa usawa
10. Mimi, mara nyingi kuliko mpenzi wangu
11. Mimi, kila mara
12. Sina mchumba kwa sasa
13. Mapato ya wastani kila wiki, katika boma lako ni?
14. Chini ya 500 KES
15. 500-750 KES
16. 750-1,000 KES
17. 1,000-2,500 KES
18. Zaidi ya 2,500 KES
19. Nina wasiwasi kwamba sitakuwa na pesa za kutosha kunifikisha hospitalini kwa huduma yangu, au ya mtoto wangu.
    1. Nakubaliana kabisa
    2. Nakubali
    3. Sikubali
    4. Sikubaliani kabisa

Usaidizi kutoka kwa Mpenzi (Kujazwa wakati wa upimaji kiwango cha virusi wa Miezi 6)

1. Ni kwa kiwango gani mpenzi wako amekusaidia toka ujifungue, kwa njia zifuatazo:

|  | Kamwe | Mara chache | Wakati mwingine | Kila wakati |
| --- | --- | --- | --- | --- |
| 9a. Kuhudhuria miadi ya matibabu ya mtoto wako |  |  |  |  |
| 9b. Kutoa pesa za usafiri kwenda hospitalini |  |  |  |  |
| 9c. Kukupa ushauri kuhusu lishe na usafi wa mtoto |  |  |  |  |
| 9d. Kukusaidia na majukumu ya nyumba ili uweze kupumzika |  |  |  |  |
| 9e. Kunikumbusha kumpa mtoto dawa. |  |  |  |  |
| 9f. Kuinikumbusha kumeza dawa zangu. |  |  |  |  |
| 9g. Kunikumbusha kuhusu umuhimu wa kufuata maagizo ya daktari. |  |  |  |  |
| 9h. Kutoa pesa za kununua dawa au vitamini |  |  |  |  |
| 9i. Kunitia moyo wa kuishi maisha bora |  |  |  |  |
| 9j. Tulioomba pamoja kuhusu afya ya familia yetu. |  |  |  |  |

Hofu

Tafadhali chagua jibu linalo karibiana na jinsi ulivyo kuwa unahisi wiki moja iliyopita, sio leo tu

1. Nina mtazamo wa maisha yenye furaha
   1. Sana, kulpita kawaida
   2. Kidogo, kuliko kawaida
   3. Kidogo sana, kuliko kawaida
   4. La, hasha.
2. Nimejilaumu bila sababu wakati mambo yameenda vibaya
   1. Ndio, mara nyingi
   2. Ndio, wakati mwengine
   3. Sio mara nyingi sana
   4. La, hasha.
3. Nimehisi hofu/ wasiwasi bila sababu ya maana
   1. Ndio, mara nyingi
   2. Ndio, mara kadhaa
   3. Mara chache
   4. La, hasha.
4. Nimekuwa niki huzunika hadi kulia
   1. Ndio, mara nyingi
   2. Ndio, mara kadhaa
   3. Mara chache
   4. La, hasha.
5. Nimekuwa nikijiwa na mawazo ya kujiumiza mwenyewe
   1. Ndio, mara nyingi
   2. Ndio, mara kadhaa
   3. Mara chache sana
   4. La, hasha.

Athari ya vurugu (Risk of Violence)

Kwa kila taarifa ifuatayo, (15-18) chagua jibu linaonyesha kwa ubora, ni mara ngapi tukio lilitokea katika miezi 12 iliyopita

|  | Kamwe | Mara chache | Wakati mwingine | Mara nyingi |
| --- | --- | --- | --- | --- |
| 1. Katika miezi 12 iliyopita, mchumba wako amewahi kuku tukana au kukufanya uhisi vibaya? |  |  |  |  |
| 1. Katika miezi 12 iliyopita, mchumba wako amewahi kukugonga, kukupiga mateke, kukuvuta au kukupiga? |  |  |  |  |
| 1. Katika miezi 12 iliyopita, mchumba wako amewahi kumgonga, kumpiga mateke, kumvuta au kumpiga mtoto wako? |  |  |  |  |
| 1. Katika miezi 12 iliyopita, mchumba wako amewahi kukulazimisha kufanya ngono wakati wewe haukutaka? |  |  |  |  |

Ufahamu wa taratibu za kumkinga mtoto dhidi ya maambukizo ya virusi kutoka kwa mama (PMTCT)

1. Ni kwa mda ngani, akina mama wanaonyonyesha, na wenye viwango vya Vurusi visivyo tambulika, hufaa kupimwa tena?
   1. Kila miezi 3
   2. Kila miezi 6
   3. Kila miezi 12
   4. Mara tu mtoto aachapo kunyonya
2. Je, ni wakati gani mama aliye na VVU hupendekezwa aache kumeza dawa za VIrusi?
   1. Hadi mtoto atakapoanza kula chakula kigumu, karibu miezi 6
   2. Hadi mtoto aache kunyonya
   3. Hadi mtoto atakapothibitishwa kuwa hana VVU akiwa umri wa miezi 18
   4. La, mama anapaswa kuendelea na Maisha ya kumeza dawa za ART kwa afya yake mwenyewe.
3. Ki utaratibu, inapendekezwa watoto waendelee kufanyiwa uchunguzi wa VVU hadi?
   1. Umri wa miezi 6
   2. Umri wa miezi 12
   3. Umri wa miezi 18
   4. Umri wa miaka 2
   5. Mara tu aachapo kunyonya

Motisha wa uzungativu kwa taratibu za Kumkinga mtoto dhidi ya maambukizo toka kwa mama (PMTCT)

Tafadhali changua jibu linaloonyesha kiwango ambacho unakubaliana au kutokubaliana na kila sentensi zifuatazo..

|  | Sikubaliani Kabisa | Sikubaliani | Sina Maoni | Nakubaliana | Nakubaliana kabisa |
| --- | --- | --- | --- | --- | --- |
| 1. Najua naweza kudumisha uzingativu wa umesaji dawa ipasavyo, wakati mtoto wangu ni mchanga na ananyonya |  |  |  |  |  |
| 1. Kumpa mtoto wangu dawa yake ni muhimu sana kwa afya yake |  |  |  |  |  |
| 1. Hata ikiwa haipendi, najua kuwa nitaweza kumpa kulingana na kipimo cha dawa yake |  |  |  |  |  |
| 1. Ni muhimu sana kupata vipimo vya upimaji wa kiwango cha virusi kitaratibu, wakati mtoto wangu bado ananyonya. |  |  |  |  |  |
| 1. Majukumu mengine – Kama kazi, shughuli za nyumbani, kushughulikia watoto – zinaweza kunizuia kumfanyia mwanangu upimaji, afikishapo wiki 6. |  |  |  |  |  |
| 1. Kuhudhuria taratibu zote za cliniki za mtoto wangu ni muhimu sana kwangu |  |  |  |  |  |

|  | Sikubaliani Kabisa | Sikubaliani | Sina Maoni | Nakubaliana | Nakubaliana kabisa |
| --- | --- | --- | --- | --- | --- |
| 1. Nilikuwa na wasiwasi kuwa kuja hospitalini kwa upimaji wa watoto kunaweza kutuweka hatarini, mimi na mtoto kuambukizwa na COVID-19 |  |  |  |  |  |
| 1. Changamoto za kusafiri kwenda na kutoka hospitalini (gharama na / au upatikanaji wa magari) kwa sababu ya COVID-19 ilinifanya kuchelewesha au kukosa kuhudhuria miadi ya baada ya kujifungua. |  |  |  |  |  |
| 1. Nilichelewesha au nilikosa miadi ya baada ya kujifungua kwa sababu nilikuwa najisikia vibaya na sikutaka kuhatarisha wengine kwa ugonjwa. |  |  |  |  |  |

Kutathmini uchunguzi wa kiwango cha VVU baada ya kijifungua, kama mfumo wa taratibu ya utafiti

Tafadhali fanya makadirio ya tarehe (Mwezi/Mwaka) ambapo ulitolewa sampuli kuchunguzwa kiwango cha virusi, matokeo ya uchunguzi huu, na sababu ya uchunguzi toka uwe mja zito. Tafadhali acha pengo, kwa yoyote ambayo haiku tolewa.

1. Kipimo cha kwanza tangu uthibitishwe kuwa na uja uzito
   1. **Kipimo cha kwanza:** Tarehe _______________________
   2. Matokeo ya kipimo cha kwanza (chagua moja)
      1. Kiwango kinachoonekana
      2. Kiwango kisichoonekana
      3. Nilipata matokeo lakini sikumbuki majibu
      4. Sikuwahi kufahamishwa matokeo
   3. Lengo la Kipimo:
      1. Ujinguzi wa kawaida
      2. Uchunguzi kufuatilia kiwango cha virusi kilichoonekana hapo awali
      3. Uchunguzi kwa Utafiti
      4. Sijui
2. Kipimo cha pili tangu uthibitishwe kuwa mja mzito
   1. **Kipimo cha pili:** Tarehe _______________________
   2. Matokeo ya kipimo cha pili (chagua moja)
      1. Kiwango kinachoonekana
      2. Kiwango kisichoonekana
      3. Nilipata matokeo lakini sikumbuki majibu
      4. Sikuwahi kufahamishwa matokeo
   3. Lengo la Kipimo:
      1. Uchunguzi wa kawaida
      2. Uchunguzi kufuatilia kiwango cha virusi kilichoonekana hapo awali
      3. Uchunguzi kwa Utafiti
      4. Sijui
3. Kipimo cha tatu tangu uthibitishwe kuwa na ujauzito
   1. **Kipimo cha tatu:** Tarehe _______________________
   2. Matokeo ya kipimo cha tatu (chagua moja)
      1. Kiwango kinachoonekana
      2. Kiwango kisichoonekana
      3. Nilipata matokeo lakini sikumbuki majibu
      4. Sikuwahi kufahamiswa matokeo
   3. Lengo la Kipimo:
      1. Uchunguzi wa kawaida
      2. Uchunguzi kufuatilia kiwango cha virusi kilichoonekana hapo awali
      3. Uchunguzi kwa Utafiti
      4. Sijui
4. Kipimo cha nne tangu uthibitishwe kuwa na ujauzito
   1. **Kipimo cha nne:** Tarehe _______________________
   2. Matokeo ya kipimo cha nne (chagua moja)
      1. Kiwango kinachoonekana
      2. Kiwango kisichoonekana
      3. Nilipata matokeo lakini sikumbuki matokeo
      4. Sikuwahi kuambiwa matokeo
   3. Lengo la Kipimo cha kiwango cha virusi:
      1. Uchunguzi wa kawaida
      2. Uchunguzi kufuatilia kiwango cha virusi kilichoonekana hapo awali
      3. Uchunguzi kwa Utafiti
      4. Sijui

MAWASILIANO NA HITSYSTEM 2.0 – KITUO KITAKACHO TUMIA MFUMO PEKEE

1. Tangu mtoto wako azaliwe, umepokea SMS yoyote kutoka hospitalini?
   1. Hapana, sijapata SMS yoyote
   2. Ndio, nimepokea SMS moja ila hazijazidi 3
   3. Ndio, nimepokea kati ya SMS 3 -5
   4. Ndio, nimepokea Zaidi ya SMS 5
2. Je, umepokea SMS yoyote kwa sababu zifuatazo:
   1. Kukujulia hali…………………………………….Ndio/Hapana
   2. Kukufahamisha tarehe zako za kiliki………………………….Ndio/Hapana
   3. Kukukumbusha kumeza dawa zako za Virusi…………...Ndio/Hapana
   4. Kuku kumbusha tarehe za huduma za mtoto zinapo wadia………Ndio/Hapana
   5. Kukuuliza urudi hospitalini kufahamishwa matokeo ya vipimo………… Ndio/Hapana
3. Kama umejibu ndio, kwa chaguo lolote la nambari 32, tafadhali jibu swali 34 kuhusu majibu yako ya kupokea ujumbe wa maandishi, ikiwa ni la, ruka hadi 35.
4. Sikuona SMS ikisaidia……………….……………………………….………..Ndio/Hapana
5. Nilipata SMS ikinisaidia kunikumbusha kuchumeza dawa……………………..…..Ndio/Hapana
6. Nilikuwa na wasiwasi kuwa mtu angeona SMS na kuwa na shauku ya hali yangu ……….Ndio/Hapana
7. SMS ilinisaidia kujihisi kushikamana zaidi na hospitali/Mhudumu wangu…….Ndio/Hapana
8. SMS ilinisaidia kukumbuka tarehe za cliniki zilipofika……………………Ndio/Hapana
9. Mtu mwingine aliwa kusoma moja ya SMS na akaniuliza juu yake……Ndio/Hapana
10. Mtu wa familia au rafiki yangu, aligundua hali yangu ya VVU kutokana na SMS…Ndio/Hapana
11. SMS ilinipa habari muhimu kuhusu utunzaji wangu au wa mtoto wangu………Ndio/Hapana
12. SMS ilinipa moyo kuendelea na huduma ya PMTCT/au EID……………………Ndio/Hapana
13. Tafadhali chagua mapendekezo ya utumiwaji jumbe za uzingatiifu wa ART kuendelea mbele:
14. La, tafadhali acheni kutuma jumbe za uzingativu
15. Kila siku
16. Kila wiki
17. Kila wiki mbili
18. Kila mwezi

Asante kwa wakati wako, na kwa kushiriki.

**Kiambatisho F. Kijalada chana ukusanyaji taarifa kwa sampuli zitazo tolewa Nyumbani**

**Kwa matembezi yote ya nyumbani:**

1. Nambari ya mshiriki _______________
2. Tarehe ya kutembelewa_________________
3. Anaye mtembelea____________
4. Sababu ya kumtembelea:
   1. Kipimo cha kiwango cha virusi – Wakati wa kujifungua
   2. Kipimo cha kiwango cha virusi - Miezi 6
   3. Sababu nyingine, tafadhali eleza____________
5. Je, ni mara ngapi, wafanyikazi wa utafiti, wamejaribu kumfikia mshiriki kupitia ziara ya nyumbani?
   1. 1
   2. 2
   3. 3
   4. 4 au Zaidi
6. Hali ya mshiriki:
   1. Yupo nyumbani
   2. Hayupo nyumbani
   3. Amehama eneo tofauti
   4. Amefariki/Marehemu
7. Kuna mtu yeyote (mbali na mtoto wa mshiriki) nyumbani?
   1. Ndio, mpenzi wa mshiriki
   2. Ndio, mtoto/Watoto wa mshiriki wasio wachanga
   3. Ndio, mtu mwingine katika familia ya mshiriki (tafadhali taja nani ___________)
   4. Ndio, jirani au rafiki ya mshiriki
   5. Hapana, mshiriki yuko peke yake
8. Kama ni ndio kwa (a, b, c, ama d) katika swali nambari 7: Je, mshiriki amefichua hali yake ya VVU kwa watu wote walio nyumbani kwake kwa sasa?
   1. Ndio
   2. Hapana
   3. Haijulikani

***Ikiwa yuko mtu mwengine nyumbani, mbali na mshiriki na mtoto wake, na mshiriki hajafichua hali yake au hali yake haijulikani, usiri unaweza kuathirika. Tafadhali badili ratiba ya upimaji kiwango cha virusi (VL), au Upimaji wa mtoto (EID) ili kuhakikisha usiri kwa mshiriki umehifadhiwa. Endelea na maswali ya kwaaidi ya ziara ya kujilia mtoto hali (Maswali 9 – 14).***

***Ikiwa hakuna mtu mwengine nyumbani, mbali na mshiriki na mtoto wake, AU, Iwapo mshiriki amefichua hali yake ya VVU kwa kila mtu anayeishi nyumbani, tafadhali endelea na maswali 15 – 20.***

**Matembezi kujulia hali Mtoto Mchanga (Ikiwa mtu mwingine yupo / usiri uta tatatizwa):**

1. Kipimo cha msuli wa mkono(MUAC) kwa mtoto ____________(sentimita)
2. Joto ya mtoto _______________^o^ C (Digrii celicius)
3. Kunyonya kuna mwendeaje?
   1. Vizuri
   2. Sawa
   3. Vibaya
4. Una wasiwasi wowote kuhusu kunyonyesha? _______________________________________________ ____________________________________________________________________________________________________________________________________________________________
5. Una wasiwasi wowote kuhusu afya ya mtoto? ___________________________________________________ ____________________________________________________________________________________________________________________________________________________________
6. Una wasiwasi wowote kuhusu afya yako? ________________________________________________ ____________________________________________________________________________________________________________________________________________________________

**Matembezi nyumbani-Kutoa sampuli ya uchunguzi wa kiwango cha virusi (endapo kuna hakikisho la usiri)**

1. Je, sampuli ya kuchunguza kiwango cha virusi ilitolewa?……………………………. Ndio / La

14a. Ikiwa La, ni kwanini?

- 1. Mhusika alikataa
  2. Zenginezo, tafadhali eleza ______________

1. Je, Maswali yamekamilishwa?………………. Ndio / La
   1. Ikiwa La, ni kwa nini?
      1. Mhusika alikataa
      2. Zenginezo, tafadhali eleza______________________
2. Je, ni wapi mhusika anaendeleza huduma zake na za mwanawe, za matibabu ya virusi?
3. Katika hospital kunapo endelezwa utafiti
4. Katika hospitali taofauti, tafadhali elezea ni gani?__________________
5. Kwa mganga wa kienyeji
6. Aliacha taratibu
7. Zenginezeo, tafadhali eleza ______________________________
8. Je, mtoto ameweza kutolewa sampuli za upimaji virusi, kulingana na umri (Wiki 6, au Miezi 6), katika kituo?...................Ndio/La.
9. **Ikiwa ni Ndio, kwa #18**, ni kituo gani? ___________________________
10. **Ikiwa ni La, kwa #18**, Je, sampuli ya EID ilitolewa alipotembelewa nyumbani? ………. Ndio / La
    - 1. Ikiwa ni La, kwa #20, Ni kwanini?
         1. Mhusika alikataa
         2. Mtoto hakuweko nyumbani
         3. Zenginezo, tafadhali eleza________________________

Luo Translations

**Appendix IIA. Enrollment Survey**

***For interviewer to fill out:***

Study ID: ______________

Date of interview__________________________

Interviewer initials_________________________

Mother Demographics

1. Ne onyuoli karango: ________________
2. Namba ni mar simu:____________________
3. Yo ma inyalo yudi godo mayot: _______________________________________________________ _________________________________________________________________________________
4. Tarik mane ichako yudo e thieth mar PMTCT:__________________
5. Inyuol tarik adi (EDD) ______________________
6. Tarik mane ichako thieth mar HAART _______________
7. Isomo ma I gik kanye?
   1. Ne ok a somo
   2. Ne ok atieko primary
   3. Ne atieko primary
   4. Ne ok atieko secondary
   5. Ne atieko secondary
   6. Mbalariany/college
8. Chal mari mar ayaki isenyiso ng’a??
   1. Onge ng’ato ma asenyiso
   2. Jaoda/jaherana
   3. Ng’ato achiel kase anyuola
   4. Ng’ato achiel kase osiepni
   5. Ng’ato achiel kose janyuolni
9. Ere kaka I goyo simu?
   1. An kod simu mara
   2. Wariwo kod ng’at ma ongeyo chal mara mar ayaki
   3. Wariwo kod ng’at ma ok ongeyo chal mara mar ayaki
   4. Akwayo simu seche ma adwaro
   5. Onge kaka dayud simu
10. Dhi hospital kawi saa marom nade (minutes)______________
11. Itiyo kod pesa adi dhi hospital, dhi gi duogo (roundtrip, KES)______
12. Kit keny mari chal nade**?**
    1. Pok adhi katedo
    2. Ose kenda
    3. Pok okenda to an an kod jaherana
    4. Wapogore kod jaherana
    5. Wawere kod jaherana
    6. An Chi liel
    7. Mamoko, ler ane__________
13. Dak mari chal nade?
    1. Aonge jaherana sani
    2. Adak mopogore kod jaherana
    3. Adak kamoro achiel kod jaherana
14. Nyithindi adi ma ngima?
    1. Ma Inyuolo____________
    2. Ma ok inyuolo___________
15. Bende ne iseyudo thieth mar PMTCT kata EID chon matin? ………Y / N

Partner support & Financial Control

1. Chal mar ayaki mar jaherani?
   1. En kod kute mag ayaki
   2. Oonge kod kute mag ayaki
   3. Ok ong’ere
   4. Aonge ng’ato sani
2. Jaherani ne otimre nade kane owinjo ni iyach?
   1. Ne Omor
   2. Ne entie kanyo
   3. Ne ok omor
   4. Ok ang’eyo
   5. Aonge jaherana sani
3. En ng’a ma chiwo yore kaka onego uti kod pesa e odu?
4. Jaherana, seche duto
5. Jaherana ngenyne ma oloya
6. Jaherana kod an maromre
7. An ngenyne moloyo jaherana
8. An, Seche duto.
9. Aonge jaherana sani
10. Yuto maru e juma
11. Tin ne mia 500
12. Kind mia 500-750
13. Kind mia 750-1,000
14. Kind 1,000-2,500
15. Oingo 2,500
16. Chunya chandre nikech aonge pesa moromo ma anyalo chopo godo hospital kata ma nyathina nyalo yudo godo thieth?
    1. Ayiego Ahinya
    2. Ayiego
    3. Adagi
    4. Adagi Ahinya

Partner Support

1. Be ineno ka en gima ber mondo jaherani omiyi sir e yore gi, kaka ?

|  | Ok ochuno ahinya |  | Ok ochuno | Ochuno matin | Ochuno ahinya | Aonge kod jaherana |
| --- | --- | --- | --- | --- | --- | --- |
| 21a Dhi kodi kanyakla achiel e thieth mar PMTCT? |  |  |  |  |  |  |
| 21b. Miyi pesa mar dhi godo hospital? |  |  |  |  |  |  |
| 21c. Miyi paro kaka inyalo bedo gi ngima maber ndalo ma iyach? |  |  |  |  |  |  |
| 21d. Konyi kod tije mag ot mondo iywe? |  |  |  |  |  |  |
| 21e. Konyi kod tije mag ot mondo idhi e thieth mar PMTCT? |  |  |  |  |  |  |
| 21f. Paroni mondo imwony yath? |  |  |  |  |  |  |
| 21g. Paroni mondo iluw thieth kaka daktari ne owacho? |  |  |  |  |  |  |
| 21h. Miyi pesa mar nyiew yath kata vitamins? |  |  |  |  |  |  |
| 21i. Dhi nywol? |  |  |  |  |  |  |
| 21j. Miyi mijing’o |  |  |  |  |  |  |

1. Gin yore mage madiher mondo jaherani osiri godo ndalo ma iyach?

|  | Ne onge geno | Ok ongere | Ne an kod geno | Aonge kod jaherana |
| --- | --- | --- | --- | --- |
| 22a Dhi kodi kanyakla achiel e thieth mar PMTCT? |  |  |  |  |
| 22b Miyi pesa mar dhi godo hospital? |  |  |  |  |
| 22c. Miyi paro kaka inyalo bedo gi ngima maber ndalo ma iyach? |  |  |  |  |
| 22d. Konyi kod tije mag ot mondo iywe? |  |  |  |  |
| 22e. Konyi kod tije mag ot mondo idhi e thieth mar PMTCT? |  |  |  |  |
| 22f. Paroni mondo imwony yath? |  |  |  |  |
| 22g. Paroni mondo iluw thieth kaka daktari ne owacho? |  |  |  |  |
| 22h. Miyi pesa mar nyiew yath kata vitamins? |  |  |  |  |
| 22i Dhi nywol? |  |  |  |  |
| 22j. Miyi mijing’o |  |  |  |  |

Depression

Kiyie to yier dwoko machiegni gi kaka ne iwinjo kuom ndalo 7 mosekalo,ok kawuono kende.

1. Ase ngiyo mbele kod mor mar gik mangeny
2. Kaka asebedo ga katimo
3. Matin kaka asebedo katimo
4. Matin ahinya moloyo kaka asebedo
5. Ak asebedo
6. Weche ka odhi marach to asebedo ka ajilaumu
7. Eeh, Ngenyne
8. Eeh , seche moko
9. Ok seche te
10. Ooyo, podi
11. Asebedo ka awinjo ka aluor ma onge gima omiyo
12. Eeh,seche mangeny
13. Eeh, seche moko
14. Ooyo, ok ahinya
15. Ooyo,podi
16. Asebedo ma ok amor, ma asebedo ka aywak
17. Eeh, seche mangeny
18. Eeh, seche moko
19. Mana seche moko
20. Ooyo,podi
21. Paro mar hinyruok an awuon ase bedogodo
22. Eeh, Ngenyne
23. Seche moko
24. Mana seche moko
25. Podi

Risk of Violence

Kuom weche gi chakre (27-30), yier duoko manyiso kaka gik moko osetimre kuom dweche 12 mosekalo

|  | Podi | Ok ahinya | Seche moko | Ngenyne |
| --- | --- | --- | --- | --- |
| 1. Kuom dweche 12 mosekalo, be jaherani ose yanyi kata miyi mondo iwinj marach ne in iwuon? |  |  |  |  |
| 1. Kuom dweche 12 mosekalo , be jaherani ose goyi, gweyi,ywayi piny kata rodhi? |  |  |  |  |
| 1. Kuom dweche 12 mosekalo , be jaherani ose goyo, gweyo,ywayo piny kata rodho nyathini? |  |  |  |  |
| 1. Kuom dweche 12 mosekalo, be jaherani osechuni mondo uriwru kode to ne ok idwa? |  |  |  |  |

PMTCT Knowledge

Kiyie to yier duoko maberni

1. Ndalo ma a yach, onego ayud pim mar viral load
2. Adagi,pim mar viral load itimo mana bang nywol
3. Kind achiel kod nyadiriyo
4. Nyadidek.
5. Ok angeyo
6. Ndalo ma I yach, dwarre ni mon man kod kute mag ayaki omwony yath mar ART seche ma ok giwinj maber………………………Adier / miriambo
7. Dwarore ni mondo adhi clinic mar ANC nyadidi?
8. 2
9. 3
10. 4
11. 5

PMTCT Motivation and self-efficacy

Kiyie to nyis kaka iyie kata idagi kod weche gi

|  | Adagi Ahinya | Adagi | Man e diere | Ayie | Ayie Ahinya |
| --- | --- | --- | --- | --- | --- |
| 1. Angeyo ni anyalo luwora kod thieth kaka dwarore ndalo duto ma ayach, kata ka ji nitiere machiegni. |  |  |  |  |  |
| 1. En gima ber mondo adhi anywol e od thieth moloyo nywol dala. |  |  |  |  |  |
| 1. Chunya chandore ni ok abiyudo gima biro ting’a ka tera e od thieth ka saa mar nywol ochopo |  |  |  |  |  |
| 1. Dich moko machalo kaka tich, tije ot, rito nyathi nyalo mona mondo adhi e thieth mar ANC kod PMTCT. |  |  |  |  |  |
| 1. Dhi e thieth mar ANC/PMTCT en gima ber ahinya kuomi |  |  |  |  |  |

|  | Adagi ahinya | Adagi | Man e dierel | Ayie | Ayie Ahinya |
| --- | --- | --- | --- | --- | --- |
| 1. Ne adeko biro e od thieth mondo ayud ANC nikech weche Covid_19 |  |  |  |  |  |
| 1. Ne adeko biro yudo nikech ngato e oda, kata an ne ok awinj maber, bende ne ok adwar ni mondo amii ngato tuo. |  |  |  |  |  |
| 1. Ok adwar biro e od thieth sani ahinya mondo Ayud thieth mar ANC nikech aluor yudo tuo mar Covid-19 |  |  |  |  |  |
| 1. Chunya ne chandre ni kadipo ni anywol e od thieth to tuo mar covid -19 nyalo maka kata nyathina |  |  |  |  |  |
| 1. Wuoth mar biro e od thieth osebedo matek( kalure kod pesa mar wuoth, kata yudo yor wuoth kaka gari kata apiko) nikech weche covid 19 |  |  |  |  |  |

Erokamono ahinya kuom miwa saa mari kod duoko penjo.

**Appendix IIB. Delivery survey** (within 4 weeks, Intervention and Control Sites)

***For interviewer to fill out:***

Study ID: ______________

Date of interview__________________________

Interviewer initials_________________________

Delivery

1. Nyathini ne inyuolo kanye?
   1. Od thieth
      1. Mane, kon mane ___________________
   2. Nyamrerwa
      1. Mane,kon mane _______________
   3. Dala
   4. Mamoko
      1. Kiyie to ler ane_________
2. Ne okawi seche adi mondo inywol**?**
   1. Matin ne seche 2
   2. Seche 2-5
   3. Seche 6-12
   4. Mohingo seche 12
3. Bende in kata nyathi ne oyudo chadruok moro amora**?**
   1. Eeh
      1. Kiyie to ler ane gimane otimore ne mama kod nyathi yore: ________________________

_________________________________________________________________

- 1. Ooyo

Partner status & Disclosure

1. Chal mari mar keny**?**
   1. Pok adi katedo
   2. Ose kenda
   3. Pok okenda to an an kod osiepna
   4. Wapogore kod jaherana
   5. Wawere kod jaherana
   6. Chi liel
   7. Mamoko, ler ane__________
2. Dak mari chal nade?
   1. Aonge jaherana sani
   2. Adak mopogore kod jaherana
   3. Adak kamoro achiel kod jaherana
3. Jaherani ma sani e wuon nyathi?...........Eeh / Ooyo
4. Chal mari mar ayaki isenyiso ng’a??
   1. Onge ng’ato ma asenyiso
   2. Jaoda/jaherana
   3. Ng’ato achiel kase anyuola
   4. Ng’ato achiel kase osiepni
   5. Ng’ato achiel kose janyuolni
5. Chal mar ayaki mar jaherani?
   1. En kod kute mag ayaki
   2. Oonge kod kute mag ayaki
   3. Ok ong’ere
   4. Aonge ng’ato sani
6. En ng’a ma chiwo yore kaka onego uti kod pesa e odu?
7. Jaherana, seche duto
8. Jaherana ngenyne ma oloya
9. Jaherana kod an maromre
10. An ngenyne moloyo jaherana
11. An, Seche duto.
12. Aonge jaherana sani
13. Yuto maru e juma
14. Tin ne mia 500
15. Kind mia 500-750
16. Kind mia 750-1,000
17. Kind mia 1,000-2,500
18. Oingo 2,500
19. Chunya chandre nikech aonge pesa moromo ma anyalo chopo godo hospital kata ma nyathina nyalo yudo godo thieth?
    1. Ayiego Ahinya
    2. Ayiego
    3. Adagi
    4. Adagi Ahinya

Partner Support

1. Ndalo mane iyach jaherani ma dichwo bende nekonyi e yore gi, kaka?:

|  | Podi | Ok ahinya | Seche moko | Ngenyne |
| --- | --- | --- | --- | --- |
| 12b. Miyi pesa ma iting’i godo ka idhi e od thieth? |  |  |  |  |
| 12c. Miyi paro kaka inyalo bedo gi ngima maber ndalo ma iyach? |  |  |  |  |
| 12d Konyi kod tije mag ot mondo iywe? |  |  |  |  |
| 12e. Konyi kod tije mag ot mondo idhi e thieth mar PMTCT? |  |  |  |  |
| 12f. Paroni mondo imwony yath. |  |  |  |  |
| 12g. Paroni mondo iluw thieth kaka daktari ne owacho. |  |  |  |  |
| 12h. Miyi pesa mar nyiew yath kata vitamins? |  |  |  |  |
| 12i. Chuora ne omiya mijingo ndalo mane a yach |  |  |  |  |
| 12j. An kod chuora ne walemo kanyakla mondo atiek ich maber kendo mondo anyuol maber |  |  |  |  |

1. Bende ne udhi kod jaherani e thieth mar PMTCT kamoro achiel?
   1. Ooyo, ne ok odhi kat achiel
   2. Ne odhi dichiel
   3. Ne odhi diriyo
   4. Ne odhi moloyo adek.
   5. Aonge jaherana.
2. Ka #13 en Ooyo, en ang’o momiyo ne ok odhi?
   1. Ne entie e tich seche mane adhi e PMTCT__________Eeh / Ooyo
   2. Odak kendo otiyo mabor _______________________ Eeh / Ooyo
   3. Chunye ne ok orwako bedo e od thieth____________ Eeh /Ooyo
   4. Ne ok adwar ni mondo odhi ____________________ Eeh / Ooyo
   5. Ne ok odwar dhi _____________________________ Eeh / Ooyo
   6. Ne en kod tije mamoko ________________________Eeh / Ooyo
   7. Mamoko, ler ane_________________________________
3. Be jaherani madichwo ne okonyi sama ne idhi nyuol? Kiyie to yier dwoko miyiego.
   1. Aonge kod jaherana madichwo
   2. Ooyo,Jaherana ma dichwo ne ok ochiwo kony moro a mora seche mane adhi nyuol.
   3. Jaherana ma dichwo ne okonya kod pesa mar chopo hospital, ento ne ok ochopo.
   4. Jaherana ne okowa e hospital seche mane muoch kaya.
   5. Jaherana ne obiro nena e hospital bang ka ose nyuol nyathi.
   6. Jaherana ne okowa e hospital seche mane muoch kaya kendo ne obiro nena e hospital bang ka ose nyuol nyathi

Depression

Kiyie to yier dwoko machiegni gi kaka ne iwinjo kuom ndalo 7 mosekalo,ok kawuono kende.

1. Ase ngiyo mbele kod mor mar gik mangeny
2. Kaka asebedo ga katimo
3. Matin kaka asebedo katimo
4. Matin ahinya moloyo kaka asebedo
5. Ak asebedo
6. Weche ka odhi marach to asebedo ka ajilaumu
7. Eeh, Ngenyne
8. Eeh , seche moko
9. Ok seche te
10. Ooyo, podi
11. Asebedo ka awinjo ka aluor ma onge gima omiyo
12. Eeh,seche mangeny
13. Eeh, seche moko
14. Ooyo, ok ahinya
15. Ooyo,podi
16. Asebedo ma ok amor, ma asebedo ka aywak
17. Eeh, seche mangeny
18. Eeh, seche moko
19. Mana seche moko
20. Ooyo,podi
21. Paro mar hinyruok an awuon ase bedogodo
22. Eeh, Ngenyne
23. Seche moko
24. Mana seche moko
25. Podi

Risk of Violence

Kuom weche gi chakre (19-22), ), yier duoko manyiso kaka gik moko osetimre kuom dweche 12 mosekalo.

|  | Podi | Ok Ahinya | Seche Moko | Ngenyne |
| --- | --- | --- | --- | --- |
| 1. Kuom dweche 12 mosekalo, be jaherani ose yanyi kata miyi mondo iwinj marach ne in iwuon? |  |  |  |  |
| 1. Kuom dweche 12 mosekalo , be jaherani ose goyi, gweyi,ywayi piny kata rodhi? |  |  |  |  |
| 1. Kuom dweche 12 mosekalo , be jaherani ose goyo, gweyo,ywayo piny kata rodho nyathini? |  |  |  |  |
| 1. Kuom dweche 12 mosekalo, be jaherani osechuni mondo uriwru kode to ne ok idwa.? |  |  |  |  |

PMTCT Knowledge

1. Bende ng’a mamiyo nyalo gengo nyathine kuom yudo kute mag ayaki e yore gi?

a. Muonyo yedhe duto mag ART ndalo ma iyach kod ka I dhodho nyathi....................................Yes / No / DK

bDhi nyuol e hospital maloyo nyuol dala……………....................................Yes / No / DK

c. Tiyo kod infant formula seche madwarore kod dhodho nyathi ………………..Yes / No / DK

d. Miyo nyathi nevirapine…………………………..........................................................Yes / No / DK

e Miyo nyathi Cotrimoxazole…………………………………………………..…………...Yes / No / DK

e. Miyo nyathi chak mar dhiang kod tong kapok ochopo dweche auchiel mondo omi nyathi odong kotegno…Yes / No / DK

1. Dwarore ni nyathi oyud pim makwongo mar ayaki kaen
2. Ei wige ariyo bang nyuol
3. Ka en wige auchiel
4. Ka en kod dweche auchiel
5. Ka en ja dweche apar kod ariyo
6. Ka oseweyo dhoth
7. Okya
8. Dwarore ni nyathi mondo opim kute mag ayaki seche madwarore nyaka ochop kara ang’o”:
   1. Dweche auchiel
   2. Dweche apar kod ariyo
   3. Dweche apar kod aboro
   4. Higni ariyo
   5. Ka nyathi oweyo dhoth

PMTCT Motivation

For each of the following, indicate the extent to which you agree or disagree with the statement.

SD=strongly disagree, D=disagree, N=neither agree nor disagree, A= agree, SA = strongly agree.

|  | Adagi Ahinya | Agadi | Man e diere | Ayie | Ayie Ahinya |
| --- | --- | --- | --- | --- | --- |
| 1. Angeyo ni anyalo luwora kod thieth kaka dwarore seche ma nyathina pod tin kendo dhoth |  |  |  |  |  |
| 1. Miyo nyathi yathe en gima maber kuoma |  |  |  |  |  |
| 1. Kata ka nyathi ok dwa yath, to angeyo ni abiro miye yath kaka dwarore |  |  |  |  |  |
| 1. Ber mondo nyatho oyud pim mar viral seche duto ndalo mapod odhoth. |  |  |  |  |  |
| 1. Dich moko machalo kaka tich, tije ot, rito nyathi nyalo mona mondo oyud pim mar wige auchiel. |  |  |  |  |  |
| 1. Tero nyathi e thieth seche madwarore en gima ger ahinya. |  |  |  |  |  |

|  | Strongly Disagree | Disagree | Neutral | Agree | Strongly Agree |
| --- | --- | --- | --- | --- | --- |
| 1. Chunya ne chandre ni kadipo ni anywol e od thieth to tuo mar covid -19 nyalo maka kata nyathina |  |  |  |  |  |
| 1. Chunya chandre ni ka atero nyathina e pim e od thieth to wanyalo yudo tuo mar covid -19 |  |  |  |  |  |
| 1. Ndalo mane ayach,weche mag wuoth mondo adhi ayud thieth mar ANC ne netek(weche pesa mar wuoth, kata yudo gari/apiko) nikech weche covid 19 |  |  |  |  |  |
| 1. Ndalo mane ayach, ne adeko, kata ne ok adhi a thieth mar ANC, kata dok kaw yath nikech ne ok awinj maber, kendo ne ok adwar ni mondo ami ji tuo. |  |  |  |  |  |

ENGAGEMENT WITH HITSYSTEM - **INTERVENTION ONLY**

1. Nyaka ne ichak dhi e ANC, bende iseyudo sms moro a mora ka oa hospital?
2. Ooyo,Pok ayudo sms.
3. Eeh, Aseyudo achiel, to ma ok ngeny ne adek
4. Eeh, aseyudo kind sms 3 kod 5,
5. Eeh, Aseyudo sms mohewo 5
6. Bende iseyudo sms moro a moro mapenji wechegi?
7. Ngeyo kaka idhi…………………………………….Yes / No
8. Manyisi chieng m onego iduogie e hospital………………… Yes / No
9. Maparoni mondo imwony yath……………………… Yes / No
10. Maparoni mondo iter nyathi e hospital………………Yes / No
11. Manyisi mondo idog e hospital mondo inge chal dwako nyathi………… Yes / No
12. Kane I yie ne iyudo sms e no 34, kiyie to dwokie penjo no 36 kaka ne itimo bang yudo sms, ka ok kamano to dhie no 37:
13. Sms go ne ok okonya……………….……………………………….………..True / False
14. Sms ne okonya mondo apar amwony yath……………………..…..True / False
15. Ne an kod chandruok ni ngato nyalo neon sms ma ng’e godo chal mara…….True / False
16. Sms ne omiya abedo kod e achiel gi hospital/jo thieth…….True / False
17. Sms ne omiyo anyalo pare chieng ma onego adogie e hospital……………………True / False
18. Ng’ato ma opgre koda ne osomo sms ma openja……………True / False
19. Ja family kata osiepna ne ongeyao chal mara nikech sms no………True / False
20. Sms ne omiya weche mabeyo kaluwore kod thieth mar nyathina……True / False
21. Sms ne omiya mijingo mondo amend dhi mbele kod yudo thieth mar PMTCT/EID………………………True / False
22. Kiyie to yier seche ma diher ni mondo waparni godo seche ma onega imwony ART
23. Okadwar,, kik uorna sms
24. Pile
25. Juma
26. Bang juma 2
27. Dwe ka dwe

Erokamono ahinya kuom miyowa saa mari kod duoko penjo.

**Appendix IIC. 6 Month Postpartum survey (Intervention and Control Sites)**

***For interviewer to fill out:***

Study ID: ______________

Date of interview__________________________

Interviewer initials_________________________

Partner status & Disclosure

1. **Kit keny mari en mane?**
2. Pok adhi katedo
3. Ose kenda
4. Pok okenda to an an kod jaherana
5. Wapogore kod jaherana
6. Wawere kod jaherana
7. An Chi liel
8. Dak mari chal nade?
9. Aonge jaherana sani
10. Adak mopogore kod jaherana
11. Adak kamoro achiel kod jaherana
12. Chal mari mar ayaki isenyiso ng’a?
13. Onge ng’ato ma asenyiso
14. Jaoda/jaherana
15. Ng’ato achiel kase anyuola
16. Ng’ato achiel kase osiepni
17. Ng’ato achiel kose janyuolni
18. Chal mar ayaki mar jaherani?
19. En kute mag ayaki
20. Oonge kod kute mag ayaki
21. Ok Ong’ere
22. Aonge jaherana sani
23. Jaherani ma sani e wuon nyathi? ….. Eeh / ooyo/ NA
24. En ng’a ma chiwo yore kaka onego uti kod pesa e odu?
25. Jaherana, seche duto
26. Jaherana ngenyne ma oloya
27. Jaherana kod an maromre
28. An ngenyne moloyo jaherana
29. An, Seche duto.
30. Aonge jaherana sani
31. Yuto maru e juma?
32. Tin ne mia 500
33. Kind mia 500-750
34. Kind mia 750-1,000
35. Kind 1,000-2,500
36. Oingo 2,500
37. Chunya chandre nikech aonge pesa moromo ma anyalo chopo godo hospital kata ma nyathina nyalo yudo godo thieth?
38. Ayiego Ahinya
39. Ayiego
40. Adagi
41. Adagi Ahinya

Partner Support (To be filled at 6 month VL test)

1. Nyaka ne i nyuol bende jaherani ose chung kodi e yore gi?:

|  | Podi | Tinne | Seche moko | Pile pile |
| --- | --- | --- | --- | --- |
| 9a. Dhi e thieth mar nyathi sama dwarore? |  |  |  |  |
| 9b. Miyi pesa mar dhi godo hospital? |  |  |  |  |
| 9c. Miyi paro kaka inyalo pidho nyathi kata keto ler? |  |  |  |  |
| 9d. Konyi kod tije ot mondo iyue? |  |  |  |  |
| 9e. Paroni mondo imi nyathi yath? |  |  |  |  |
| 9f. Paroni mond imwony yath? |  |  |  |  |
| 9g Paroni mondo iluw thieth kaka daktari ne owacho? |  |  |  |  |
| 9h.Miyi pesa mar nyiew yath kata vitamins? |  |  |  |  |
| 9i. Chiwo mij’ingo mondo I dag e ngima maber? |  |  |  |  |
| 9j. Lemo kanyakla mondo ubed kod ngima maber? |  |  |  |  |

Depression

Kiyie to yier dwoko machiegni gi kaka ne iwinjo kuom ndalo 7 mosekalo,ok kawuono kende

1. Asebedo gi geno kod ilo ne gik moko
2. En mangey kaka ase timo
3. Matin ahinya kaka asetimo
4. Matin moromo
5. Ok asebedo
6. Weche ka odhi marach to asebedo ka ajilaumu
7. eeh seche te
8. seche moko
9. ok seche te
10. ooyo podi
11. Asebedo ka awinjo ka aluor ma onge gima omiyo
12. Eeh,seche mangeny
13. Eeh, seche moko
14. Ooyo, ok ahinya
15. Ooyo,pod
16. Asebedo ma ok amor, ma asebedo ka aywak
17. Eeh, seche mangeny
18. Eeh, seche moko
19. Mana seche moko
20. Ooyo,podi
21. Paro mag hinyruok an awuon ase bedogodo
22. Eeh, Ngenyne
23. Seche moko
24. Mana seche moko
25. Podi

Risk of Violence

Kuom weche gi chakre (15-18), yier duoko manyiso kaka gik moko osetimre kuom dweche 12 mosekalo?

|  | Podi | Tinne | Seche moko | Pile pile |
| --- | --- | --- | --- | --- |
| 1. Kuom dweche 12 mosekalo, be jaherani ose yanyi kata miyi mondo iwinj marach ne in iwuon? |  |  |  |  |
| 1. Kuom dweche 12 mosekalo , be jaherani ose goyi, gweyi,ywayi piny kata rodhi? |  |  |  |  |
| 1. Kuom dweche 12 mosekalo , be jaherani ose goyo, gweyo,ywayo piny kata rodho nyathini? |  |  |  |  |
| 1. Kuom dweche 12 mosekalo, be jaherani osechuni mondo uriwru kode to ne ok idwa? |  |  |  |  |

PMTCT Knowledge

1. Mine man kod tuo mar ayaki, ma kute gi okyanl fweny onego opim nyadidi ndalo ma gi dhodho nyathi?
2. Bang dweche adek
3. Bang dweche auchiel
4. Bang dweche apar kod ariyo
5. Mana, seche ma nyathi oweyo dhoth
6. Mine man kod kute mag ayaki onego we mwonyo yath mar ART karang’o?
7. Nyaka nyathi chak chamo chiemo mapek, ka osechopo dweche auchiel.
8. Nyaka nyathi we dhoth.
9. Nyaka yudni nyathi onge kute mag ayaki bang pim ka osechopo dweche 18
10. Ooyo, min nyathi onego dhi mbele kod mwonyo yath mondo orit ng’imane.
11. Dwarore ni nyithindo mondo opim kute mag ayaki seche madwarore nayaka girom nade?:
12. Dweche auchiel
13. Dweche apar kod ariyo
14. Dweche apar kod aboro
15. Higni ariyo
16. Ka nyathi oweyo dhoth

PMTCT Motivation

Kuom weche gi, ere ma iyiego kata idagi.

SD=strongly disagree, D=disagree, N=neither agree nor disagree, A= agree, SA = strongly agree.

|  | Adagi Ahinya | Adagi | Man e diere | Ayie | Ayie Ahinya |
| --- | --- | --- | --- | --- | --- |
| 1. Angeyo ni anyalo luwora kod thieth kaka dwarore seche ma nyathina pod tin kendo dhoth |  |  |  |  |  |
| 1. Miyo nyathi yathe en gima maber kuoma |  |  |  |  |  |
| 1. Kata ka nyathi ok dwa yath, to angeyo ni abiro miye yath kaka dwarore |  |  |  |  |  |
| 1. Ber mondo nyatho oyud pim mar viral seche duto ndalo mapod odhoth |  |  |  |  |  |
| 1. Dich moko machalo kaka tich, tije ot, rito nyathi nyalo mona mondo oyud pim mar dweche auchiel. |  |  |  |  |  |
| 1. Tero nyathi e thieth seche madwarore en gima ger ahinya |  |  |  |  |  |

|  | Adagi ahinya | Adagi | Man e dierel | Ayie | Ayie Ahinya |
| --- | --- | --- | --- | --- | --- |
| 1. Chunya chandre ni ka atero nyathina e pim e od thieth to wanyalo yudo tuo mar covid -19 |  |  |  |  |  |
| 1. Weche mag wuoth dhi kendo duogo ka aa e od thieth(weche pesa kata yudo gari/apiko) kalure kod tuo mar covid 19 ne omiyo adeko, kata koso yudo thieth |  |  |  |  |  |
| 1. Ne adeko yudo thieth kata dhi e od thieth mondo,nikech ne atuo kendo ne ok adwa ni mondo ami ji tuo |  |  |  |  |  |

Assessing delivery VL as intervention

Kiyie to nyiswa Tarik kod higa mane remo mar VL ne okau, dwoko mag pim, to kod gima omiyo ne otim pim are nyaka ne ibed mayach. Kiyie to we nono kar dwoko penjo ka remo ne ok okau.

1. Pim mokwongo nyaka ne yud ni in kod ich
2. **Tarik mar pim mokwongo** _______________________
3. Dwoko mokwongo( yier achiel)
4. Ofwenyore
5. Ok ofwenyore
6. Ne ayudo dwoko, to ok apar ni ne en nade
7. Ne ok onyisa dwoko
8. Gima omiyo ne otim pim
9. Pim ma pile mar VL
10. Iluwo godo pim mar VL mane ofwenyore
11. Nonro
12. Ok ang’eyo
13. Pim mar ariyo nyaka ne yud ni in kod ich
14. **Tarik mar pim mar ariyo** _______________________
15. Dwoko mar ariyo(yier achiel)
16. Ofwenyore
17. Ok ofwenyore
18. Ne ayudo dwoko, to ok apar ni ne en nade
19. Ne ok onyisa dwoko
20. Gima omiyo ne otim pim
21. Pim ma pile mar VL
22. Iluwo godo pim mar VL mane ofwenyore
23. Nonro
24. Ok ang’eyo
25. Pim mar adek nyaka ne yud ni in kod ich
26. **Tarik mar pim mar adek** _______________________
27. Dwoko mar adek (yier achiel)
28. Ofwenyore
29. Ok ofwenyore
30. Ne ayudo dwoko, to ok apar ni ne en nade
31. Ne ok onyisa dwoko
32. Gima omiyo ne otim pim
33. Pim ma pile mar VL
34. Iluwo godo pim mar VL mane ofwenyore
35. Nonro
36. Ok ang’eyo
37. Pim mar angwen nyaka ne yud ni in kod ich
38. **Tarik mar pim mar angwen** _______________________
39. Dwoko mar angwen (yier achiel)
40. Ofwenyore
41. Ok ofwenyore
42. Ne ayudo dwoko, to ok apar ni ne en nade
43. Ne ok onyisa dwoko
44. Gima omiyo ne otim pim
45. Pim ma pile mar VL
46. Iluwo godo pim mar VL mane ofwenyore
47. Nonro
48. Ok ang’eyo

Engagement with HITSystem 2.1 - **INTERVENTION ONLY**

1. Nyaka ne ichak dhi e ANC, bende iseyudo sms moro a mora ka oa hospital?
2. Ooyo,Pok ayudo sms.
3. Eeh, Aseyudo achiel, to ma ok ngeny ne adek
4. Eeh, aseyudo kind sms 3 kod 5,
5. Eeh, Aseyudo sms mohewo
6. Bende iseyudo sms moro a moro mapenji wechegi?
7. Ngeyo kaka idhi…………………………………….Yes / No
8. Manyisi chieng ma onego iduogie e hospital………………… Yes / No
9. Maparoni mondo imwony yath……………………… Yes / No
10. Maparoni mondo iter nyathi e hospital………………Yes / No
11. Manyisi mondo idog e hospital mondo inge chal dwako nyathi………… Yes / No
12. Kane I yie ne iyudo sms e no 33, kiyie to dwokie penjo no 34 kaka ne itimo bang yudo sms, ka ok kamano to dhie no 35:
13. Sms go ne ok okonya……………….……………………………….………..True / False
14. Sms ne okonya mondo apar amwony yath……………………..…..True / False
15. Ne an kod chandruok ni ngato nyalo neon sms ma ng’e godo chal mara…….True / False
16. Sms ne omiya abedo kod e achiel gi hospital/jo thieth…….True / False
17. Sms ne omiyo anyalo pare chieng ma onego adogie e hospital……………………True / False
18. Ng’ato ma opgre koda ne osomo sms ma openja……………True / False
19. Ja family kata osiepna ne ongeyao chal mara nikech sms no………True / False
20. Sms ne omiya weche mabeyo kaluwore kod thieth mar nyathina……True / False
21. Sms ne omiya mijingo mondo amend dhi mbele kod yudo thieth mar PMTCT/EID………………………True / False
22. Kiyie to yier seche ma diher ni mondo waparni godo seche ma onego imwony ART
23. Okadwar,, kik uorna sms
24. Pile
25. Juma
26. Bang juma 2
27. Dwe ka dwe

**Appendix IIF. Data Collection Tool For Home-based Sample Collection**

**For all home-visits:**

1. ID Ja chiwre e nonro _______________
2. Tarik mar nonro_________________
3. Nying Ja nonro ____________
4. Utimo limbe nang’o?
   1. Pim VL mar nyuol
   2. Pim VL mar dweche auchiel
   3. Mamoko,ler ane____________
5. Jotijwa osetimo limbe e dalani kae nyadidi?
   1. Achiel
   2. Ariyo
   3. Adek
   4. Ang’wen kata mangeny
6. Ja chiwre e nonro be oyudre?
   1. Dala
   2. Oonge dala
   3. Odar odhi kama chielo
   4. Ne osetho
7. Be nitie ng’ato e dala (ma opogore kod nyathi ngat ma obi lim)?
   1. Eeh, Jahera ja chiwre e nonro
   2. Eeh, Nyithind ja chiwre e nonro
   3. Eeh, Anyuola ja chiwre e nonro (ng’a giri ___________)
   4. Eeh, osiep kata jirani ja chiwre e nonro
   5. Ooyo, ja chiwre e nonro nitie kende
8. Ka iyie ni eeh e (a, b, c, or d) e penjo mar 7: Be isenyiso jogi duto mantie e dalani chal mari mar ayaki?
   1. Eeh
   2. Ooyo
   3. Ok ong’ere

***Ka ng’ato nitie e dala ma opogre kod ja chiwre e nonro kod nyathine, to ne pok onyiso ng’ato a ng’ata chal mare mar ayaki, weche ma iye kendo mopondo nyalo ngere. Yier chieng machielo mar timo pim mar VL kod EID , to ka ber ne ni weche ma opondo mag ja chiw nonro ok ongere kata matin. Dhi nyime kod penjo (penjo 9-14).***

***Ka ngato onge e dala to ja chiw nonro nitie kod nyathine, kata ka jachiw nonro asenyiso ji e dala chal mare mar ayaki,kare koro dhi nyime kod penjo 15-20***

**Well Baby Visit (if other person is present/confidentiality is compromised):**

1. MUAC mar nyathi ____________cm
2. Liet mar nyathi _______________^o^ Celcius
3. Nyathi dhoth nade?
   1. Maber
   2. Maber matin
   3. Marach
4. Be nitie wach moro amora kod dhoth mar naythi? _______________________________________________ ____________________________________________________________________________________________________________________________________________________________
5. Be nitie wach amora kod ngima nyathi? ___________________________________________________ ____________________________________________________________________________________________________________________________________________________________
6. Be nitie wach moro amora kod ngimani? ________________________________________________ ____________________________________________________________________________________________________________________________________________________________

**Home visit for VL collection (if confidentiality is secure)**

1. Be remo mar VL ne okaw……………………………. Eeh/ Ooyo

14a. Ka en Ooyo, Ang’o ma omiyo?

- 1. Ja chiwre e nonro ne otamore
  2. Mamoko, Ler ane ______________

1. Be nonro ne otieki………………. Eeh/Ooyo
   1. Ka en Ooyo, Ang’o ma omiyo?
      1. Ja chiwre e nonro ne otamore
      2. Mamoko, Ler ane ______________________
2. Ja chiwre e nonro kod nyathine sani yudo thieth mar ayaki Kanye?
3. E od thieth ma itimo e nonro
4. E od thieth machilo, kiyie ti ler ane ni en mane__________________
5. Kod ajuoga
6. Noweyo thieth
7. Mamoko, mage ______________________________
8. Bende nyathi ne opim e od thieth kaka dwarore seche mane hike oromo(6 wks or 6 months)?........... Eeh / Ooyo
9. **Ka en Eeh #18**, od thieth mane ___________________________
10. **Ka en ooyo #18**, to EID sample ne okaw kane otim limbe e dala? ………. Eeh / Ooyo
    - 1. Ka en ooyo #20, ang’o ma omiyo?
         1. Ja chiw nonro ne odagi
         2. Nyathi ne onge dala
         3. Mamoko,ler ane________________________
