# Supplementary material for: Evaluating the efficacy of the HITSystem 2.1 to improve PMTCT retention and maternal viral suppression in Kenya: Study protocol of a cluster-randomized trial
Source: PLoS One. 2022 Jul 26;17(7):e0263988. doi: 10.1371/journal.pone.0263988 (PMC9321364; doi:10.1371/journal.pone.0263988)
Supplement: S1 File — (PDF) [file pone.0263988.s004.pdf]

KENYA MEDICAL RESEARCH INSTITUTE

P.O. Box 54840-00200, NAIROBI, Kenya
Tel: (254) 2722541, 2713349, 0722-205901,0733-400003, Fax: (254) (020) 2720030
Email: director@kemri.org, info@kemri.org, Website. www.kemri.org

KEMRI/RES/7/3/1 **April 27, 2020**

TO: **SHARON N. MOKUA**
PRINCIPAL INVESTIGATOR

THROUGH: **DIRECTOR, CPHR**
NAIROBI.

Forwarded 5/05/2020

Dear Madam,

RE: **KEMRI/SERU/CPHR/06/3983 *(RESUBMISSION OF INITIAL SUBMISSION)*: EVALUATING THE IMPACT OF HIV INFANT TRACKING SYSTEM (HIT SYSTEM) ON RETENTION IN PREVENTION-OF-MOTHER TO CHILD TRANSMISSION SERVICES AND MATERNAL VIRAL SUPRESSION IN SIAYA AND MOMBASA COUNTIES, KENYA *(VERSION 3.0 DATED 10.03.2020)***

Reference is made to your letter dated March 18, 2020. The KEMRI Scientific and Ethics Review Unit (SERU) acknowledges receipt of the revised study documents on March 23, 2020 and online submitted application on April 18, 2020.

This is to inform you that the issues raised during the 296th Committee A meeting of the KEMRI Scientific and Ethics Review Unit (SERU) held on **February 18, 2020** have been adequately addressed.

Consequently, the study is granted approval for implementation effective this day, **April 27, 2020** for a period of **one (1) year**. Please note that authorization to conduct this study will automatically expire on **April 26, 2021.** If you plan to continue with data collection or analysis beyond this date, please submit an application for continuation approval to SERU by **March 15, 2021.**

You are required to submit any proposed changes to this study to SERU for review and the changes should not be initiated until written approval from SERU is received. Please note that any unanticipated problems resulting from the implementation of this study should be brought to the attention of SERU and you should advise SERU when the study is completed or discontinued.

Yours faithfully,

ENOCK KEBENEI,
THE ACTING HEAD,
KEMRI SCIENTIFIC AND ETHICS REVIEW UNIT.

CONFIDENTIAL
