# Supplementary material for: Evaluating the efficacy of the HITSystem 2.1 to improve PMTCT retention and maternal viral suppression in Kenya: Study protocol of a cluster-randomized trial
Source: PLoS One. 2022 Jul 26;17(7):e0263988. doi: 10.1371/journal.pone.0263988 (PMC9321364; doi:10.1371/journal.pone.0263988)
Supplement: S2 File — (PDF) [file pone.0263988.s005.pdf]

The University of Kansas Medical Center

Human Research Protection Program

APPROVAL OF SUBMISSION

December 5, 2019

Sarah Kessler
skessler2@kumc.edu

Dear Sarah Kessler:

On 12/5/2019, the IRB reviewed the following submission:

Type of Review:	Modification / Update
Reviewing IRB:	IRB00006196
FWA#:	00003411
IRB#:	STUDY00144753
Title:	Evaluating the HITSystem to improve PMTCT retention and maternal viral suppression in Kenya
Investigator:	Sarah Kessler
IRB ID:	MOD00026113
Funding:	Name: National Institutes of Health
IND, IDE or HDE:	None
Documents submitted for the above review:	• Response Letter<br>• ICF_Simplified Language_CLean.pdf<br>• KEMRI Letter of support for study<br>• Informed Consent Documents_KEMRI format_simplified language<br>• Request to continue protocol review
Special Determinations:	• Children

The IRB approved this submission from 12/5/2019 to 10/24/2020 inclusive.

Your approved documents are stored in the “Documents” tab for this study in the eCompliance system. The IRB stamped consent form(s) can be found under the “Final” column on the right side of the screen. These are the **only** valid versions for documenting informed consent.

If continuing review approval is not granted on or before 10/24/2020, approval of this study expires after that date.

Approval of this research is contingent upon your agreement to:

(1) Adhere to all KUMC Policies and Procedures Relating to Human Subjects, as written in accordance with the Code of Federal Regulations (45 CFR 46).

(2) Ensure that all study personnel are adequately trained for their role on the study.

(3) Maintain current training in human subjects protection and current disclosure of conflicts of interest as required by KUMC policy.

(4) Except where informed consent and HIPAA authorization have been formally waived by the IRB, seek, document and maintain records of informed consent and HIPAA authorization from each prospective subject or his/her legally authorized representative.

(5) Maintain copies of all pertinent information related to the research study including, but not limited to, video and audio tapes, instruments, copies of written informed consent agreements, and any other supportive documents in accordance with the KUMC Research Records Retention Policy.

(6) Report adverse events, non-compliance and other problems to the IRB by submitting a Report of New Information.

(7) Follow the IRB-approved protocol. Submit Modifications to the IRB for any proposed changes from the previously approved project. Changes may not be initiated without prior IRB review and approval, unless a delay in implementation would place subjects at risk.

(8) Submit a Continuing Review to the KUMC IRB before the expiration date. Federal regulations and IRB policies require continuing review of research at intervals appropriate to the degree of risk, but not less than once per year.

For more information on Human Subjects Research Policies or using the eCompliance system, please see our website at: http://www.kumc.edu/compliance/human-research-protection-program/institutional-review-board.html

If you have any questions regarding the human subject protection process, please do not hesitate to contact our office at 913-588-1240 or humansubjects@kumc.edu.

Sincerely,

Emily Swanger
